# Skeleton of an unusual, cat-sized marsupial relative (Metatheria: Marsupialiformes) from the middle Eocene (Lutetian: 44-43 million years ago) of Turkey

**DOI:** 10.1371/journal.pone.0181712

**Published:** 2017-08-16

**Authors:** A. Murat Maga, Robin M. D. Beck

**Affiliations:** 1 Division of Craniofacial Medicine, Department of Pediatrics, University of Washington, Seattle, Washington, United States of America; 2 Department of Anthropology, University of Washington, Seattle, Washington, United States of America; 3 Center for Developmental Biology and Regenerative Medicine, Seattle Children’s Research Institute, Seattle, Washington, United States of America; 4 School of Environmental and Life Sciences, University of Salford, Manchester, Salford, United Kingdom; 5 School of Biological, Earth and Environmental Sciences, University of New South Wales, Sydney, New South Wales, Australia; Monash University, AUSTRALIA

## Abstract

We describe a near-complete, three-dimensionally preserved skeleton of a metatherian (relative of modern marsupials) from the middle Eocene (Lutetian: 44–43 million years ago) Lülük member of the Uzunçarşıdere Formation, central Turkey. With an estimated body mass of 3–4 kg, about the size of a domestic cat (*Felis catus*) or spotted quoll (*Dasyurus maculatus*), it is an order of magnitude larger than the largest fossil metatherians previously known from the Cenozoic of the northern hemisphere. This new taxon is characterised by large, broad third premolars that probably represent adaptations for hard object feeding (durophagy), and its craniodental morphology suggests the capacity to generate high bite forces. Qualitative and quantitative functional analyses of its postcranial skeleton indicate that it was probably scansorial and relatively agile, perhaps broadly similar in locomotor mode to the spotted quoll, but with a greater capacity for climbing and grasping. Bayesian phylogenetic analysis of a total evidence dataset comprising 259 morphological characters and 9kb of DNA sequence data from five nuclear protein-coding genes, using both undated and “tip-and-node dating” approaches, place the new taxon outside the marsupial crown-clade, but within the clade Marsupialiformes. It demonstrates that at least one metatherian lineage evolved to occupy the small-medium, meso- or hypo-carnivore niche in the northern hemisphere during the early Cenozoic, at a time when there were numerous eutherians (placentals and their fossil relatives) filling similar niches. However, the known mammal fauna from Uzunçarşıdere Formation appears highly endemic, and geological evidence suggests that this region of Turkey was an island for at least part of the early Cenozoic, and so the new taxon may have evolved in isolation from potential eutherian competitors. Nevertheless, the new taxon reveals previously unsuspected ecomorphological disparity among northern hemisphere metatherians during the first half of the Cenozoic.

## Introduction

Current fossil evidence indicates that the mammalian clades Metatheria (= Marsupialia plus all fossil taxa more closely related to marsupials than to placentals) and Eutheria (= Placentalia plus all fossil taxa more closely related to placentals than to marsupials) probably diverged at least 160 million years ago (MYA) [1]. This date is broadly congruent with recent molecular clock estimates for the timing of the split between marsupials and placentals [2–5]. During the Mesozoic, metatherians and eutherians were moderately diverse taxonomically [6], but they were restricted to relatively small body sizes (<7 kg) and exhibited limited ecomorphological disparity [6–8]. Metatherians seem to have been affected much more severely than eutherians by the K-Pg mass extinction event [7–9].

From the Palaeocene onwards, eutherians (including placentals) radiated massively, going on to dominate mammalian faunas in most of the world, in terms of both number of species and ecomorphological disparity [10, 11]. Metatherians dispersed to South America in the latest Cretaceous or early Palaeocene [12–14], and they diversified widely in South America, Antarctica and Australia during the Cenozoic [12, 14–19]. In the northern hemisphere, by contrast, the Cenozoic fossil record of Metatheria is characterised by low species diversity and very limited ecomorphological disparity, with northern hemisphere metatherians eventually going extinct during the middle Miocene [10, 20, 21]. A single didelphid marsupial, the Virginia opossum (*Didelphis virginiana*), is currently widespread in North America, the result of dispersal from South America within the last one million years [22]. However, besides the Virginia opossum and a few other didelphid species that occur in Central America, marsupials are now entirely absent from the northern hemisphere (except for a handful of human introductions). The comparative lack of evolutionary success of metatherians in the northern hemisphere during the Cenozoic has often been argued to be because they are competitively inferior to eutherians [23, 24].

Here, we describe a three-dimensionally preserved partial skull and near complete postcranial skeleton of a new metatherian from the middle Eocene (Lutetian) Uzunçarşıdere Formation in central Turkey. Not only is it remarkably well-preserved, it also reveals previously unsuspected ecomorphological disparity among northern hemisphere metatherians during the Cenozoic. We present qualitative and quantitative functional analyses of its craniodental and postcranial morphology, and we test its evolutionary relationships via Bayesian undated and “tip-and-node dating” phylogenetic analyses of a total evidence dataset comprising 259 morphological characters and 9kb of DNA sequence data from five nuclear protein-coding genes. Finally, we discuss the significance of this taxon for understanding the relative success of eutherians and metatherians in the northern hemisphere during the Cenozoic.

## Materials and methods

### Specimen

The specimen described here was discovered and collected by one of us (AMM) from the Uzunçarşıdere Formation in the summer of 2002, during fieldwork with Nizamettin Kazanc_1_ (Ankara Üniversitesi) and Mary Maas (University of Texas at Austin). No permits were required for the described study, which complied with all relevant regulations. The specimen has been deposited as specimen number AÜJM 2002–25 in a permanent repository, namely Ankara Üniversitesi Jeoloji Müzesi (AÜJM) in Ankara, Turkey; the specimen is publically accessible.

### Nomenclatural acts

The electronic edition of this article conforms to the requirements of the amended International Code of Zoological Nomenclature, and hence the new names contained herein are available under that Code from the electronic edition of this article. This published work and the nomenclatural acts it contains have been registered in ZooBank, the online registration system for the ICZN. The ZooBank LSIDs (Life Science Identifiers) can be resolved and the associated information viewed through any standard web browser by appending the LSID to the prefix “http://zoobank.org/”. The LSID for this publication is: urn:lsid:zoobank.org:pub:7A365AFA-3531-466F-98E3-77443A3E4B9E. The electronic edition of this work was published in a journal with an ISSN, and has been archived and is available from the following digital repositories: PubMed Central, LOCKSS.

### CT scanning

Selected elements of AÜJM 2002–25 were scanned at the Small Animal Tomographic Analysis Facility of Seattle Children’s Research Institute using a Skyscan 1076C desktop micro-CT scanner (Bruker, Belgium). Acquisition parameters were as follows: 1.0mm Al filter, 100kV, 100μA, 360° scan with 1.0° rotational steps. At each rotation, two frames were acquired and averaged. Shadow images from the scan were reconstructed as PNG stacks with 0.036mm isotropic voxels using the scanner vendor’s reconstruction software, NRecon. 3D volume rendering of the PNG stacks was carried out using 3D Slicer 4.6.2 [25], with the exception of Fig 16A, which was produced using Drishti 2.6.3 [26].

### Comparative material

As part of this study, comparative material of modern and fossil metatherians was examined in the following collections: Australian Museum, Sydney, Australia (prefix AM); American Museum of Natural History, New York, USA (prefix AMNH); Vertebrate Paleontology Laboratory, Jackson School of Geosciences, The University of Texas at Austin, Austin, Texas (prefix TMM), Smithsonian National Museum of Natural History, Washington DC, USA (prefix USNM). A full list of the specimens examined is given in S1 Text.

### Description

Description of the dentition of AÜJM 2002–25 follows standard terminology for tribosphenic metatherians [6, 27–30]. Description of the cranium draws on a range of studies of metatherian cranial anatomy [27, 31–39]. Description of the postcranium follows studies of metatherians [32, 39–56] and of other therian mammals [57–62].

### Body mass estimate

The dentition of AÜJM 2002–25, with its steep molar size gradient in which m4 is much larger than m1, is unlike that of any living marsupial; thus, we have chosen not to estimate body mass using tooth size [63–65]. Instead, we estimated body mass based on total jaw length using the “dasyuromorphian” dataset of Myers [65], because dasyuromorphians (e.g. *Dasyurus* spp., *Sarcophilus harrisii*) appear to represent reasonable ecomorphological analogues for AÜJM 2002–25. The regression equation calculated by Myers [65] is log_10_(body mass) = –2.722 + (3.207* log_10_[total jaw length]), with a “smearing estimate” of 2.3% and a percentage error of 18%. Both mandibles are slightly damaged anteriorly in AÜJM 2002–25 (the left mandible is slightly more complete, and so was used to measure total jaw length), and so the body mass estimate using this equation is likely to be a slight underestimate.

### Qualitative morphofunctional analyses

Qualitative functional analysis of the dentition follows Muizon and Lange-Badré [66], whilst that of the skeleton follows multiple studies [32, 39, 41, 43–45, 47–49, 51–55, 57–62].

### Quantitative morphofunctional analyses

Zimicz [67, 68] presented five dental indices, modified from previous work by Van Valkenburgh [69], that can be used to quantitatively compare tooth shape in carnivorous metatherians, namely: Relative Grinding Area (RGA) = square root of the area of the talonid of m4/length of m4; Relative Premolar Size (RPS) = width of the largest lower premolar/cube root of body mass in kg; Premolar Shape (PS) = width of the largest lower premolar/length of largest lower premolar; Relative Premolar Length (RPL) = length of largest lower premolar/length of m4; Relative Blade Length (RBL) or Trigonid Length of the Carnassial Molar (TLC) = length of the trigonid of m4/total length of m4. These indices were calculated for AÜJM 2002–25 and compared with 31 other similarly-sized (<10 kg), carnivorously-adapted metatherians (both modern and fossil), based on either published data or our own measurements of specimens. Body mass estimates for modern taxa are from the PanTHERIA database [70]. Body mass estimates for fossil taxa were taken from the literature, or were calculated using published predictive regression equations based on dental measurements.

Values for individual indices calculated for AÜJM 2002–25 were compared to the critical values presented by Zimicz [67, 68], which are as follows: for RGA, <0.5 = hypercarnivore, 0.5–0.8 = mesocarnivore, >0.8 = hypocarnivore; for PS, >0.58 = bone-cracker (or other durophage), <0.58 = non-bone-cracker; for RPS, >2.6 = bone-cracker (or other durophage), <2.6 = non-bone-cracker; for RPL, >0.7 = hypercarnivore, <0.7 **=** mesocarnivore or hypocarnivore; for RBL/TLC, >0.9 = hypercarnivore, 0.8–0.9 = bone-cracker (or other durophage), 0.7–0.8 = mesocarnivore, <0.7 = hypocarnivore. These values of RGA, RPS, PS, RPL and RBL were also subjected to principal component analysis (PCA) in PAST 3.07 [71] to determine where AÜJM 2002–25 and other carnivorously-adapted metatherians fall in dental morphospace. Because the indices are all dimensionless ratios, the analysis used the variance-covariance matrix, rather than the correlation matrix.

The cranium of AÜJM 2002–25 is too incomplete to allow calculation of bite force using the “dry skull” method [72, 73]. Instead, we used the method of Therrien [74], as modified for metatherians by Wilson et al. [75], to calculate relative bite force based on measurements of the mandible. We measured the dorsoventral radius of the mandible in medial view rather than lateral view (as was done by Therrien [74]), because the molar crowns extend much further ventrally (and the roots are partially exposed) in lateral view than in medial view.

Chen and Wilson [76] identified 30 postcranial indices that collectively perform well at correctly identifying locomotor mode of extant small-bodied mammals. Of these, 24 can be calculated for AÜJM 2002–25. We added AÜJM 2002–25 to the “five-locomotor-mode” dataset of Chen and Wilson [76], which comprises 87 modern mammals of known locomotor mode, namely either “Arboreal”, “Scansorial”, “Semiaquatic”, “Semifossorial” and “Terrestrial”. The 24 indices for this combined taxon set were then subjected to phylogenetically flexible discriminant analysis [77, 78] using a modified version of an R script written by L. Schmitz (available here: https://github.com/lschmitz/phylo.fda), using the phylogeny and divergence times of Bininda-Emonds et al. [79] for relationships among the modern taxa, and assuming equal prior probabilities for the five locomotor modes.

### Phylogenetic analysis

To assess the evolutionary relationships of the new taxon, we added it to a modified version of the total evidence dataset of Beck et al. [80]; this includes 33 metatherian terminals, including representatives of all modern marsupial orders, plus key fossil metatherian taxa known from well-preserved craniodental and/or postcranial material, namely *Asiatherium*, *Deltatheridium*, *Pucadelphys*, *Mayulestes*, *Andinodelphys*, *Herpetotherium*, *Djarthia* and a composite Peradectidae terminal that combines character scores from *Peradectes* and *Mimoperadectes*. This dataset comprises 259 morphological characters (of which 27 [= 10.4%] are dental, 78 [= 30.1%] are cranial, 146 [= 56.4%] are postcranial and 8 [= 3.1%] are soft tissue) and 9012 base pairs of sequence data from five nuclear protein-coding genes (*APOB*, *BRCA1*, *IRBP*, *RAG1* and *VWF*; see Beck et al. [37, 80] for full details). We also added the Late Cretaceous stagodontid *Didelphodon vorax*, which shows some dental similarities to taxon described here, with character scores based on the description of Wilson et al. [75] and on isolated tarsals from Dinosaur Park in Canada that were tentatively referred to *Didelphodon* by Szalay [56]. The new taxon could be scored for 98 morphological characters (= 37.8%), of which 19 are dental, 13 are cranial, and 66 are postcranial, whilst *Didelphodon* could be scored for 73 (= 28.2%), of which 24 are dental, 35 are cranial, and 14 are postcranial; character scores for *Anatoliadelphys* and *Didelphodon* are given in S2 Text. As in Beck et al. [37, 80], 49 characters representing plausible morphoclines were specified as ordered (see S2 Text). The enigmatic fossil Australian marsupial *Yalkaparidon* [37] was deleted, as preliminary analyses indicated that its phylogenetic position was highly unstable. The final taxon set comprised 34 fossil and modern metatherian terminals, plus five non-metatherian outgroup terminals (the extant monotremes *Ornithorhynchus* and *Tachyglossus*, the fossil stem-therian *Vincelestes*, and the fossil eutherians *Asioryctes* and *Ukhaatherium*).

The resultant total evidence matrix (available for download as S1 File) was analysed using Bayesian undated and “tip-and-node dating” analysis in MrBayes 3.2.6 [81], following the general approach of Beck et al. [37, 80]. In brief, an appropriate partitioning scheme and set of models was determined for the sequence data using PartitionFinder v1.0.1 [82], using the Bayesian Information Criterion, linked branch lengths and the “greedy” search algorithm, whilst the morphological data was assigned a single Mk model [83], assuming that only parsimony-informative characters had been scored and with a gamma distribution to model rate heterogeneity between sites. For the “tip-and-node dating” analysis, temporal information was incorporated via ages for the terminals (taken from the literature; assumed age ranges for terminals are listed in S3 Text) and offset-exponential age priors on selected nodes [84–87]. A single Independent Gamma Rates (IGR) clock model [84] and a Fossilised Birth-Death (FBD) tree prior that assumed “diversity” sampling [86] were implemented. Because our taxon set was at the level of genera, we assumed that the sampling probability of our modern taxa was 0.25 (= 23 out of a total of 93 currently recognised genera). Both the undated and dated analyses were run 20x10^6^ generations, sampling every 5x10^3^ generations, with each analysis comprising four independent runs of four chains (three “heated,” one “cold”). An appropriate burn-in period was determined using Tracer v1.6 [88]. The post-burn-in trees from the undated analysis were summarised using 50% majority rule consensus in MrBayes, whereas those from the dated analysis were summarised as a maximum clade credibility (MCC) tree using TreeAnnotator v1.8.3, after first converting branch lengths from substitutions per site to millions of years using the perl script burntrees.pl (available here: https://github.com/nylander/Burntrees). Support was assessed using Bayesian posterior probabilities for both analyses, and divergence times from the dated analysis were calculated as median heights (with 95% highest posterior densities used as confidence intervals).

The morphological characters were then mapped onto the undated and dated phylogenies to identify unambiguous synapomorphies (i.e. character state changes that are synapomorphies under both Accelerated Transformation [ACCTRAN] and Delayed Transformation [DELTRAN]) using PAUP* 4.0a152, assuming the maximum parsimony criterion.

## Results

### Systematic palaeontology

Mammalia

Theria

Metatheria

Marsupialiformes

*Anatoliadelphys* gen. nov. urn:lsid:zoobank.org:act:FC51708A-405F-482F-93D7-EAE0A205283A

*Anatoliadelphys maasae* sp. nov. urn:lsid:zoobank.org:act:FB5EB1F2-843F-41B7-B33A-234724EA96B4

#### Etymology

*Anatolia* (Greek): the geographic name for the Asian part of Turkey; *delphys* (Greek): uterus, a common suffix for marsupials and their fossil relatives; *maasae*: in honour of Dr. Mary Maas and her contributions to Paleogene mammalian palaeontology, particularly in Turkey.

#### Holotype

Ankara Üniversitesi Jeoloji Müzesi (AÜJM) specimen 2002–25, which comprises a fragmented partial cranium, both dentaries, and associated postcranial elements, including most of the vertebral column, partial pectoral and pelvic girdles, all of the long limb bones, both calcanei, two metapodials, and a few phalanges.

#### Locality and age

AÜJM 2002–25 was collected from the Lülük member of the Uzunçarşıdere Formation (UCF), which is part of the small Orhaniye-Güvenç sedimentary basin located at the northwestern edge of the city of Ankara, approximately 5 km southwest of the town of Kazan, in central Turkey [89]. The Lülük member is the lowest of the three members currently recognised within the UCF (together with the Gökdere [middle], and Sarıbeyler [upper] members), and is the source of all fossil mammals known from the UCF to date [89–94]. AÜJM 2002–25 is from locality AK33, which is approximately 90m above the base of the UCF, at Memlik village (N40° 5.5914’, E32° 44.3924’). Until recently, the age of the UCF was poorly constrained, but a combination of U-Pb dating of zircons and magnetostratigraphy now support a date of 44–42 MYA (= Lutetian) for the formation as a whole, and 44–43 MYA for the Lülük member [89, 94].

#### Diagnosis

*Anatoliadelphys maasae* differs from all other metatherians in the following combination of features: comparatively large size (estimated body mass 3–4 kg); premolars increase markedly in size posteriorly (occlusal area of p1 less than one sixth that of p3); P3 and p3 very large (similar in occlusal area to M2 and m2 respectively) and also broad (labiolingual width:mesiodistal length ratio is 0.89 for P3 and 0.7 for p3); modified tribosphenic molar dentition, in which M1-3 and m1-4 increase markedly in size posteriorly (occlusal area of M1 approximately one third that of M3; occlusal area of m1 approximately one seventh that of m4); upper molars with cingula extending along the anterior and posterior margins; protocone large but conules indistinct or absent; metacone taller than the paracone on M3 but smaller than the paracone on M4; centrocrista v-shaped on M3, with the premetacrista extending labially to stylar cusp D; centrocrista straight on M4; parastylar lobe very large on M4; anterior cingulid weakly developed on m3-4; m4 trigonid dominated by enormous protoconid, with paraconid and metaconid both greatly reduced; preentocristid and cristid obliqua of m3-4 both with carnassial notch; posterior cingulid present but very faint on m3-4; strongly curved radius and tibia; femur with prominent third trochanter, well-marked trochlea and distal condyles of approximately equal width; calcaneus with medially-inflected tuber, large peroneal process with prominent groove for peroneus longus tendon, concave calcaneocuboid facet, and prominent pit (probably for plantar calcaneocuboid ligament) on ventral surface.

### Description

#### General

**Fig 1:**
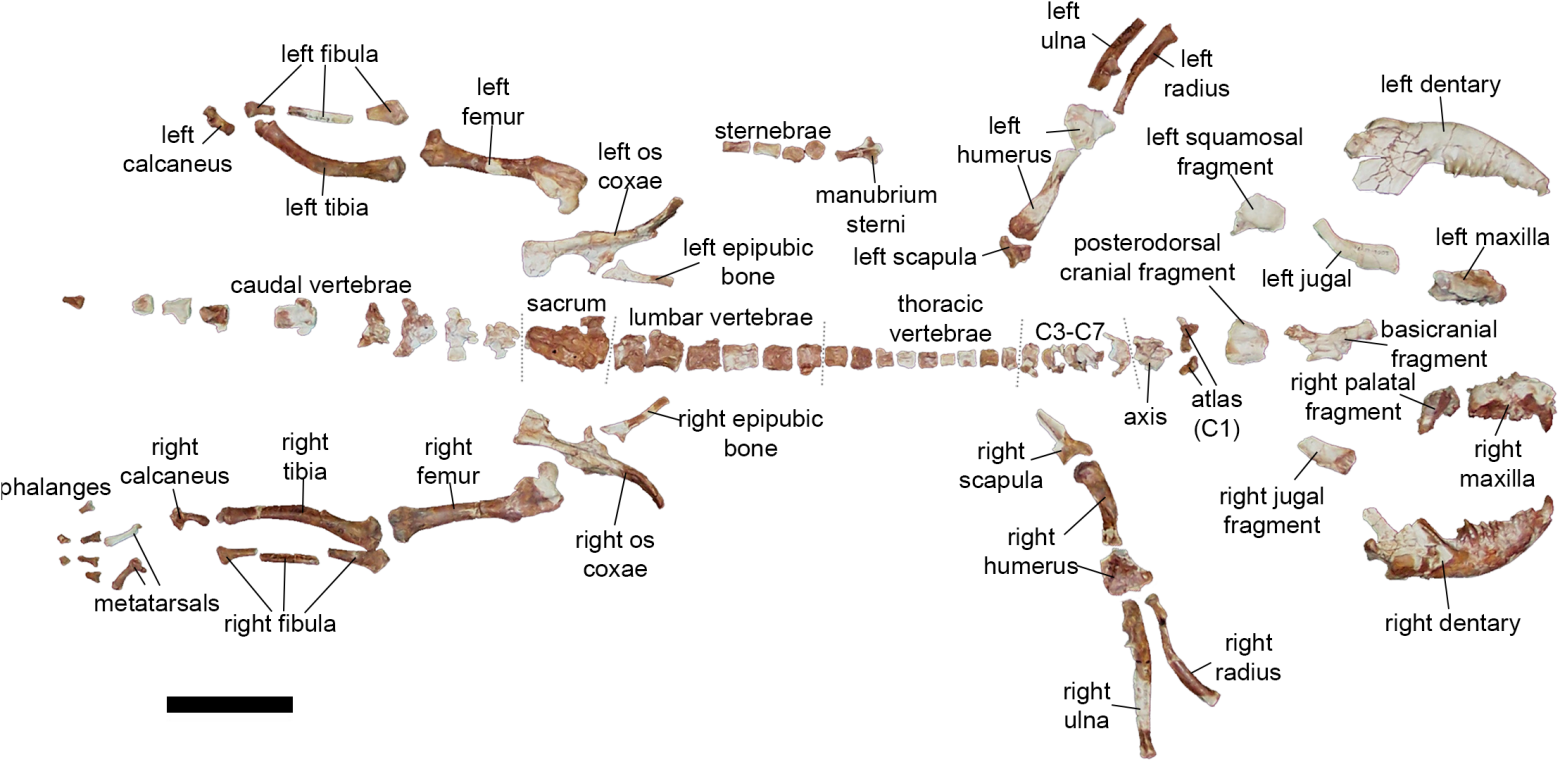
Holotype skeleton of *Anatoliadelphys maasae* (AÜJM 2002–25). Scale bar = 5 cm.

Relative to other fossil metatherian specimens known from the Cenozoic of Laurasia, AÜJM 2002–25 is remarkably well-preserved (Fig 1). It is more complete than skeletal remains of the herpetotheriiid *Herpetotherium* cf. *fugax* that have been described from the early Oligocene (Orellan) of North America [49, 50]. Several fossil metatherian specimens from Eocene deposits at Messel and Geiseltal in Germany are more complete than AÜJM 2002–25, and include some soft tissue preservation, but they are badly crushed [95, 96]; as a result, comparatively little information can be extracted from them. By virtue of its combination of completeness and three-dimensional preservation, AÜJM 2002–25 is a particularly significant specimen.

The following elements are preserved: a partial cranium, broken into multiple fragments but preserving left and right C1 P1-3 M1-4; left and right dentaries, damaged anteriorly but preserving c1 p1-3 m1-4; most of the vertebral column, although many vertebrae are represented by the centrum only; the manubrium sterni and sternebrae; left and right partial scapulae; left and right humeri; left and right ulnae; left and right radii; left and right ossa coxae; left and right femorae; left and right tibiae; left and right fibulae; left and right calcanea; two metapodials; four proximal and two intermediate phalanges. A detailed description of each these elements follows.

#### Dentition as a whole

The upper and lower dentition anterior to the canines is not preserved in AÜJM 2002–25. The specimen does not preserve any direct evidence of the pattern of dental replacement, but the postcanine dentition comprises three premolariform teeth and four molariform teeth in the upper and lower jaws, suggesting the presence of a typical metatherian dental formula of three premolars and four molars [6, 97, 98]. With the exceptions of M3-4 and m3-4, the postcanine teeth are heavily worn, and so aspects of their occlusal morphology are unclear.

#### Upper dentition

**Fig 2:**
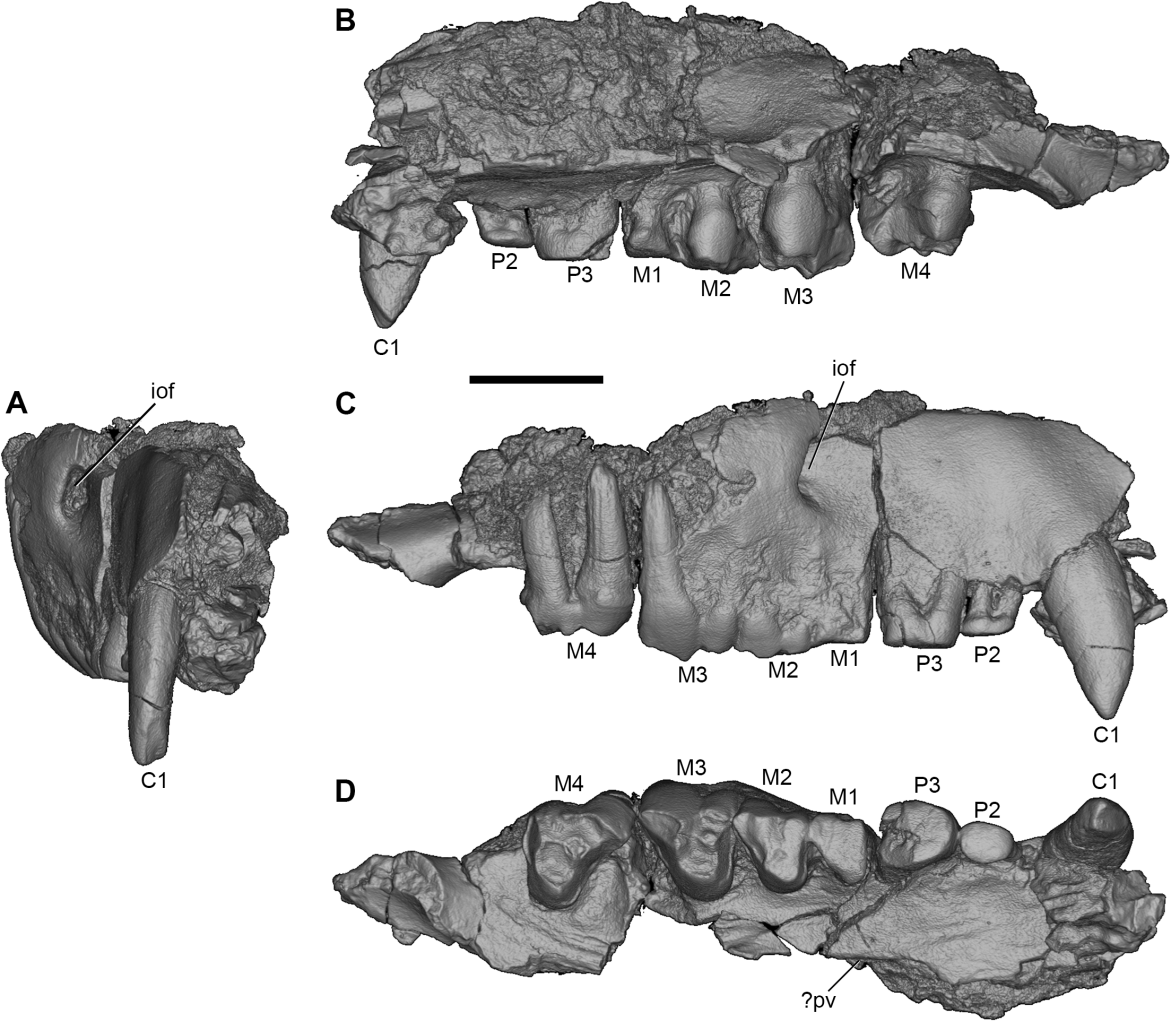
Volume-rendered CT image of right maxilla of holotype of *Anatoliadelphys maasae* (AÜJM 2002–25). C1 P2-3 M1-4 are preserved. Note that the maxillary fragment housing M4 is slightly displaced relative to the intact morphology (the correct position for M4 can be seen in Figs 4 and 5). **A**, anterior view; **B**, medial view; **C**, lateral view; **D**, ventral (occlusal) view. Abbreviations: iof, infraorbital foramen;? pv,? palatal vacuity. Scale bar = 1 cm.

**Fig 3:**
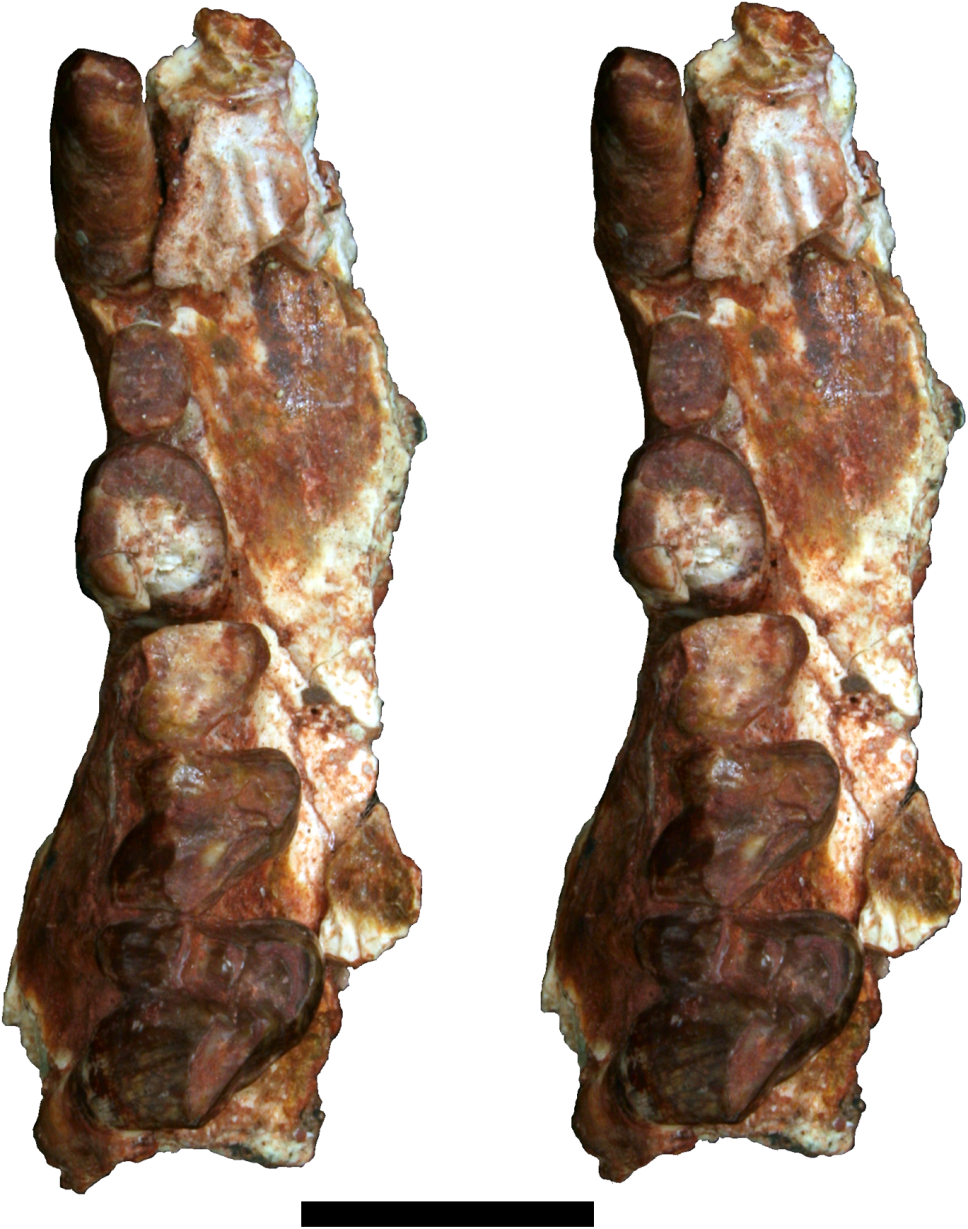
Stereo-photograph of right maxilla of holotype of *Anatoliadelphys maasae* (AÜJM 2002–25) in ventral (occlusal) view. C1 P2-3 M1-3 are preserved. Scale bar = 1 cm.

A large, robust, caniniform, single-rooted C1 is present (Figs 2 and 3). The anteroventral tip of C1 bears a sulcus or wear facet, resulting from contact with c1.

The right maxilla (Figs 2 and 3) retains P2 and P3 *in situ*, and also has a tiny alveolus for P1. P2 is very heavily worn, and little can be inferred regarding its occlusal morphology. P3 is fully erupted but considerably less worn than is P2, suggesting that it erupted much later; M4 is also fully erupted and lightly worn, indicating that AÜJM 2002–25 represents an adult individual. A similar wear pattern is seen in the lower dentition. Collectively, this is consistent with a marsupial-type pattern of dental replacement (in which replacement occurs only at the third premolar locus [6, 99, 100]), and suggests that the third premolars erupted about the same time as the fourth molars; however, other interpretations are possible [6, 101]. P3 is nearly as wide as it is long (labiolingual width:mesiodistal length ratio is 0.87), and is similar in size in terms of occlusal area to M2.

**Fig 4:**
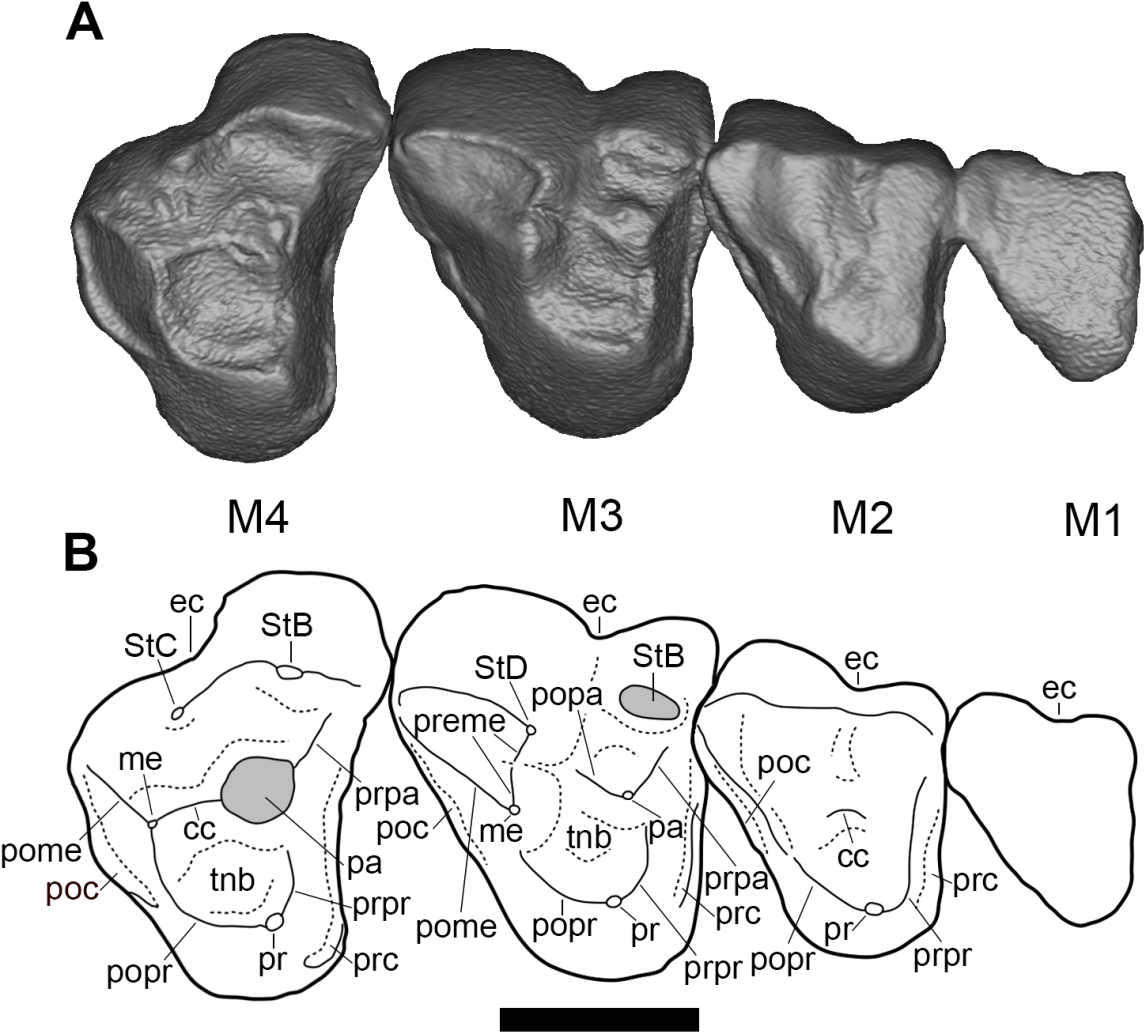
Right upper molars (M1-4) of holotype of *Anatoliadelphys maasae* (AÜJM 2002–25) in ventral (occlusal) view. **A**, Volume-rendered CT image; **B**, interpretative drawing (damaged areas are indicated in grey). Abbreviations: cc, centrocrista; ec, ectoflexus; poc, postcingulum; pome, postmetacrista; popa, postparacrista; popr, postprotocrista; pr, protocone; prc, precingulum; prme, premetacrista; prpa, preparacrista; prpr, preprotocrista; StB, stylar cusp B; StC, stylar cusp C; StD, stylar cusp D; tnb, trigon basin. Scale bar = 0.5 cm.

**Fig 5:**
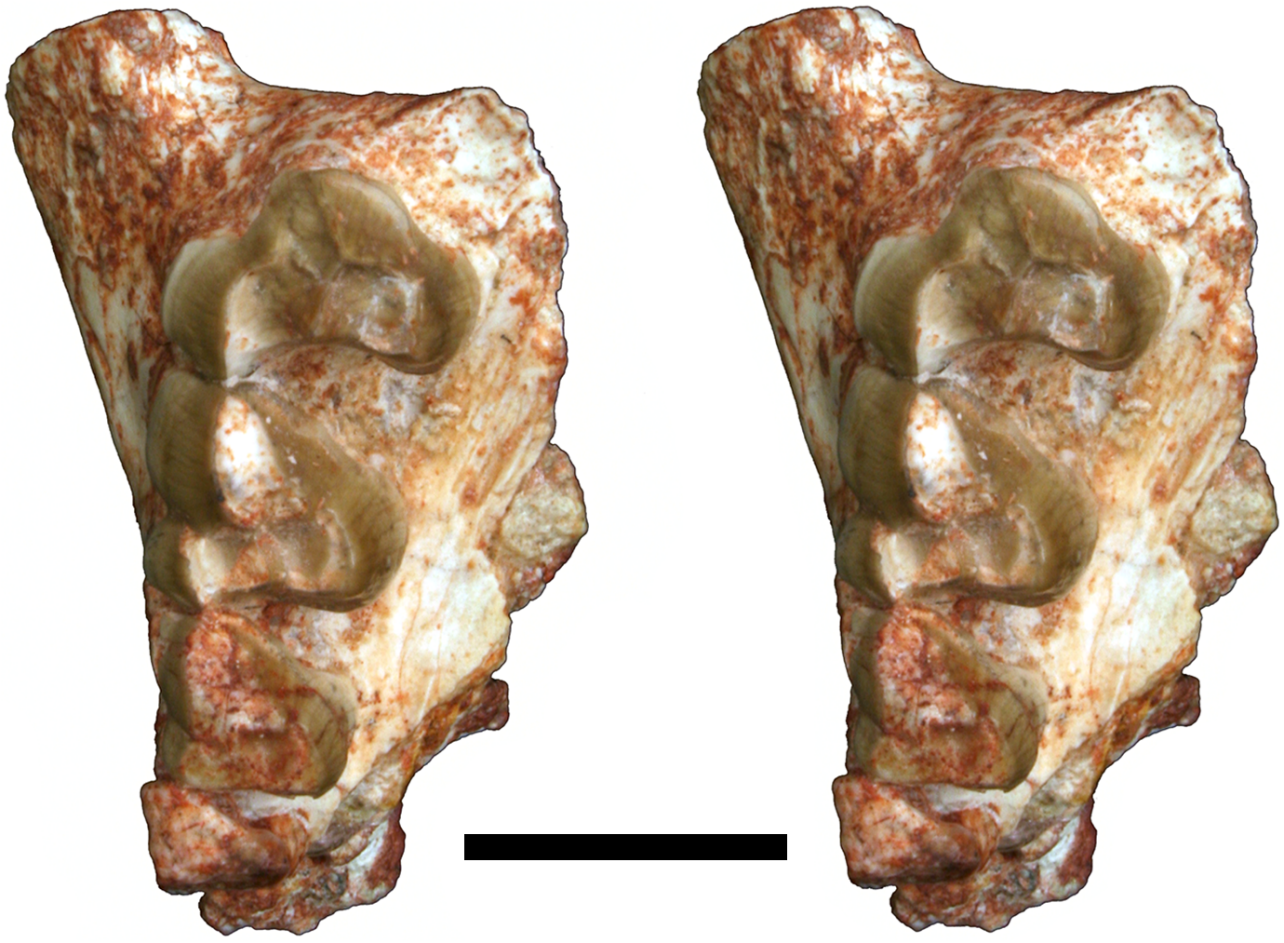
Stereo-photograph of left maxilla of holotype of *Anatoliadelphys maasae* (AÜJM 2002–25) in ventral (occlusal) view. M1-4 are preserved. Scale bar = 1 cm.

The left upper molars are somewhat better preserved than those on the right side (Figs 4 and 5). From anterior to posterior, molar size increases markedly (Table 1). M1-M3 are triangular in occlusal outline, whilst M4 has an enlarged parastylar region and greatly reduced metastylar region.

**Table 1:** Dimensions of upper teeth (in mm) of AÜJM 2002–25, holotype of *Anatoliadelphys maasae*.

Tooth	Measurement	Left side	Right side
C1	maximum mesiodistal length	-	6.9
	maximum labiolingual width	-	4.1
P1	maximum mesiodistal length	-	-
	maximum labiolingual width	-	-
P2	maximum mesiodistal length	-	4.1
	maximum labiolingual width	-	2.9
P3	maximum mesiodistal length	-	5.7
	maximum labiolingual width	-	5.1
M1	maximum mesiodistal length	-	4.6
	maximum labiolingual width	-	5.0
M2	maximum mesiodistal length	5.9	6.1
	maximum labiolingual width	6.4	6.8
M3	maximum mesiodistal length	8.1	7.6
	maximum labiolingual width	8.7	8.5
M4	maximum mesiodistal length	7.0	6.8
	maximum labiolingual width	10.2	9.7

M1 is the smallest molar, and is so heavily worn that no morphological interpretation can be made beyond that it is broadly triangular in outline, probably with an ectoflexus present.

M2 is less worn than M1, but its cusps are nevertheless barely identifiable due to the degree of wear. It appears broadly similar in occlusal outline to M1 but is ~30% larger in linear dimensions. A distinct ectoflexus is present. Two large stylar cusps appear to be present, one either side of the ectoflexus, with a possible third present at the posterolabial corner of the tooth; based on their positions and on comparison with other metatherians, we identify them (from anterior to posterior) as StB, StD and? StE, of which StD is the tallest, followed by StB. What remains of the metacone is taller than the paracone. The centrocrista is heavily worn, but appears to have been v-shaped. The preparacrista is much shorter than the postmetacrista. The protocone is anteroposteriorly broad, and is anterior to the midline of the tooth, in line with the paracone. A narrow cingulum extends along the anterior margin of the tooth, low down on the tooth crown, from level with the protocone to the anterolabial corner of the tooth; it may represent a conjoined precingulum and anterolabial cingulum. A similar, but somewhat narrower postcingulum extends along the posterior margin of the tooth, behind the postmetacrista, to the posterolabial corner of the tooth.

M3 is ~20% larger in linear dimensions than M2 and is the largest upper molar in terms of occlusal area. The ectoflexus is deeper than on M2. Anterior to the ectoflexus, there is a relatively well-developed StB. Posterior to the ectoflexus, the stylar shelf forms a labiolingually compressed crest that extends along the labial margin of the tooth; anteriorly, this crest rises to a distinct stylar cusp, which we identify as StD. This cusp is the tallest on the tooth crown, and is connected to the metacone via the premetacrista. The metacone is distinctly taller than the paracone.

The molar crests are better preserved on the right M3 than on the left. The preparacrista runs toward the anterolabial corner of the tooth, but terminates just before it. The preparacrista is slightly notched in anterior view. The centrocrista is unusual in that the postparacrista and premetacrista do not contact each other directly; instead, the premetacrista extends labially to terminate at StD, whilst the postparacrista extends posterolabially from the paracone and appears to terminate at the base of the premetacrista. A superficially similar centrocrista morphology is seen in *Hatcheritherium alpha* from the Late Cretaceous of North America [13], but in this taxon the premetacrista terminates at StC (which is absent in *Anatoliadelphys*), anterior to a very tall StD. The premetacrista is distinctly notched.

The postmetacrista is significantly longer than the premetacrista, and it rapidly reduces in height as it extends from the metacone towards the posterolabial corner of the tooth. The trigon basin is almost flat. The protocone is anterior to the midline of the tooth, and slightly anterior to the ectoflexus. The protocone is anteroposteriorly broad, and the pre- and postprotocrista meet at almost a right angle. Distinct conules are not present. Similarly to M2, a narrow cingulum extends along the anterior margin of the tooth, low down on the tooth crown, from level with the protocone to the anterolabial corner of the tooth; it broadens at the level of the paracone and remains the same width as it continues labially, where it may represent the homologue of the anterolabial cingulum. A narrow cingulum also extends along part of the posterior margin of the tooth, from level with the metacone to the posterolabial corner of the tooth.

The M4 is proportionately longer anteroposteriorly (relative to M1-3) than those of most other tribosphenic metatherians, due in part to its large parastylar region; however, it still appears somewhat anteroposteriorly compressed relative to the more anterior molars, due to reduction of the metastylar region. The most prominent cusp is the massive, spire-like paracone, which is connected to the metacone via a well-developed, linear centrocrista. As in the more anterior molars, a preparacrista extends towards the anterolabial corner of the tooth, but terminates just before reaching it. The postmetacrista is high, short and terminates at a small swelling that is probably StE. Anterior to this, a tiny bump on the labial margin of the tooth may be StD. Much larger stylar cusps are present in the B and C positions. In occlusal view, StB is anteroposteriorly broad and crestlike, dominating the parastylar region, but it is similar in height to StC when viewed labially. A crest extends lingually from the metacone towards the postprotocrista; a tiny swelling at its midpoint might represent the metaconule. The preprotocrista and postprotocrista meet at right angles. As in M2 and M3, the protocone is anteroposterorly broad and positioned anteriorly relative to the midline of the tooth, and a weakly developed cingulum extends along the entire anterior margin of the tooth, low down on the crown. A postcingulum also extends along the posterior margin; this cingulum is slightly wider than on M3, and extends labially from a point below the swelling that might represent the metaconule to stylar cusp E.

#### Maxilla

Fragments of both the left and right maxillae are present (Figs 2, 3 and 5). The largest and best preserved of these is a fragment of the right maxilla that retains the C1, P1-3 and M1-3 (Figs 2 and 3; these teeth are described above). When this fragment is viewed laterally, the infraorbital foramen can be identified as an opening in a relatively posterior position, above the anterior root of M2. The infraorbital foramen appears relatively well-developed, but the incompleteness of the cranium means that its relative size cannot be easily compared to the infraorbital foramina of other metatherians [75, 102].

The medial side of this maxillary fragment is concealed by matrix, but CT scans reveal the presence of a piece of the right nasal within the matrix. The CT scans also suggest that some of the maxillary turbinals and the maxillary foramen (the posterior opening of the infraorbital canal) are also preserved. In ventral view, only the alveolar process and a small portion of the palatal process are preserved; the lateral edge of a palatal vacuity may be present medial to P3-M1, but the specimen is too damaged to be certain of this. Palatal vacuities are found in most metatherians, but are absent in deltatheroidans, sparassodonts, *Pucadelphys* and *Mayulestes*, all of which fall outside Marsupialia [103].

Other cranial fragments include one comprising a part of the palatal process of the maxilla with the right M4 *in situ*, together with part of the palatine (this fragment has been glued to the larger right maxillary fragment described above–see Fig 2), and another comprising a partial left maxilla with M1-4 *in situ* (Fig 5). A notable feature of these fragments is the presence of a deep pit in the palatal process of the maxilla, between M3 and M4, which would have housed the very large protoconid of m4 during occlusion. Similar pits have been reported in sparassocynids [104], sparassodonts (character 21 of [32]) and *Thylacinus* species [105].

#### Basicranial fragment

**Fig 6:**
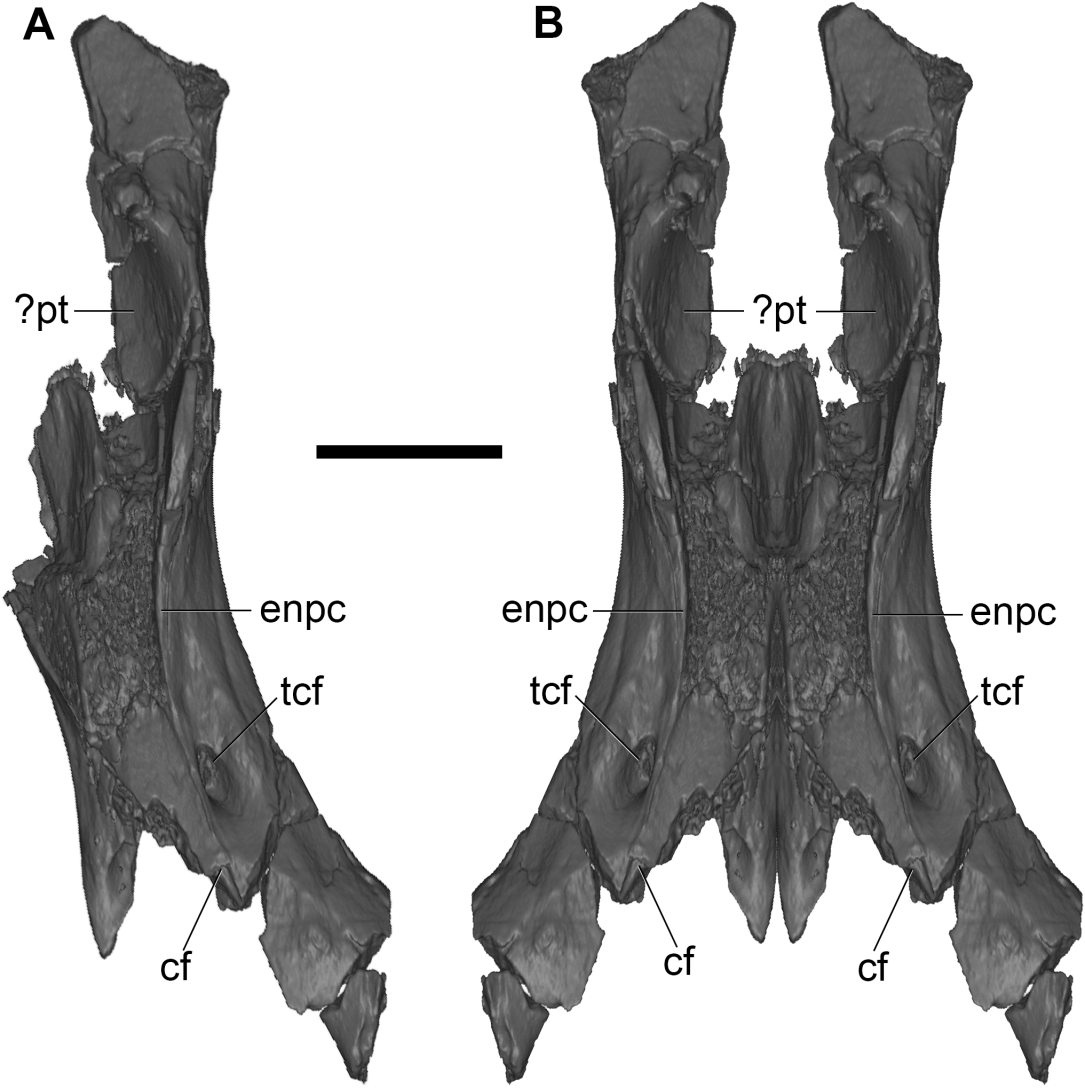
Volume-rendered CT images of nasopharyngeal-sphenoid region of holotype of *Anatoliadelphys maasae* (AÜJM 2002–25) in ventral view. **A**, cranial fragment from left side; **B**, mirrored version of A, showing approximate morphology of intact nasopharyngeal-sphenoid region. Abbreviations: cf, carotid foramen; enpc, entopterygoid crest;? pt,? pterygoid; tcf, transverse canal foramen. Scale bar = 1 cm.

A long, badly damaged fragment can be identified as part of the left side of the basicranium, preserving parts of the palatine, basisphenoid and alisphenoid, and possibly also parts of the pterygoid and presphenoid (Fig 6). In ventral view, the most easily identifiable feature is the transverse canal foramen, which is found in most (but not all) metatherians [106], but which is less common in eutherians. Posteriorly, there is an opening that extends anterodorsally, which we tentatively identify as the carotid foramen; if so, this foramen may be located entirely within the basisphenoid, but this cannot be confirmed because of poor preservation. Nevertheless, it is clear that the transverse canal foramen is positioned anterior to the carotid foramen, as in most metatherians [37, 106]. The crest medial to the transverse canal foramen is probably the posterior part of the entopterygoid crest, which gradually enlarges anteriorly and joins the lateral wall of nasopharyngeal passage.

In dorsal view, the crests forming the border of the sulcus for the internal opening of the foramen rotundum can be identified. The hypophyseal fossa is badly damaged. A small portion of the lateral wall of alisphenoid is preserved above the sulcus for the foramen rotundum. The crest anteromedial to the internal opening of the foramen rotundum is possibly the lateral border of the sphenorbital fissure. On the left side of the anteriormost edge of this basicranial fragment, a sulcus that may be for the sphenopalatine emissary vein is preserved, as is the posterior border of the sphenopalatine foramen.

#### Jugal

**Fig 7:**
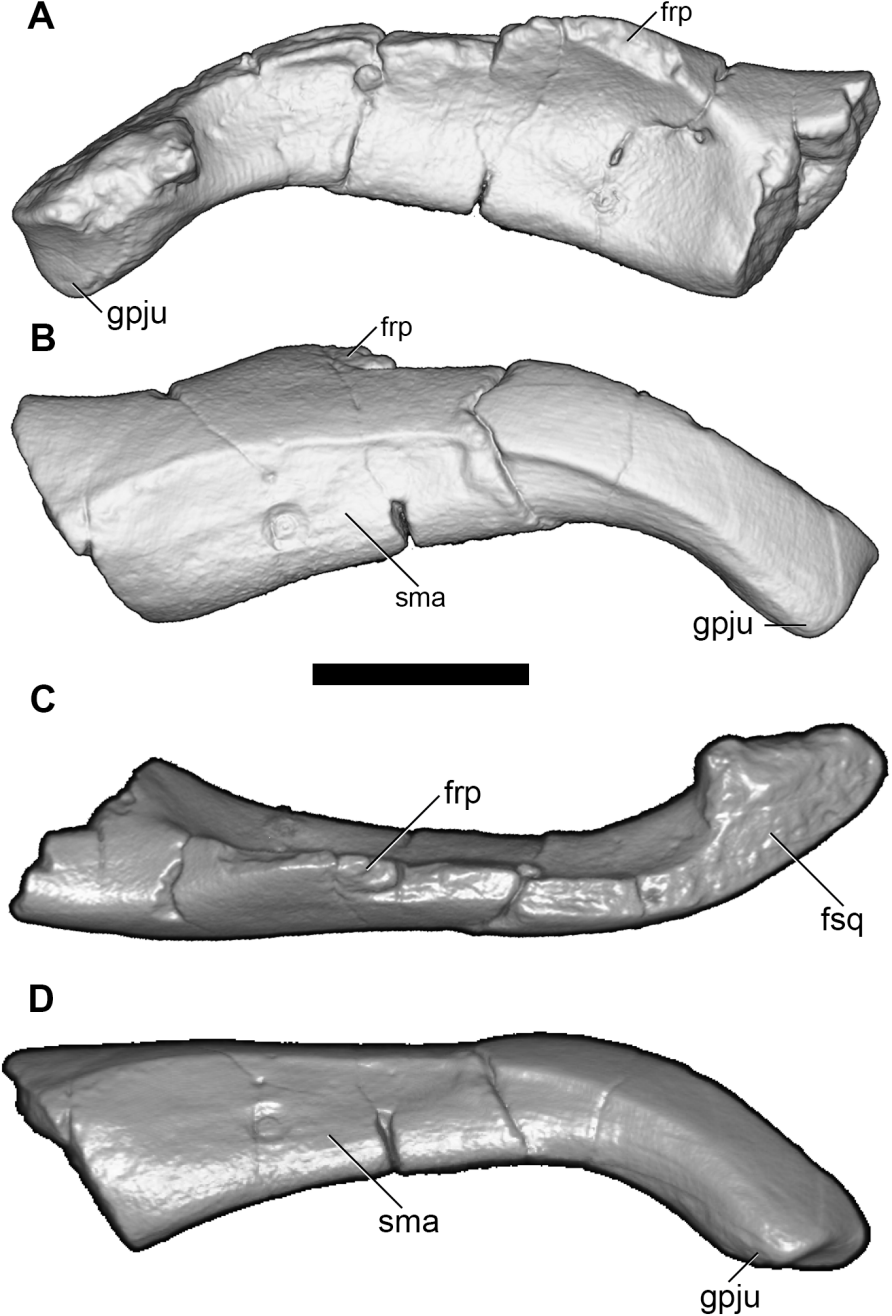
Volume-rendered CT images of left jugal of holotype of *Anatoliadelphys maasae* (AÜJM 2002–25). **A**, medial view; **B**, lateral view; **C**, dorsal view; **D**, ventral view. Abbreviations: frp, frontal process of the jugal; fsq, facet for squamosal; gpju, glenoid process of the jugal; sma, sulcus for the masseter muscle. Scale bar = 1 cm.

**Fig 8:**
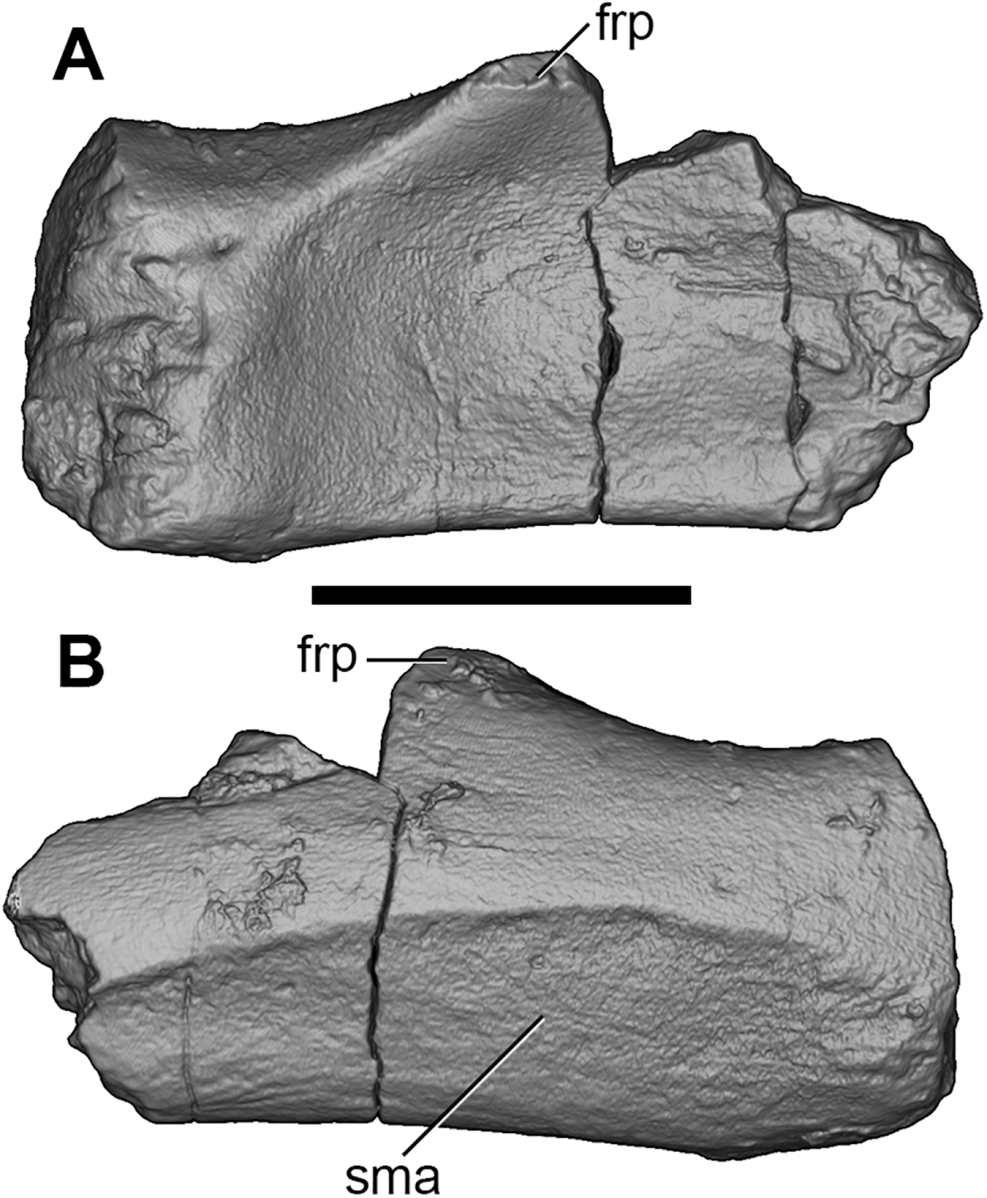
Volume-rendered CT images of right jugal of holotype of *Anatoliadelphys maasae* (AÜJM 2002–25). **A**, medial view; **B**, lateral view. Abbreviations: frp, frontal process of the jugal; sma, sulcus for masseter muscle. Scale bar = 1 cm.

Parts of both the left and right jugals are preserved (Figs 7 and 8). The left fragment is almost complete, missing only a small portion of its posterodorsal margin (Fig 7); it articulates perfectly with the preserved zygomatic process of the left maxilla. The right fragment is more damaged, and only the middle third is preserved (Fig 8).

Anteriorly, there does not appear to be a distinct depression on the lateral wall of the zygomatic process of the jugal for the zygomaticus and levator labii muscles, whereas such a depression is seen in at least some didelphids [36, 107]; however, this region is slightly damaged in AÜJM 2002–25. A distinct frontal process (representing the ventral point of attachment of the postorbital ligament) is identifiable on the dorsal margin of the left jugal fragment, but it is damaged. In ventral view, the jugal widens posteriorly, and forms a distinct, faceted preglenoid process, which would have formed the anterolateral margin of the glenoid fossa [36]. The medial surface of the jugal is concave, and it houses a large nutrient foramen anterior to the frontal process.

#### Squamosal

**Fig 9:**
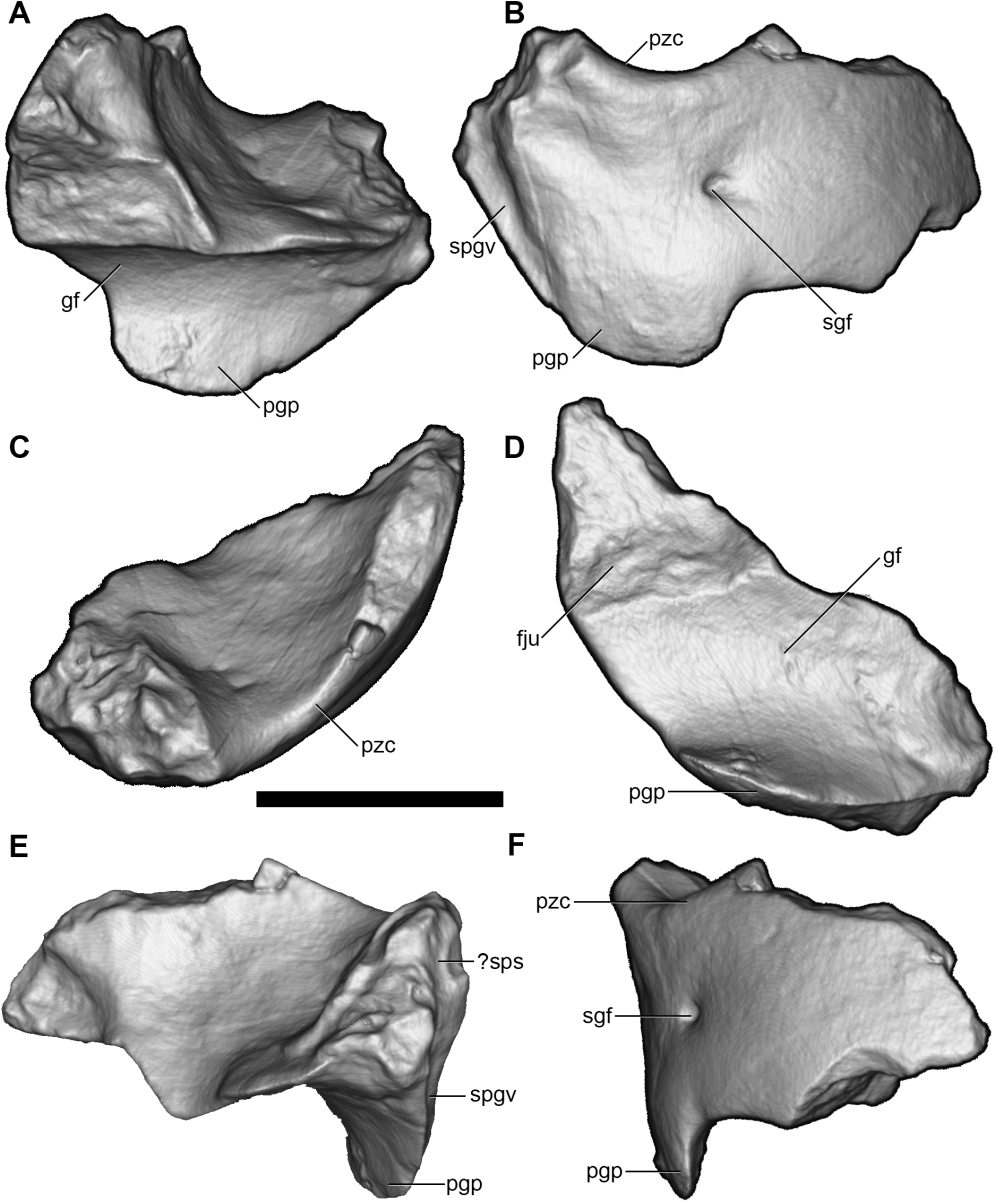
Volume-rendered CT images of partial right squamosal of holotype of *Anatoliadelphys maasae* (AÜJM 2002–25). **A**, anterior view; **B**, posterior view; **C**, dorsal view; **D**, ventral view; **E**, medial view; **F**, lateral view. Abbreviations: fju, facet for jugal; gf, glenoid fossa; pgp, postglenoid process; pzc, postzygomatic crest; sgf, supraglenoid foramen; spgv, sulcus for postglenoid vein;? sps,? sulcus for prootic sinus. Scale bar = 1 cm.

**Fig 10:**
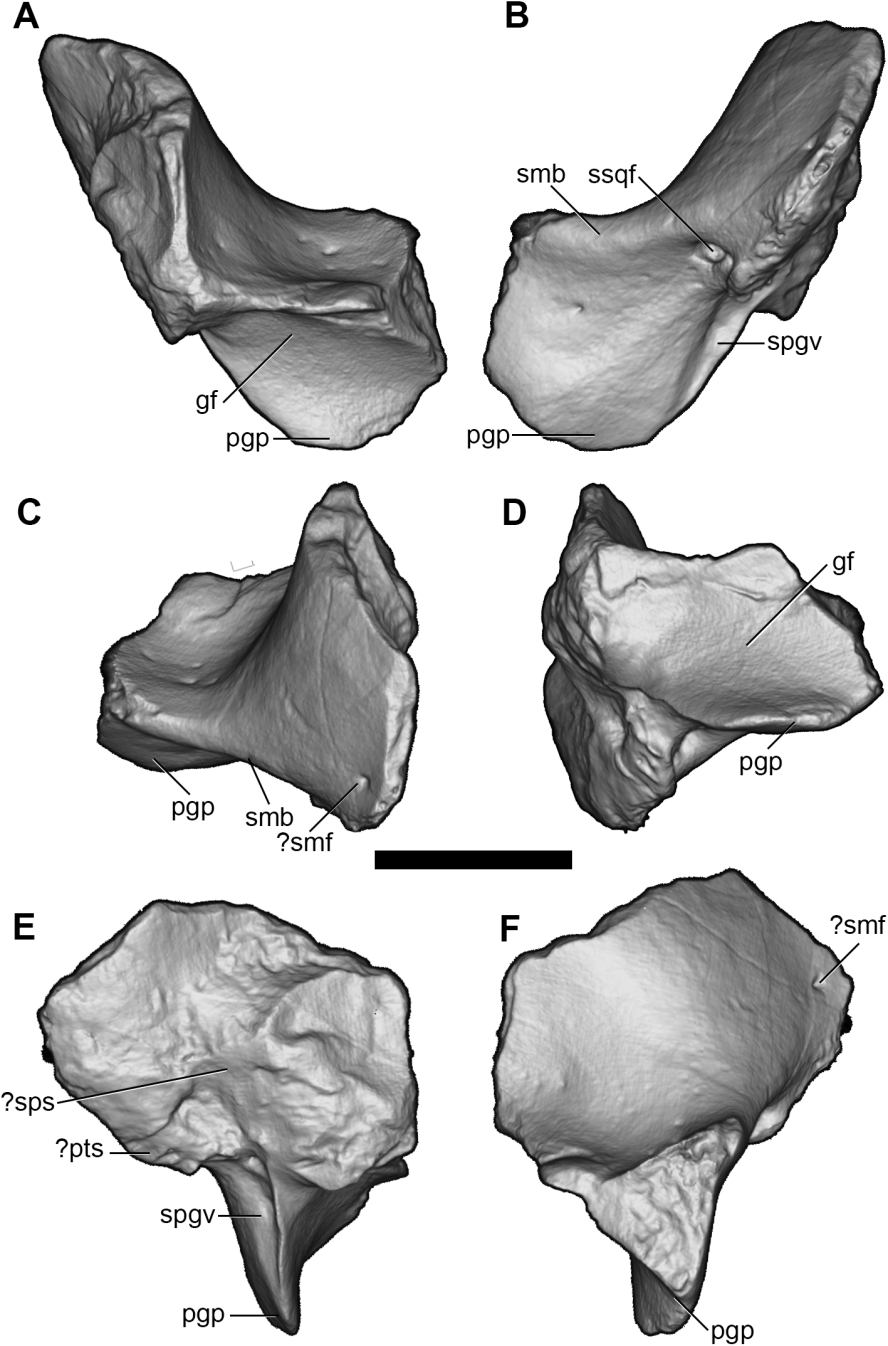
Volume-rendered CT images of partial left squamosal of holotype of *Anatoliadelphys maasae* (AÜJM 2002–25). **A**, anterior view; **B**, posterior view; **C**, posterodorsal view; **D**, ventral view; **E**, medial view; **F**, lateral view. Abbreviations: gf, glenoid fossa; pgp, postglenoid process;? pts,? posttemporal sulcus; sgf, supraglenoid foramen; smb, suprameatal bridge;? smf,? suprameatal foramen (= “subsquamosal foramen” of Wible [36]); spgv, sulcus for postglenoid vein;? sps,? sulcus for prootic sinus; ssqf, subsquamosal foramen (= “suprameatal foramen” of Wible [36]). Scale bar = 1 cm.

Parts of both the right and left squamosals are preserved (Figs 9 and 10). Only the glenoid fossa and the postglenoid process of the right squamosal are preserved (Fig 9), but the left squamosal is nearly complete, lacking only the zygomatic process (Fig 10).

In lateral view, the squamosal appears roughly rectangular in shape. The suprameatal bridge is short but robust. The subsquamosal foramen [27, 31, 108–110] (= the “suprameatal foramen” of Wible [36]), which transmits a temporal branch of the postglenoid artery and vein, is paired, with the anterior opening much larger than the posterior. Dorsal to the suprameatal bridge, there is at least one vascular opening that probably represents a subsquamosal foramen (= “suprameatal foramen” *sensu* Wible [36]). There is also a distinct supraglenoid foramen opening in the lateral side of the zygomatic process of the squamosal, dorsal to the postglenoid process. In ventral view, the glenoid fossa is mediolaterally elongate and roughly oval in shape. The anterolateral margin of the glenoid fossa would have been bordered by a prominent glenoid process of the jugal in life (see above). The postglenoid process appears robust and unpneumatised. The medial edge of the postglenoid process is grooved by a prominent sulcus that presumably transmitted the postglenoid vein from the postglenoid foramen, but the postglenoid foramen itself is not preserved intact; it may have been completed medially by the petrosal, as is seen in some marsupials (e.g. some peramelemorphians [111]). In medial view, the vertical sulcus for the prootic sinus may be faintly visible on the squamosal, and on the left squamosal a horizontal sulcus may represent the posttemporal sulcus housing the arteria and vena diploetica magna [35].

#### Posterior cranial roof

**Fig 11:**
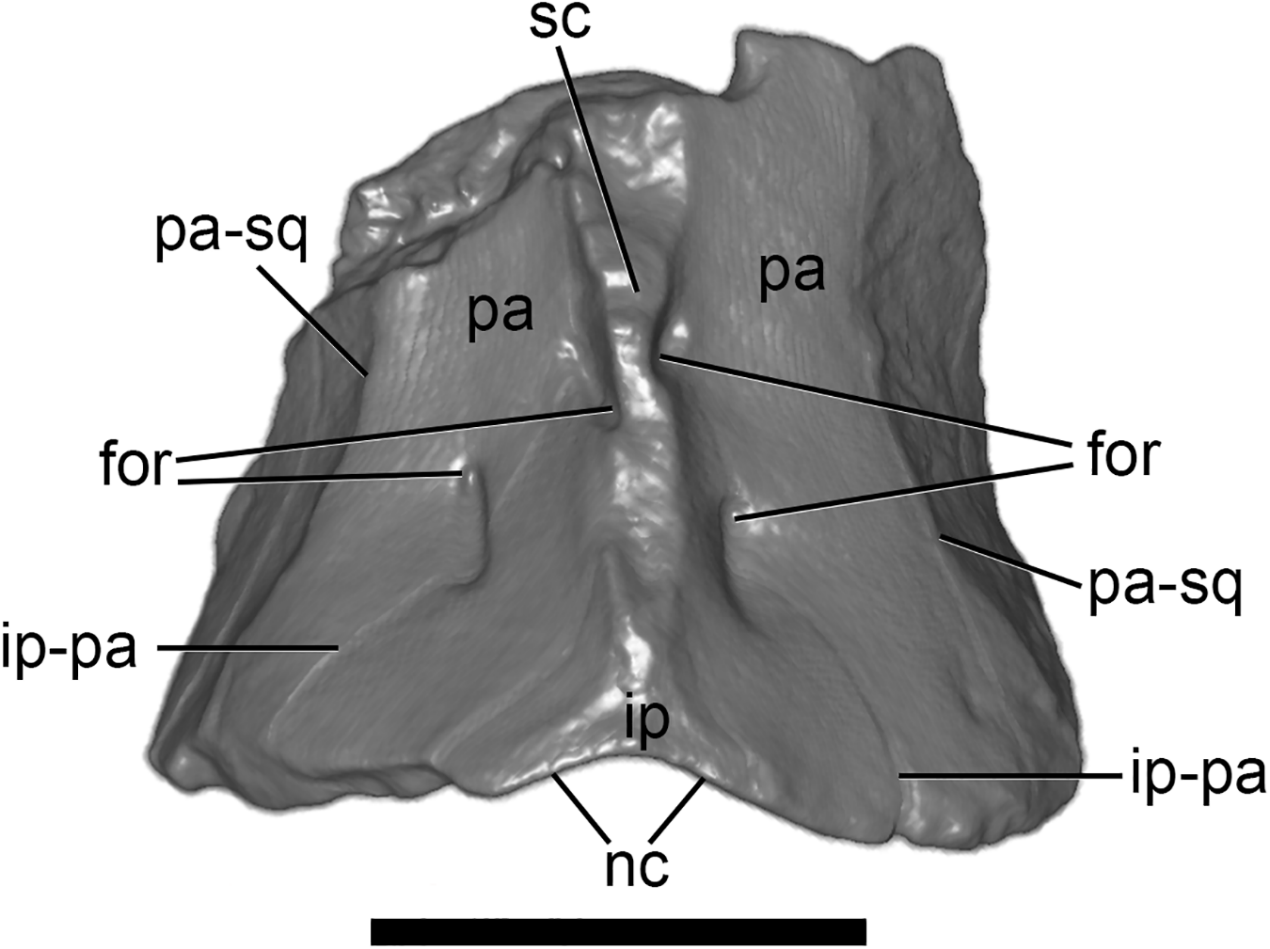
Volume-rendered CT image of cranial roof of holotype of *Anatoliadelphys maasae* (AÜJM 2002–25) in dorsal view. Only the posterior part of the dorsal cranial roof is preserved. Abbreviations: for, foramina; ip, interparietal; ip-pa, interparietal-parietal suture; nc, nuchal (lambdoid) crest; pa, parietal; pa-sq, path of parietal-squamosal suture; sc, sagittal crest. Scale bar = 1 cm.

A fragment representing the posterior part of the dorsal cranial roof is preserved (Fig 11); it appears to comprise the interparietal (posterodorsally), the paired parietals (dorsolaterally), and supraoccipital (posteriorly). In dorsal view, a thick, well-developed sagittal crest is clearly identifiable, extending anteriorly from the nuchal (lambdoid) crest, along the interparietal and onto the parietals; the crest is damaged anteriorly. Multiple pairs of foramina are present on the dorsal surface of this fragment, of which two bilateral pairs are particularly prominent. One pair of these is located approximately halfway along the preserved length of the fragment, either side of the base of the sagittal crest, at the anterior end of the interparietal; these foramina penetrate anteroventrally into the cranium. The second pair is positioned more posterolaterally, opening adjacent to or within the interparietal-parietal suture; a posteriorly-directed sulcus extends from each of these foramina. These foramina may be for emissary veins draining venous blood. Further laterally, a raised ridge marks the suture with the squamosal.

Didelphids are unusual among mammals in that the supraoccipital and interparietal are entirely fused without any trace of a suture in postweaning juveniles or older individuals [27, 36]; in other marsupials, by contrast, the suture between supraoccipital and interparietal can usually be identified, roughly corresponding to the path of the nuchal crest (if present), except in very old or heavily ossified individuals [27]. Based on its known morphology and the results of our phylogenetic analysis (see below), *Anataoliadelphys maasae* is clearly not a didelphid; nevertheless, a suture delimiting the boundary between interparietal and supraoccipital cannot be clearly identified in AÜJM 2002–25. We are confident that an interparietal was present as a distinct bone, as it appears to be consistently present in mammals [112]. However, the interparietal-supraoccipital suture may be concealed by the prominent nuchal crest of this specimen. Similarly, the midline suture is not apparent in AÜJM 2002–25, but might be concealed by the very prominent sagittal crest; the midline parietal suture is fused in some marsupials [27].

In posterior view, the nuchal crest appears well-developed, with a concave posterior surface; however, the ventral half of the posterior surface is somewhat crushed and displaced anteriorly, exaggerating the concavity of the surface. Multiple bilaterally symmetric foramina are present on the posterior surface. Posteroventral to the point of contact between the nuchal and sagittal crests, there is a median foramen. In anterior view, the vermis impression appears round and deep.

#### Other cranial fragments

As discussed above, CT scanning of the large right maxillary fragment reveals that part of the right nasal is preserved within the matrix that covers the medial side. However, this nasal fragment does not preserve any remarkable features.

A large fragment of bone was found attached to the angular process of the left mandible, which had broken off the main body of the mandible. Due to their fragile nature, it was not possible to separate these fragments and reattach the mandibular parts to the rest of the mandible. The non-mandibular fragment has a slightly convex outer surface, and so could represent either part of the iliac blade or a part of the sidewall of the cranium. However, both ilia are almost complete, and the weathering pattern and pigmentation of this fragment closely resembles the right squamosal. We therefore interpret it as a fragment of the right cranial wall. The lateral side is mostly convex, with a flaring posterodorsal margin. The anterodorsal margin bears a small nutrient foramen with a posterodorsal opening. Most of the medial view is obscured by the overlying angular process of the left mandible.

Two petrosal fragments are preserved, but both are heavily damaged. Only an elongate mastoid exposure and a conical and relatively shallow subarcuate fossa can be recognised on a fragment of the pars canalicularis of the right petrosal. The subarcuate fossa is relatively deep in most metatherians, but it is shallow in *Sarcophilus* and some sparassodonts (e.g. *Pharsophorus*), and it is reportedly entirely absent in the vombatids *Vombatus* and *Lasiorhinus* and some sparassodonts (e.g. *Lycopsis*, *Prothylacinus*, *Arctodictis*) [31, 32, 113].

#### Lower dentition

The left and right dentaries of AÜJM 2002–25 both preserve c1 and the entire postcanine dentition (p1-3 m1-m4), but these teeth are better preserved on the left side (Figs 12–19).

**Fig 12:**
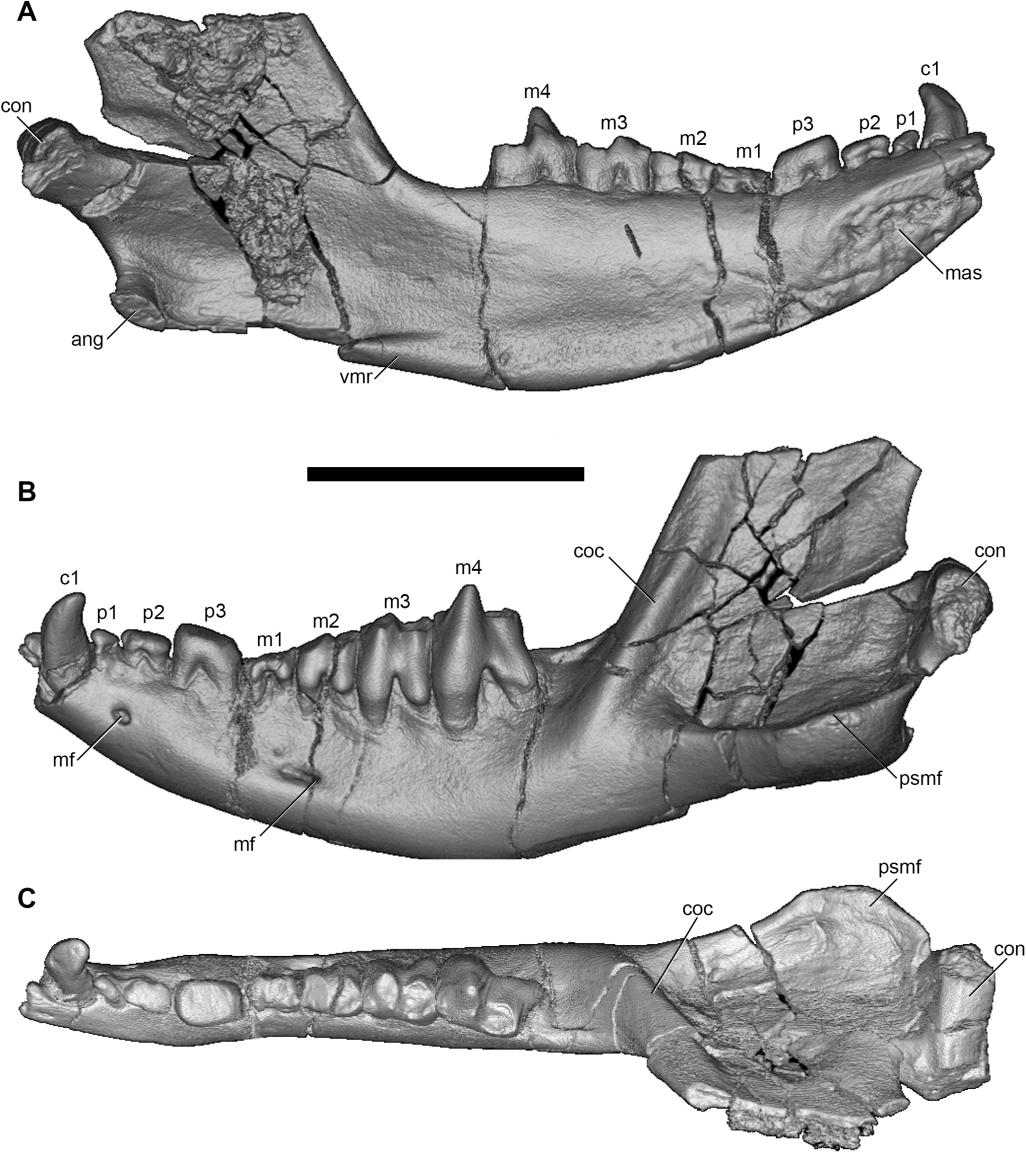
Volume-rendered CT image of left dentary of holotype of *Anatoliadelphys maasae* (AÜJM 2002–25). **A**, medial view; **B**, lateral view; **C**, dorsal (occlusal) view. Abbreviations: ang, angular process; coc, coronoid crest; con, dentary condyle; mas, mandibular symphysis; mf, mental foramen; psmf, posterior shelf of the masseteric fossa; vmr, ventromedial ridge. Scale bar = 2.5 cm.

**Fig 13:**
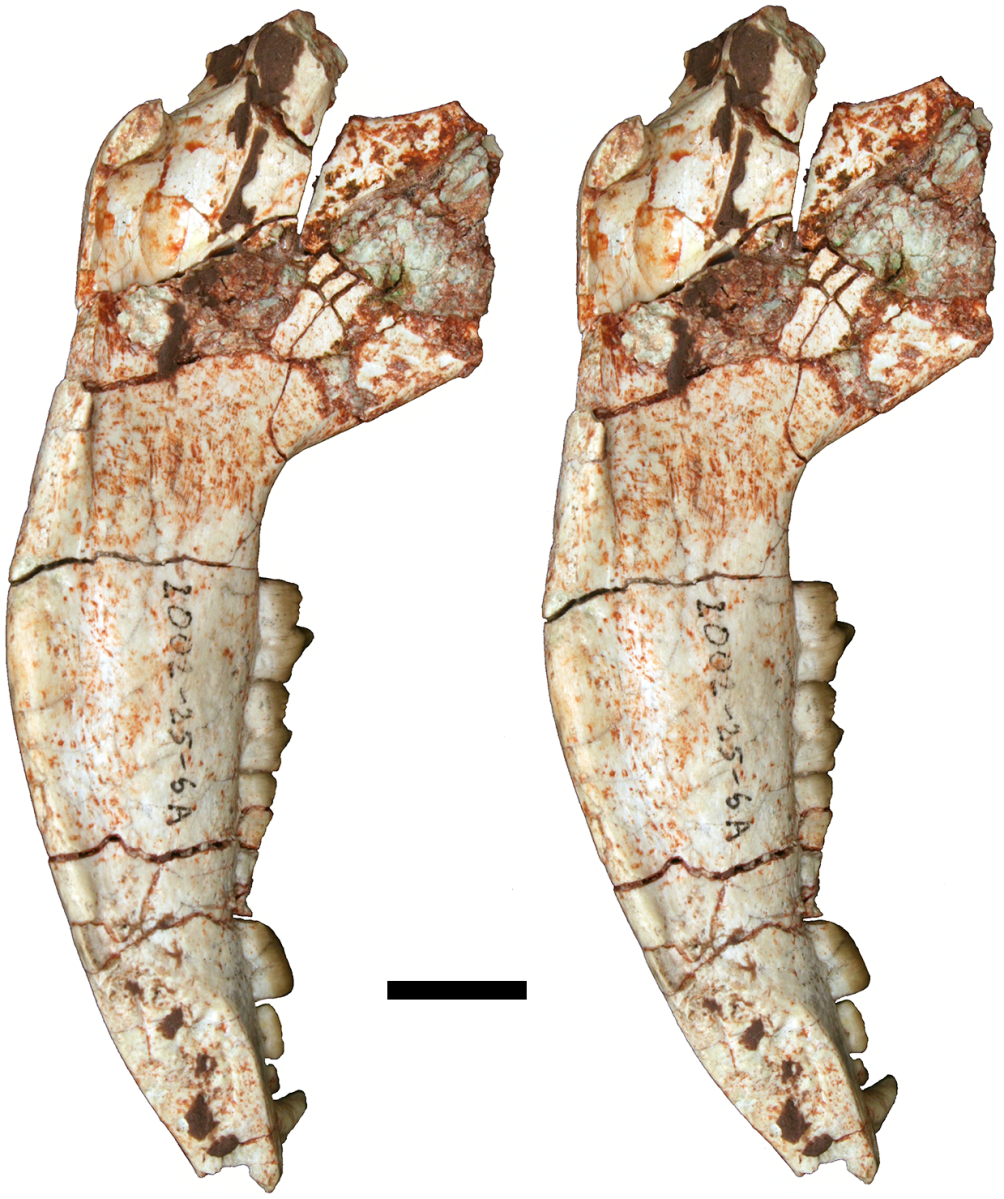
Stereo-photograph of left dentary of holotype of *Anatoliadelphys maasae* (AÜJM 2002–25) in medial view. Scale bar = 1 cm.

**Fig 14:**
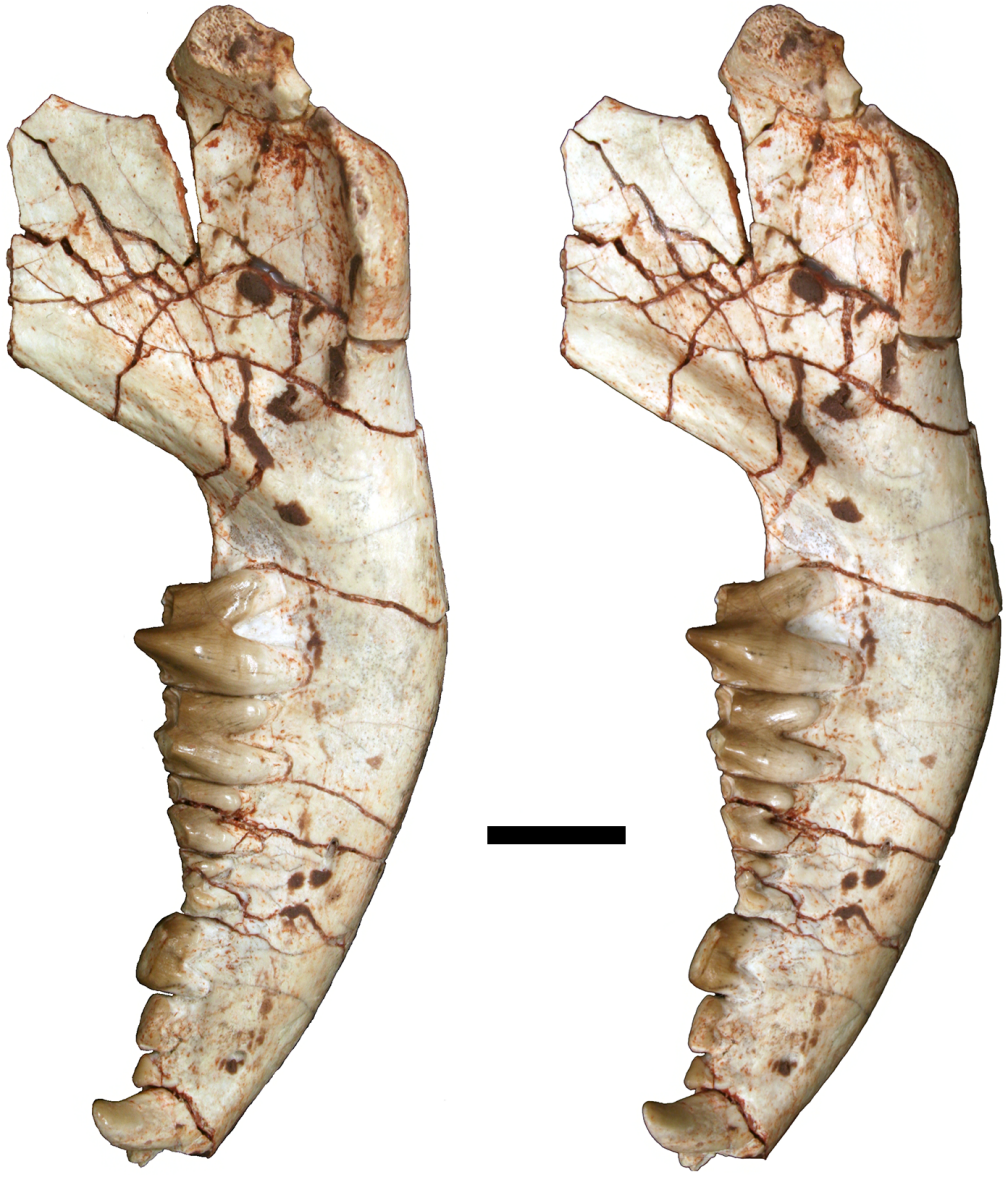
Stereo-photograph of left dentary of holotype of *Anatoliadelphys* maasae (AÜJM 2002–25) in lateral view. Scale bar = 1 cm.

**Fig 15:**
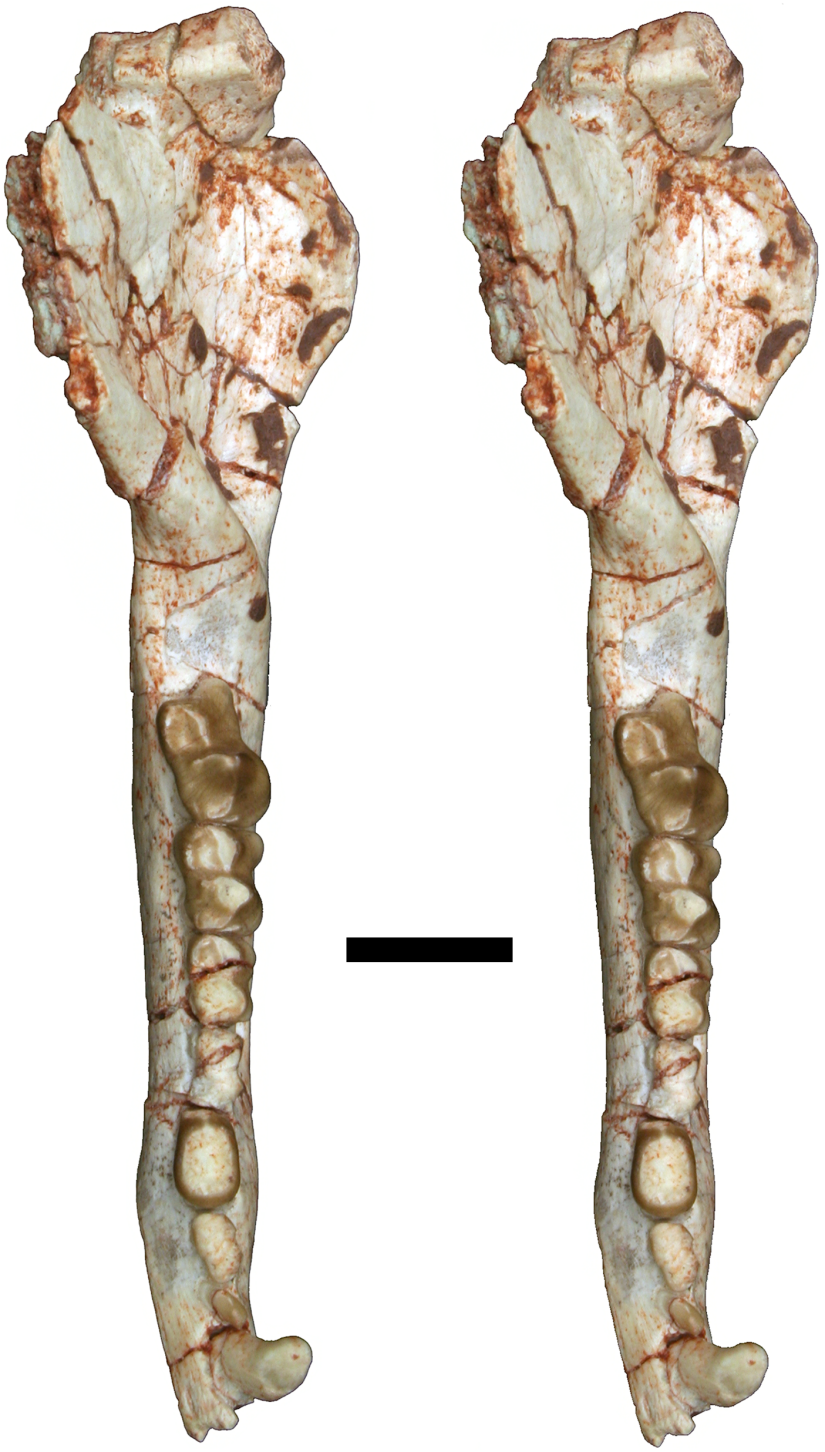
Stereo-photograph of left dentary of holotype of *Anatoliadelphys maasae* (AÜJM 2002–25) in dorsal (occlusal) view. Scale bar = 1 cm.

**Fig 16:**
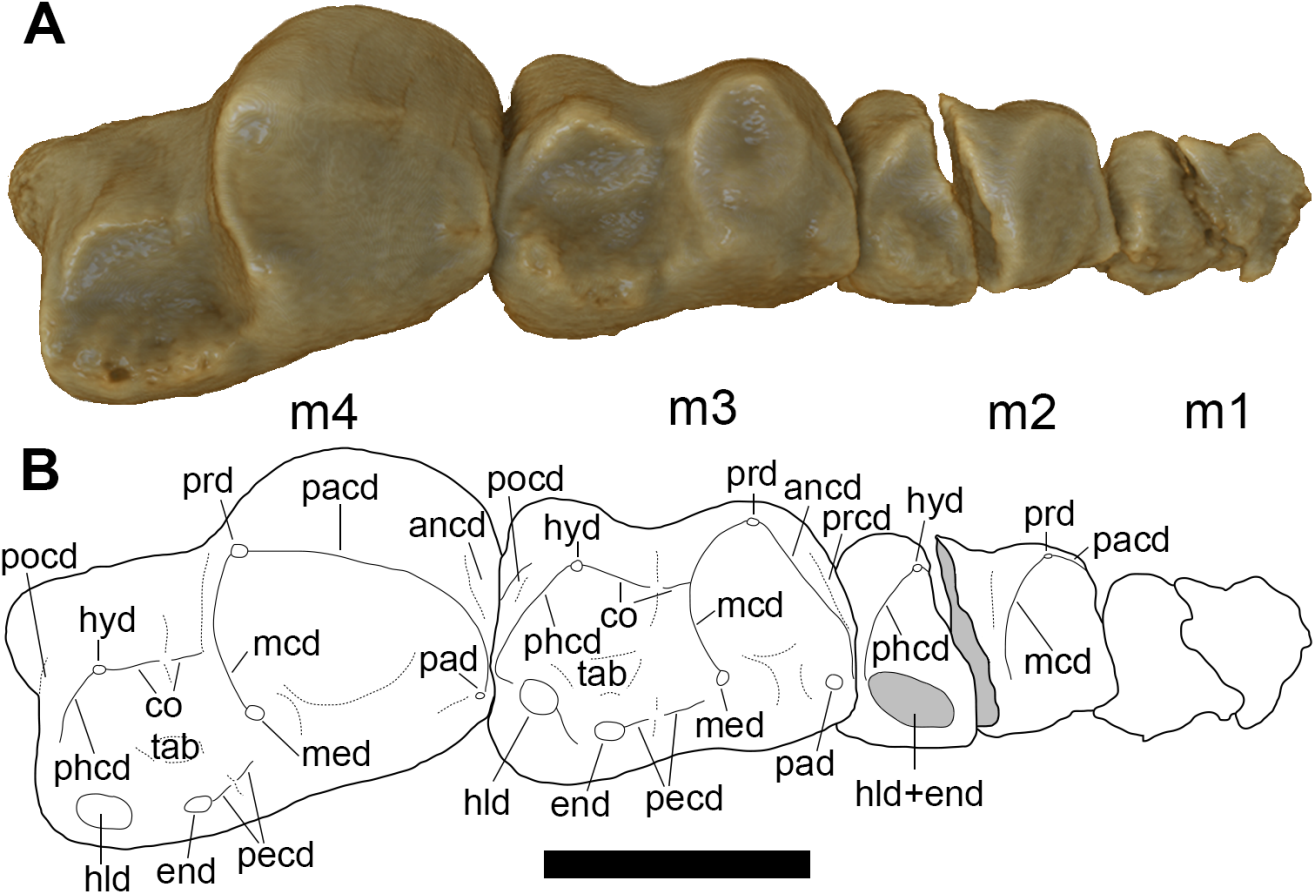
Left lower molars (m1-4) of holotype of *Anatoliadelphys maasae* (AÜJM 2002–25) in dorsal (occlusal) view. **A**, Volume-rendered CT image; **B**, interpretative drawing (damaged areas are indicated in grey). Abbreviations: ancd, anterior cingulid; co, cristid obliqua; end, entoconid; hld, hypoconulid; hyd, hypoconid; mcd, metacristid; med, metaconid; pacd, paracristid; pad, paraconid; pecd, preentocristid; phcd, posthypocristid; prd, protoconid; tab, talonid basin;. Scale bar = 0.5 cm.

**Fig 17:**
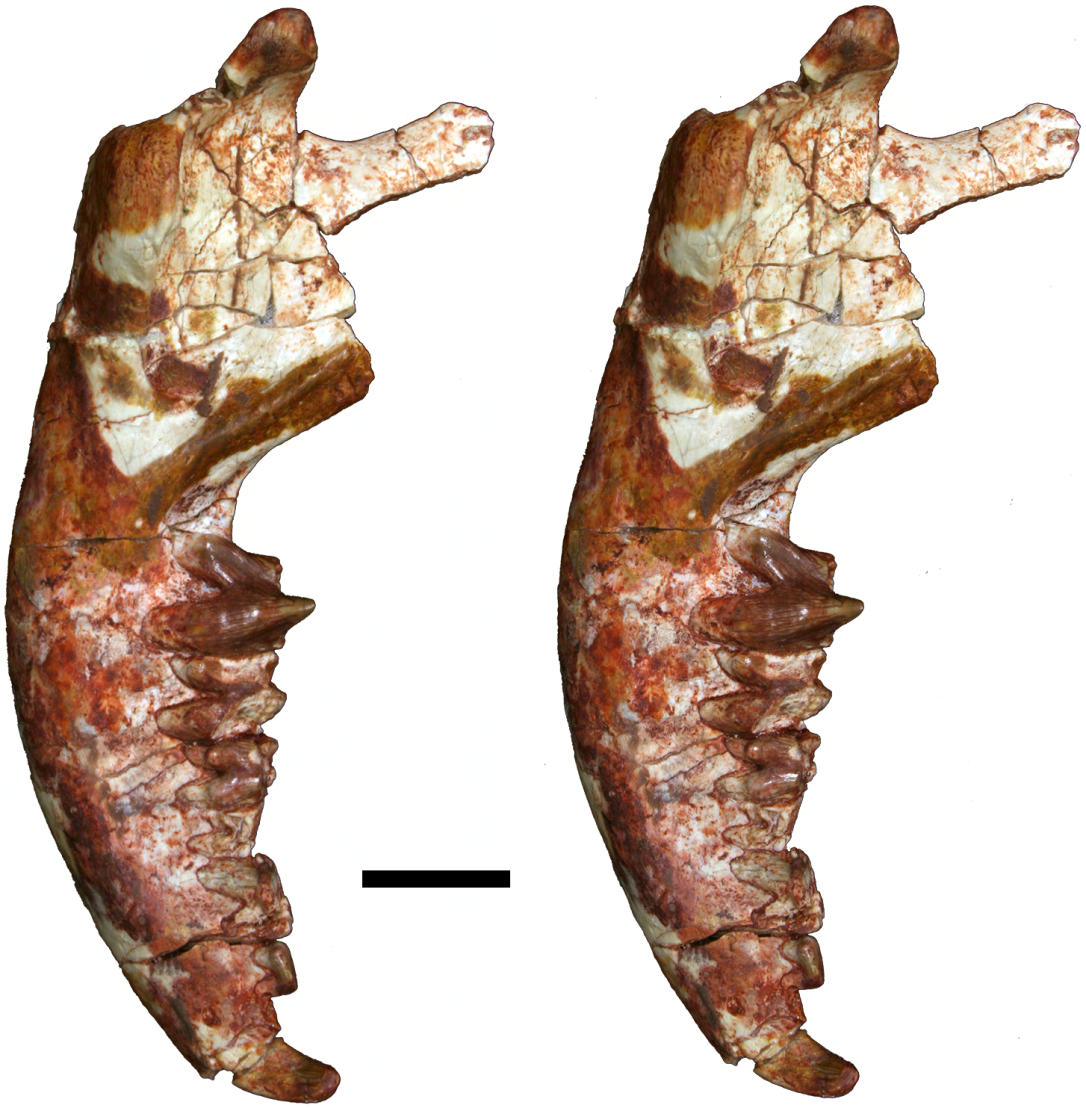
Stereo-photograph of right dentary of holotype of *Anatoliadelphys maasae* (AÜJM 2002–25) in medial view. Scale bar = 1 cm.

**Fig 18:**
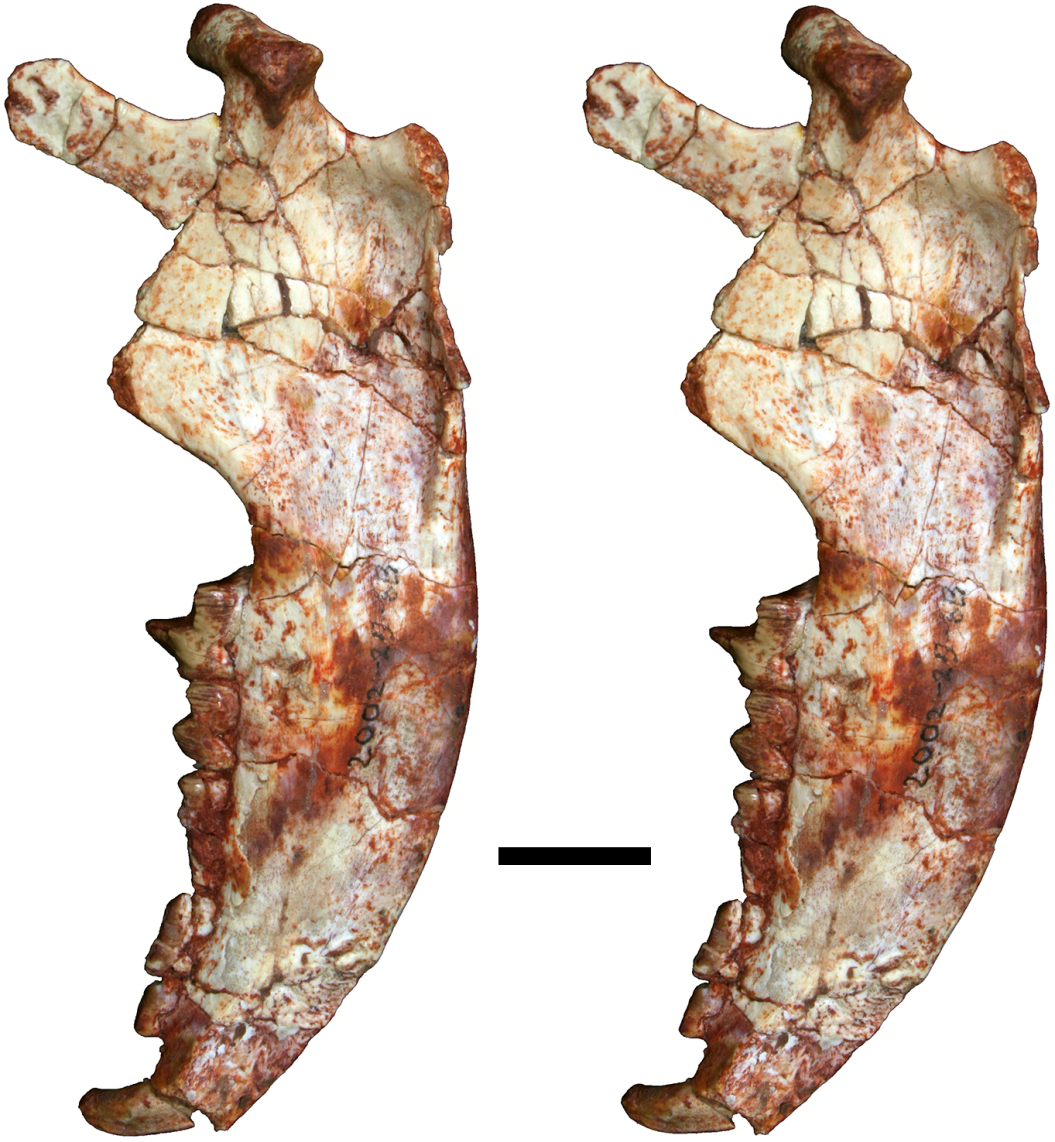
Stereo-photograph of right dentary of holotype of *Anatoliadelphys maasae* (AÜJM 2002–25) in lateral view. Scale bar = 1 cm.

**Fig 19:**
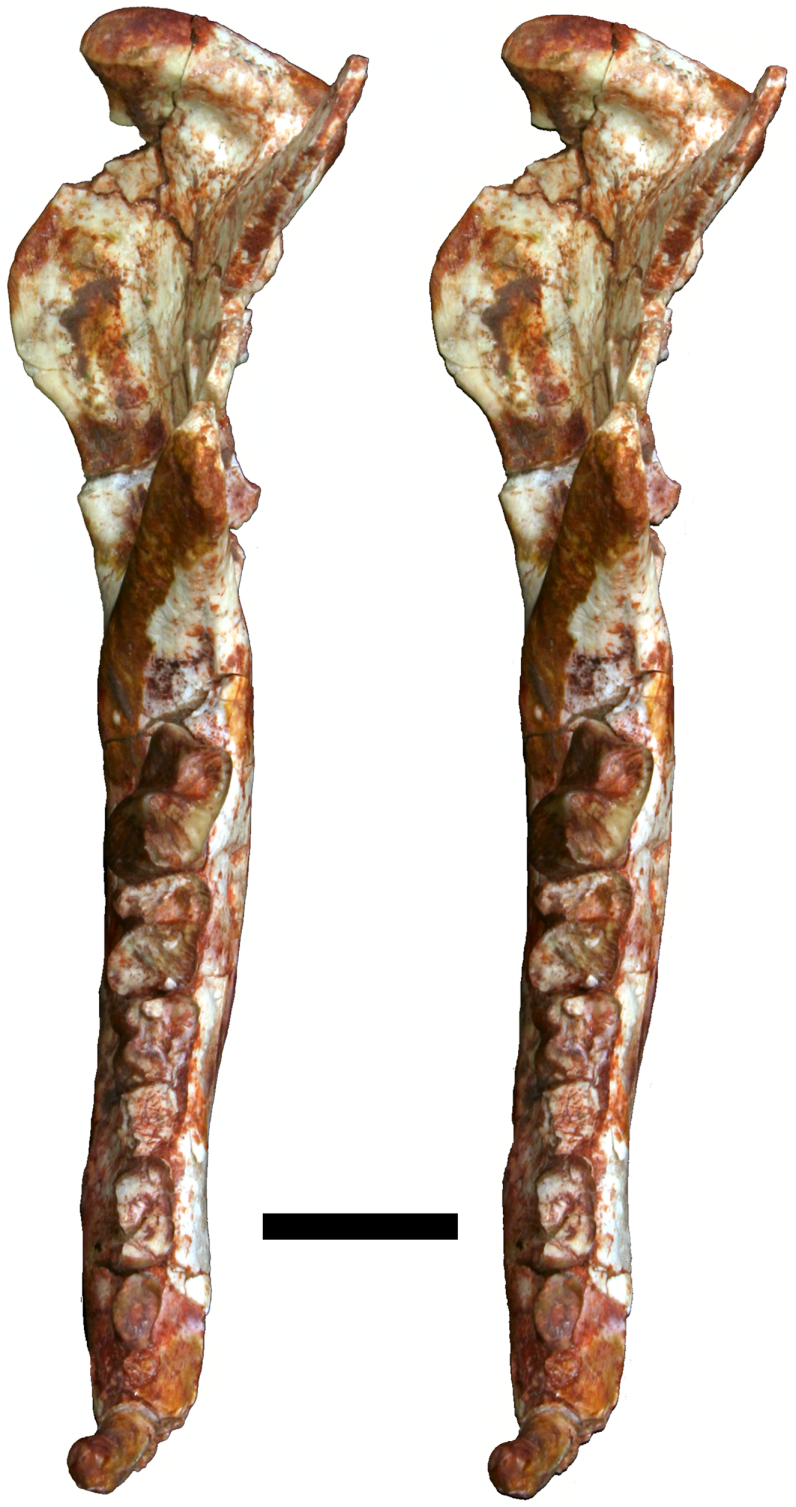
Stereo-photograph of right dentary of holotype of *Anatoliadelphys maasae* (AÜJM 2002–25) in dorsal (occlusal) view. Scale bar = 1 cm.

The c1 is a single rooted, caniniform tooth that is not as tall as C1.

Three premolars are present, namely p1-3. Of these, p1 is tiny but double-rooted, and is oriented posterolingually-anterolabially relative to the major axis of the dentary; it is heavily worn. The p2 is also double-rooted, markedly larger than p1 but also heavily worn. The double-rooted p3 is by far the largest of the three premolars; it is somewhat less worn than p1 and p2 (see our earlier comments about how this pattern of wear may indicate marsupial-type dental replacement), but most of the occlusal surface has nevertheless been lost through wear. It is broad labiolingually (width:length ratio = 0.70), and it is about as large as m2 in terms of occlusal area.

The lower molars increase markedly in size from anterior to posterior, with m4 being the largest tooth, with an occlusal area about seven times larger than that of m1 (Table 2). The lower molars are distinctly “exodaenodont” *sensu* Sigé *et al*. [114]: the labial side of the molar crown extends much further ventrally than does the lingual side of the crown, with the degree of ventral extension greater in m3-4 than in m1-2. The molar roots are also partially exposed in labial (but not lingual) view.

**Table 2:** Dimensions of lower teeth (in mm) of AÜJM 2002–25, holotype of *Anatoliadelphys maasae*.

Tooth	Measurement	Left side	Right side
c1	maximum mesiodistal length	4.0	4.4
	maximum labiolingual width	3.1	3.0
p1	maximum mesiodistal length	2.8	-
	maximum labiolingual width	1.5	-
p2	maximum mesiodistal length	4.4	4.7
	maximum labiolingual width	3.1	3.2
p3	maximum mesiodistal length	6.3	7.5
	maximum labiolingual width	4.4	-
m1	maximum mesiodistal length	5.2	4.9
	maximum labiolingual width of trigonid	3.3	-
	maximum labiolingual width of talonid	3.6	-
m2	maximum mesiodistal length	5.7	6.2
	maximum labiolingual width of trigonid	4.4	4.7
	maximum labiolingual width of talonid	5.0	5.5
m3	maximum mesiodistal length	7.0	7.5
	maximum labiolingual width of trigonid	5.7	6.2
	maximum labiolingual width of talonid	6.8	6.4
m4	maximum mesiodistal length	9.8	9.8
	maximum labiolingual width of trigonid	6.9	6.5
	maximum labiolingual width of talonid	5.7	5.7

In labial and lingual views, an unusual, more-or-less continuous wear surface can be seen on the left lower dentition, formed by the occlusal surfaces of m1, m2 and the trigonid of m3; this wear surface is not as obvious on the right lower dentition because of damage to the labial side of m3.

The m1 and m2 are both heavily worn, and so their occlusal morphology cannot be identified. On m3, the paraconid and metaconid are both low. The protoconid is worn to its base, and so it is not possible to confidently determine its original height; however, based on its large base, it was probably a tall, prominent cuspid. A very weak anterior cingulid (= precingulid) is present. Overall, the lingual margin of the talonid is somewhat higher than the labial margin. The talonid basin is relatively shallow. Of the talonid cuspids, the cuspid occupying the position of the hypoconulid (at the distolingual corner of the tooth) is the tallest. However, the hypoconid and entoconid are both worn. Despite this wear, a preentocristid with a distinct carnassial notch appears to be identifiable. The cristid obliqua terminates at approximately the midpoint of the protocristid, and also exhibits a distinct carnassial notch. An extremely faint, vestigial posterior cingulid (= postcingulid) appears to be present, below the crest connecting the hypoconid and hypoconulid (the posthypocristid). On m4, the massive protoconid dominates the trigonid. The paraconid and metaconid are reduced to small swellings at the lingual ends of the paracristid and protocristid, respectively. As on m3, a vestigial anterior cingulid is present. The talonid is similar to m3 in that the lingual margin is higher than labial margin, the talonid basin is shallow, and the talonid cuspids are weakly developed. As on m3, there appears to be a carnassial notch in both the preentocristid and the cristid obliqua, and an extremely faint posterior cingulid appears to be present.

#### Mandible

Both left and right mandibles are preserved (Figs 12–15 and 17–19). Both are largely complete except for damage anteriorly, at the anterior end of the mandibular symphysis. As a result of this, no incisors are preserved (except for one questionable tooth fragment), but there is evidence of at least two incisor alveoli. Both dentaries preserve c1 p1-3 m1-4 (described above). There is no diastema between c1 and p1, nor between p1 and p2. The retromolar space between m4 and the coronoid process is relatively long, being slightly shorter than the length of m4. The anterior margin of the coronoid process is mediolaterally thick and forms a distinct coronoid crest, whilst the dorsal margin is broken on both mandibles. The posterior margin of the coronoid process is mediolaterally thin, and slightly concave posteriorly.

There are two mental foramina on the lateral side of the dentary: the anterior one is positioned below the anterior root of the p2, whilst the posterior one is below the anterior root of m2. A short sulcus extends anteriorly from the posterior mental foramen. Overall, the mandibular body is robust and deep. The lateral surface of the body is not flat, but rather is somewhat concave. The masseteric fossa is well-excavated, and its ventral border forms a distinct shelf posteriorly (the posterior shelf of the masseteric fossa [38]). The condylar process is transversely wide. The mandibular condyle itself is oriented posterodorsally; in mediolateral view, it is slightly lower than the occlusal surface of m4.

The medial surface of the mandibular body is convex. The mandibular symphysis is unfused, and extends posteriorly to a point approximately below the midpoint of p3. Immediately posterior to the symphysis, the ventral margin of the body forms a distinct, medially-directed ridge or thickening; this ridge is much better developed than in *Didelphis*. The ridge has two distinct parts: an anterior part that begins immediately posterior to the symphysis and extends posteriorly to below the anterior root of m4, and a posterior crest that starts from level with the posterior margin of m4 and expands into a medially inflected lamina, namely the medially inflected angular process characteristic of metatherians [6, 115]. Based on evidence from the jaw musculature of *Didelphis* [116], the anterior part of this ridge may represent the attachments of the genioglossus, geniohyoideus and anterior belly of the digastric (and perhaps the mylohyoideus posteriorly), whilst the posterior part may reflect the attachment of the superficial masseter (and perhaps the mylohyoideus anteriorly), where it wraps around the ventral margin of the mandibular body.

#### Atlas

**Fig 20:**
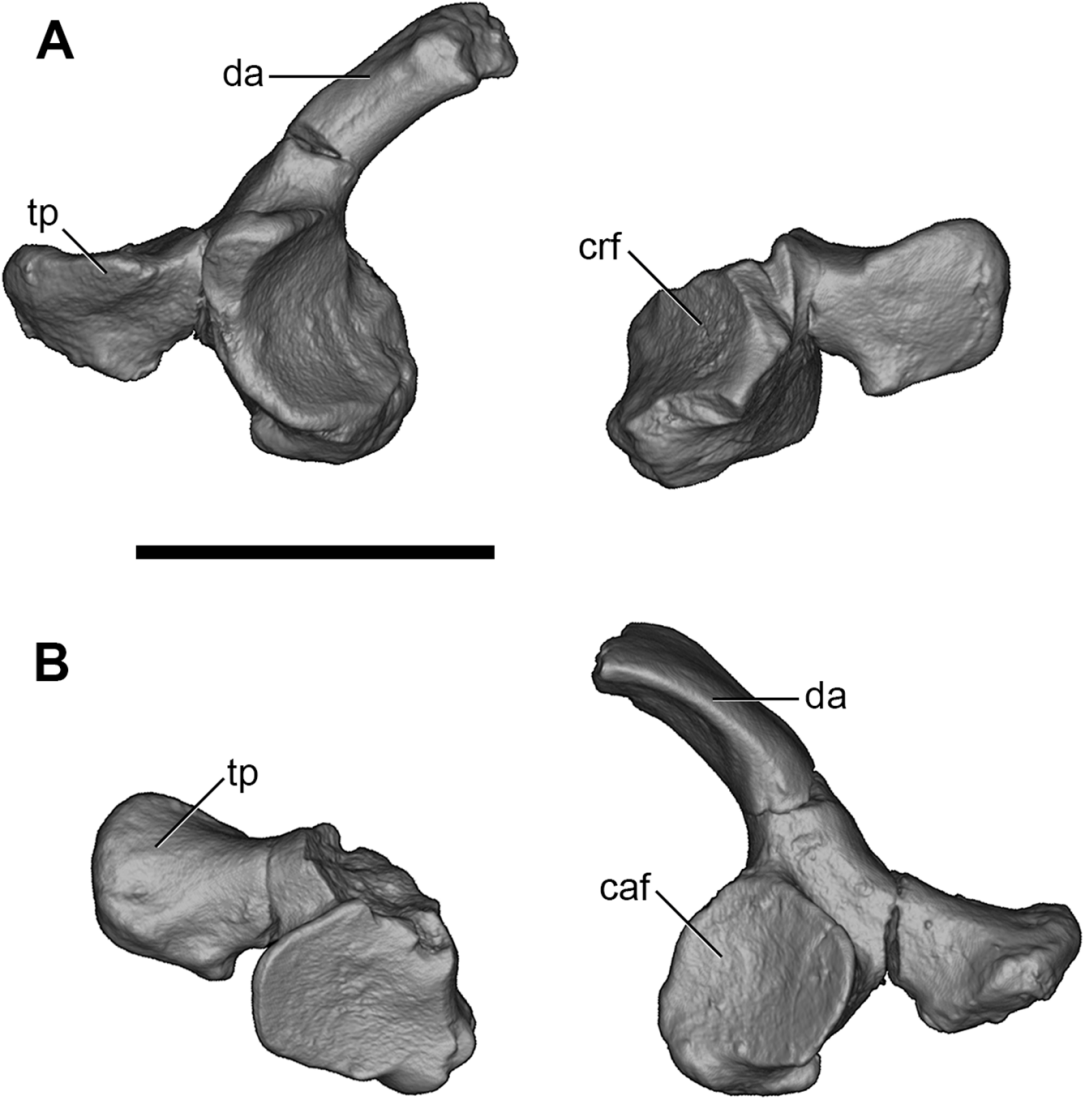
Volume-rendered CT images of atlas vertebra (C1) of holotype of *Anatoliadelphys maasae* (AÜJM 2002–25). **A**, anterior view; **B**, posterior view. Abbreviations: caf, caudal articular facet (= postzygapophysis); crf, cranial (exoccipital) articular facet (= prezygapophysis); da, dorsal arch; tp, transverse process. Scale bar = 1 cm.

The atlas is only partially preserved (Fig 20).

The right half is relatively complete, and preserves the cranial (exoccipital) articular facet or prezygapophysis (for articulation with the occipital condyles) and caudal articular facet or postzygapophysis (for articulation with the axis), transverse process, and right half of the dorsal (neural) arch. The left fragment comprises the caudal articular facet and transverse process only. The cranial articular facet is concave. The preserved portion of the dorsal arch shows no evidence for an atlantal foramen. An enclosed transverse foramen also appears to be absent, but there is a well-developed groove between the ventrolateral margin of the cranial articular facet and the base of the transverse process on the right side, resembling the morphology observed in *Didelphis* [46] and also the alar notch of the dog [57]. A similar, well-developed groove is present posteriorly, between the caudal articular facet and the transverse process. The right transverse process is complete; compared to that of *Didelphis*, it is more slender in AÜJM 2002–25, and it does not extend posteriorly as far as the level of the caudal articular facet; however, the transverse processes of the atlas of *Didelphis* are particularly well-developed.

#### Axis

**Fig 21:**
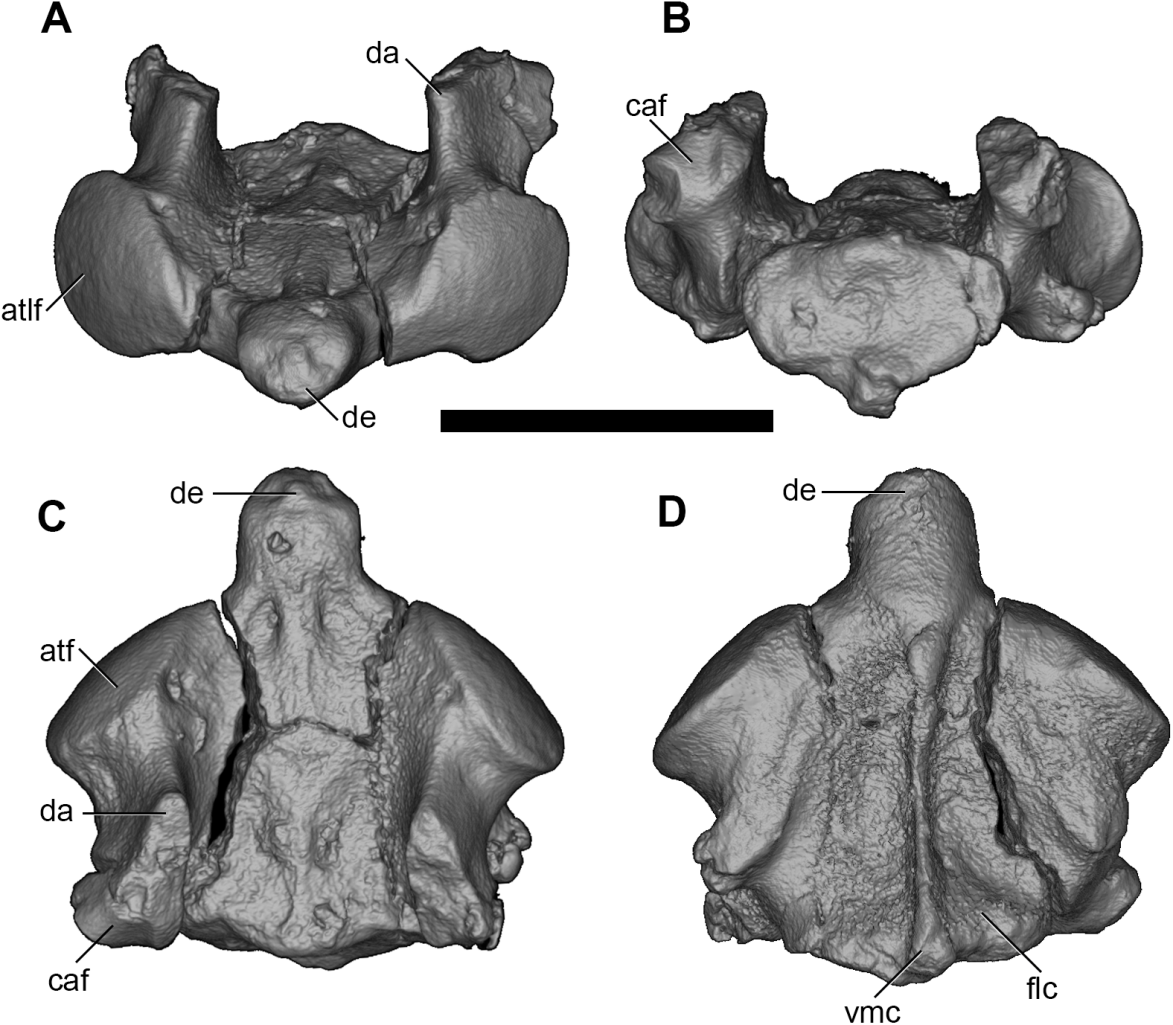
Volume-rendered CT images of axis vertebra (C2) of holotype of *Anatoliadelphys maasae* (AÜJM 2002–25). **A**, anterior view; **B**, posterior view; **C**, dorsal view; **D**, ventral view. Abbreviations: atlf, atlantal facet (= prezygapophysis); caf, caudal articular facet (= postzygapophysis); da, dorsal arch; de, dens; flc, fossa for longus colli muscles; vmc, ventral median crest. Scale bar = 1 cm.

The axis is only partially preserved, with the dorsal arch and transverse processes broken away (Fig 21).

The atlantal (cranial) articular facet (= prezygapophysis) and dens are not connected, but are instead separated by a short and shallow sulcus. Due to the breakage to the left and right transverse processes, it is not possible to determine whether the transverse foramen was fully enclosed by bone or incomplete. In ventral view, a prominent fossa for the longus colli muscle (an important depressor of the head) is present bilaterally, either side of a prominent ventral median crest that extends from the posterior (caudal) margin of the axis to the dens. A similar morphology has been reported in the early Palaeogene marsupialiforms *Pucadelphys* and *Mayulestes* [39, 45].

#### Other cervical vertebrae

**Fig 22:**
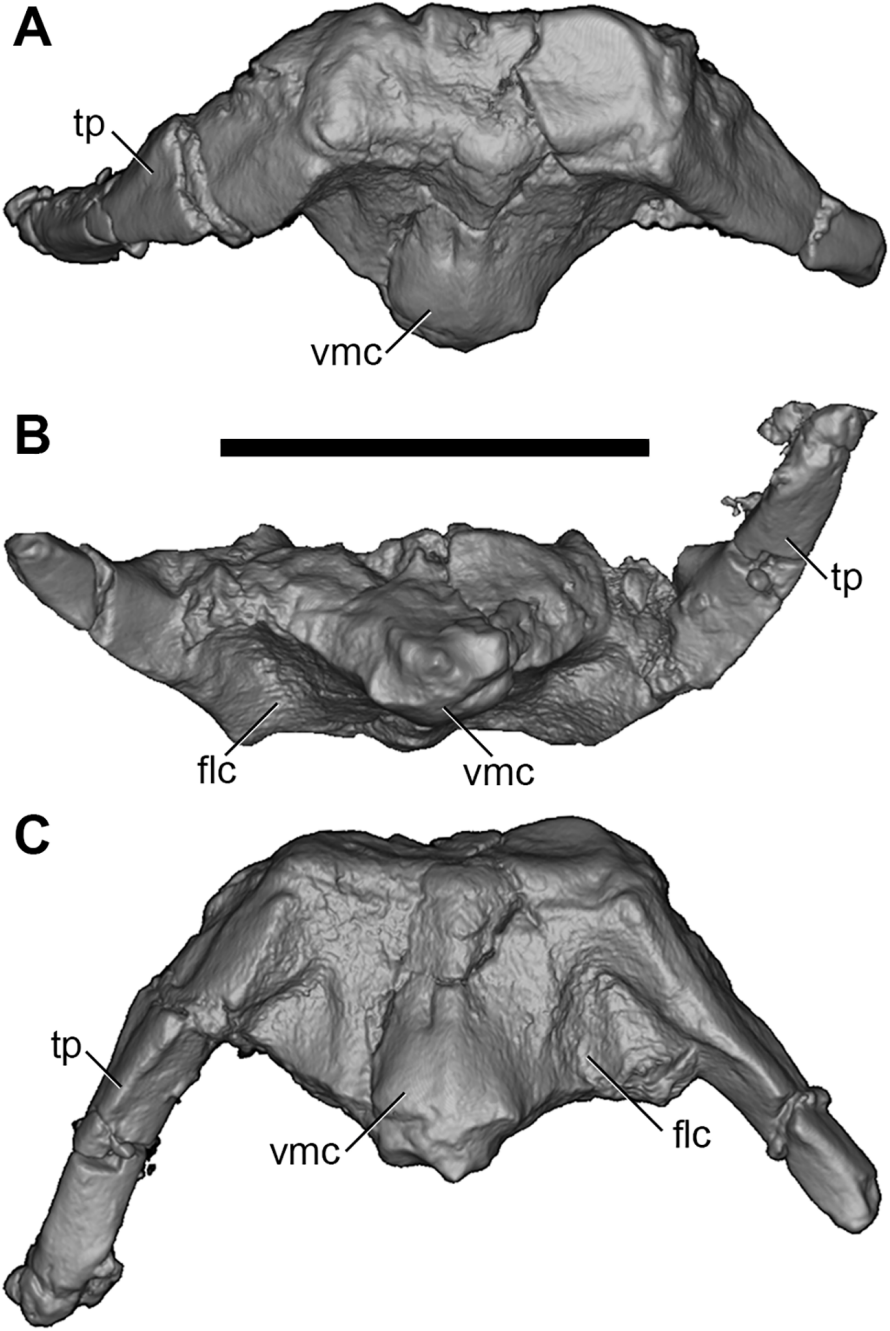
Volume-rendered CT images of cranial vertebra C3 of holotype of *Anatoliadelphys maasae* (AÜJM 2002–25). **A**, anterior view; **B**, posterior view; **C**, ventral view. Abbreviations: flc, fossa for longus colli muscles; tp, transverse process; vmc, ventral median crest. Scale bar = 1 cm.

An articulated series of five cervical vertebrae (C3-7) are preserved in AÜJM 2002–25 (Fig 1). However, most comprise the vertebral centra and partial neural arches only; C3 is one of the better preserved (Fig 22).

Similar to the axis vertebra (C2), there is a prominent fossa for the longus colli muscle present bilaterally on the ventral surface of vertebra C3, as also seen in *Mayulestes* [39]. The articulations between the vertebrae occur both on the vertebral centra and on the zygapophyses. In all five cervical vertebrae, the articular surfaces on the centra are flat. C7 bears a pair of transverse foramina.

#### Thoracic vertebrae

Nine vertebral centra preserved in AÜJM 2002–25 can be identified as coming from the thoracic region (Fig 1). The centra are mostly damaged, and they do not preserve any diagnostic morphology that would definitively identify them as coming from the thoracic series, such as long neural processes, or articular facets for ribs. However, given that the complete series of cervical and lumbar vertebrae are known for this specimen, by elimination they must represent thoracic vertebrae. Marsupials consistently have 19 thoracolumbar vertebrae, and this is likely also plesiomorphic for Metatheria as a whole [117]; given the presence of six lumbar vertebrae (see below), this implies that *Anatoliadelphys* would originally have had a total 13 thoracic vertebrae.c

#### Lumbar vertebrae

**Fig 23:**
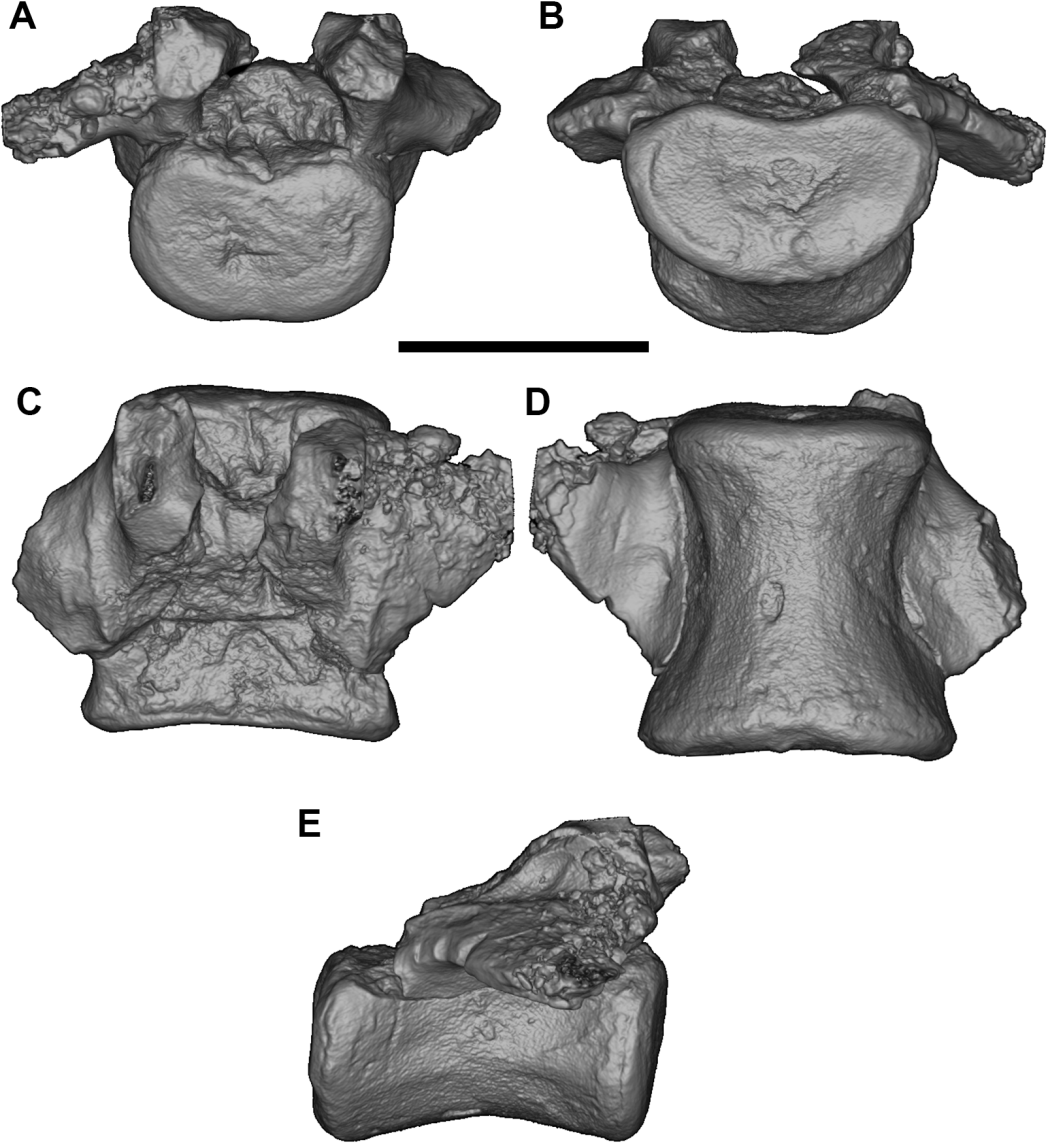
Volume-rendered CT images of lumbar vertebra L5 of holotype of *Anatoliadelphys maasae* (AÜJM 2002–25). **A**, anterior view; **B**, posterior view; **C**, dorsal view; **D**, ventral view; **E**, right lateral view. Scale bar = 1 cm.

A complete set of six articulated lumbar vertebrae (L1-6) are preserved (Fig 1), with L1, L2, L3 and L4 represented by the vertebral centrum alone. L5 is one of the best preserved of these (Fig 23): the tips of the transverse processes are broken, and the dorsal (neural) arch is partially preserved.

On L6, the transverse process is complete on one side but broken on the other, the (dorsal) neural arch is present (although the neural process is missing), and the pre- and post-zygapophyses are broken.

#### Sacrum

**Fig 24:**
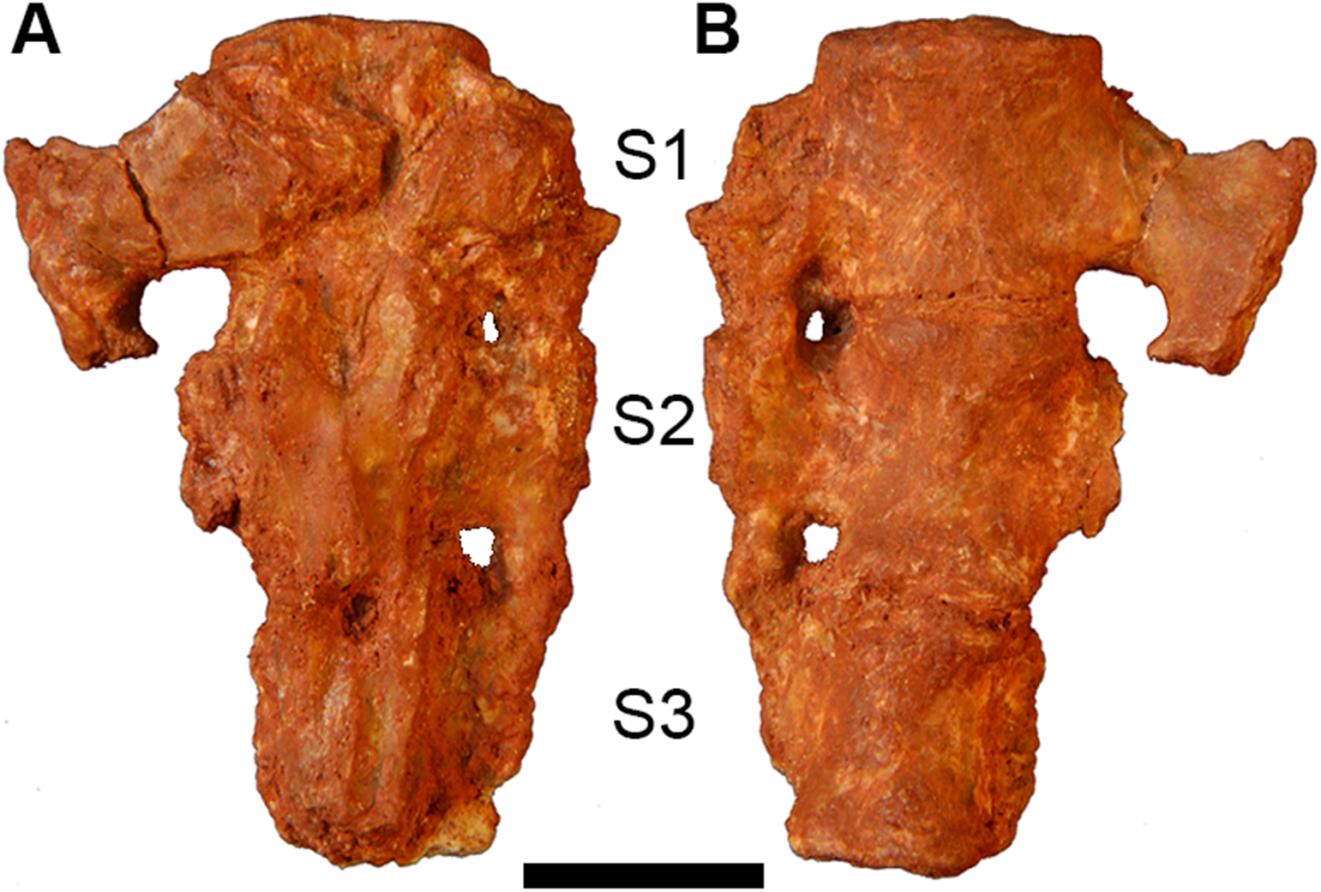
Photographs of sacrum of holotype of *Anatoliadelphys maasae* (AÜJM 2002–25). **A**, dorsal view; **B**, ventral view. Abbreviations: S1, first sacral vertebra; S2, second sacral vertebra; S3, third sacral vertebra. Scale bar = 1 cm.

AÜJM 2002–25 preserves the sacrum, which comprises three partially fused sacral vertebrae (Fig 24). The sacrum is robust, but damage to the sacral vertebrae means that relatively little else can be inferred about its morphology, including how many of the vertebrae articulated with the ossa coxae (innominate bones).

#### Caudal vertebrae

**Fig 25:**
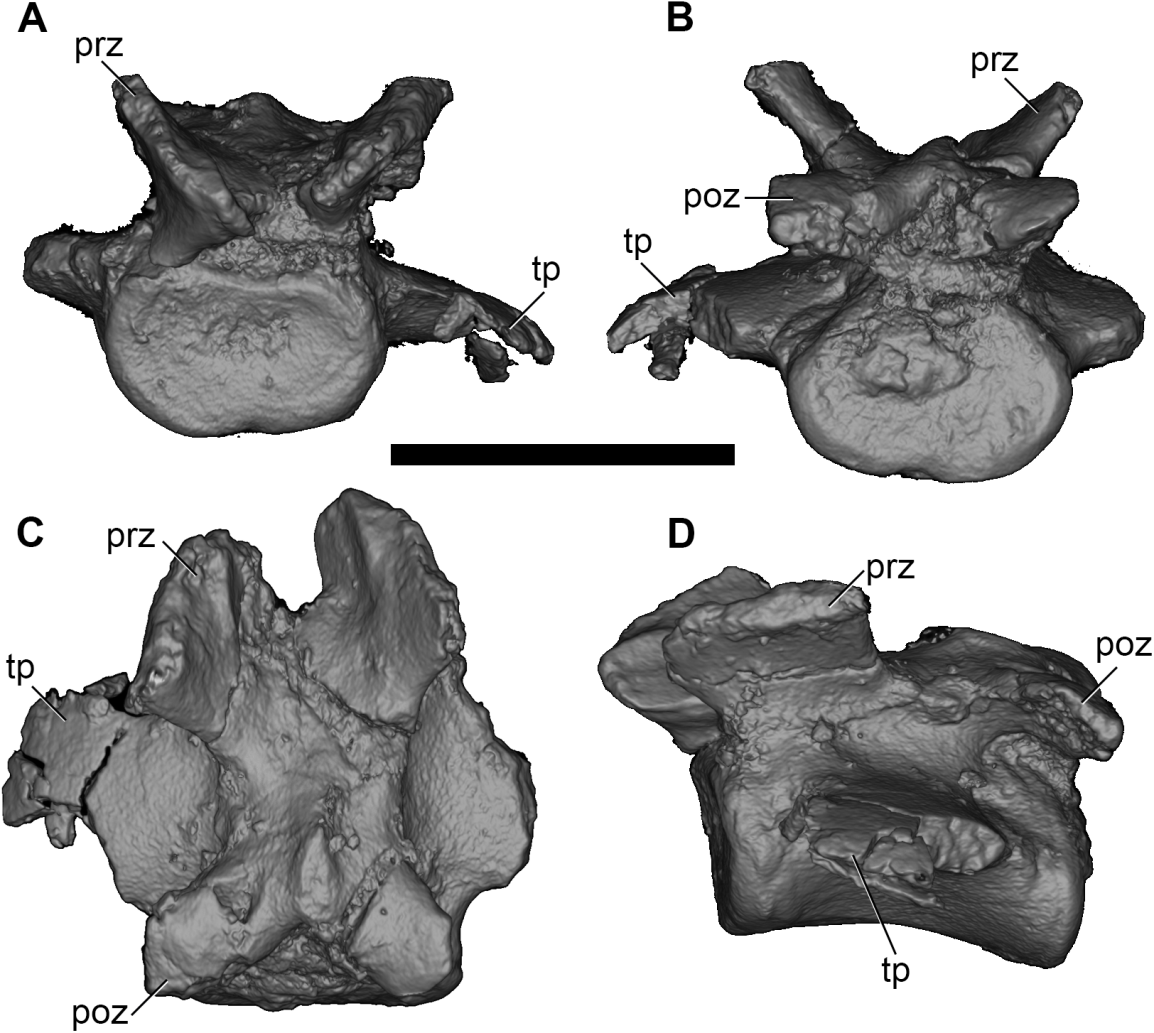
Volume-rendered CT images of caudal vertebra Ca3 of holotype of *Anatoliadelphys maasae* (AÜJM 2002–25). **A**, anterior view; **B**, posterior view; **C**, dorsal view; **D**, left lateral view. Abbreviations: prz, prezygapophysis; poz, postzygapophysis; tp, transverse process. Scale bar = 1 cm.

Five vertebrae can be identified as proximal caudal vertebrae, based on the size of their transverse processes (Fig 1); of these, Ca3 is one of the best preserved (Fig 25).

A single vertebra can be identified as a caudal vertebra belonging to the middle part of the series, based on its long vertebral body and lack of transverse processes. A group of vertebrae is here identified as representing distal caudal vertebrae because of their diminishing vertebral length. The total number of elements identified as caudal vertebrae is nine, but we identify multiple gaps in the size rank of the vertebral bodies, and so the total caudal vertebral count is clearly much greater than nine, probably at least 12. The preserved caudal vertebrae are quite broad mediolaterally, suggesting a relatively thick tail. However, it is impossible to accurately estimate the total length of the tail relative to body size.

#### Sternum

**Fig 26:**
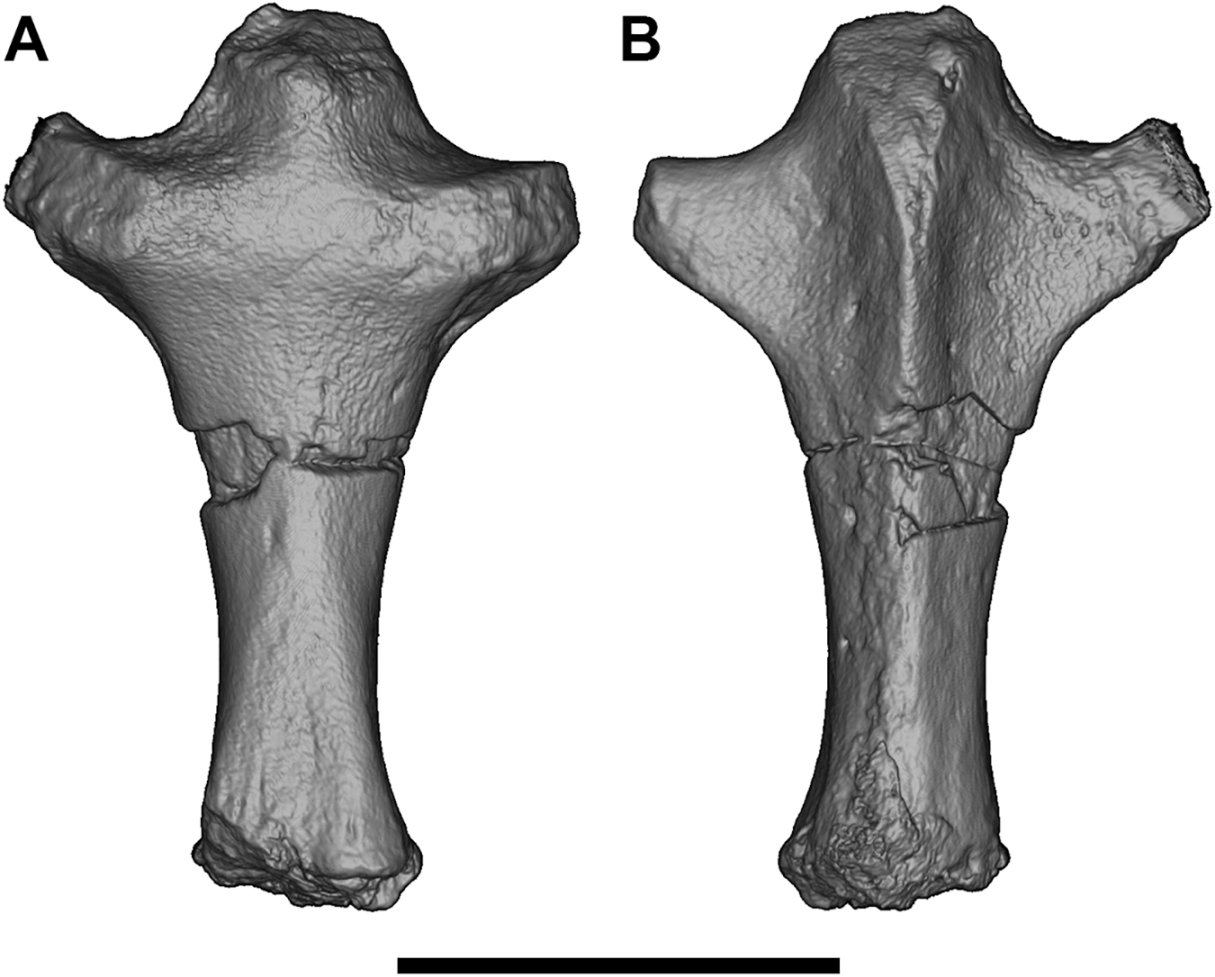
Volume-rendered CT images of manubrium of holotype of *Anatoliadelphys maasae* (AÜJM 2002–25). **A**, dorsal view; **B**, ventral view. Scale bar = 1 cm.

The manubrium and four sternebrae are preserved (Fig 1). The morphology of these elements closely resembles those of marsupials, with the manubrium (Fig 26) having prominent lateral processes anteriorly for articulation with the first pair of ribs, and a distinct ventral keel that is particularly prominent towards the anterior end, suggesting the presence of a well-developed M. pectoralis [40, 45, 118].

#### Scapula

**Fig 27:**
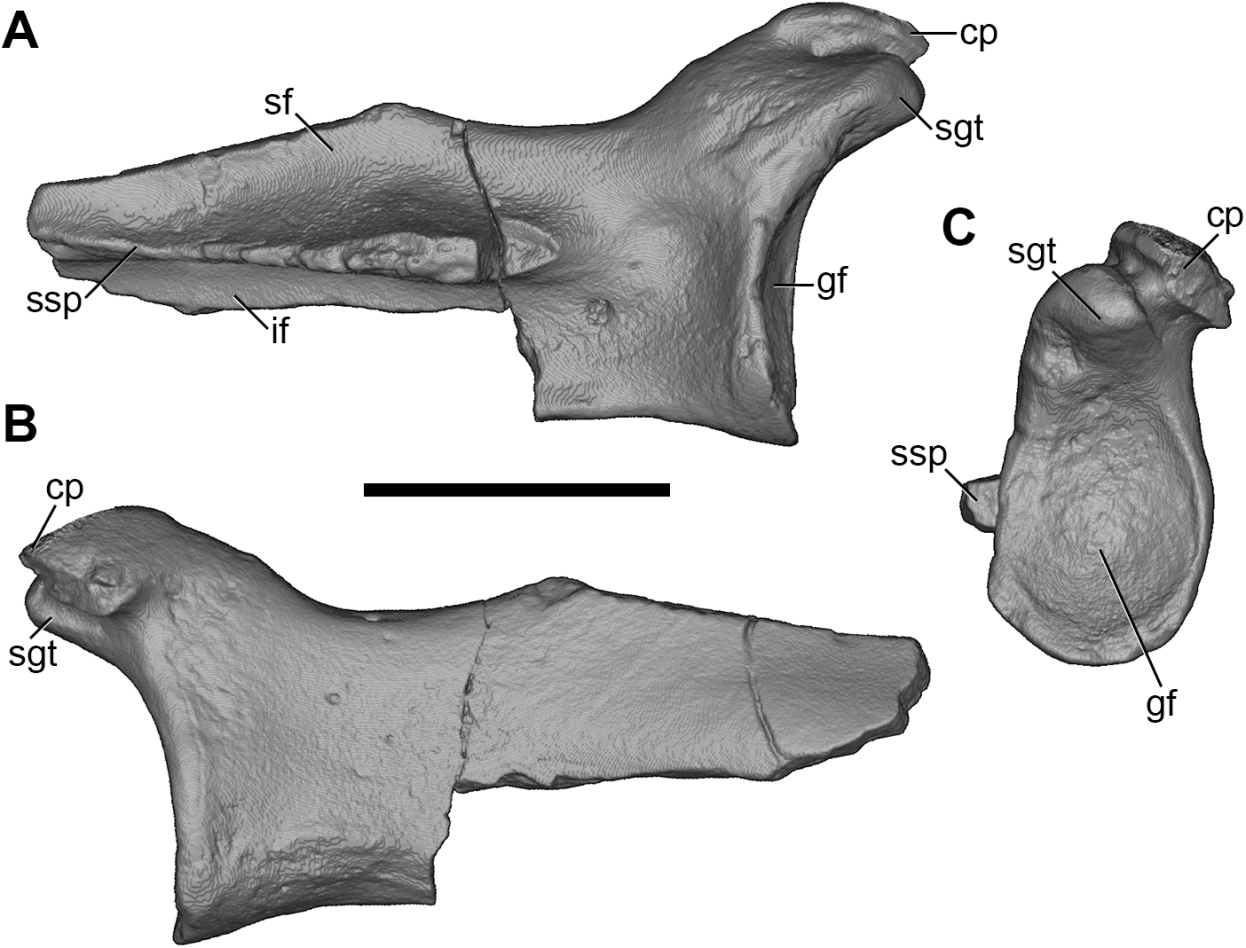
Volume-rendered CT images of right scapula of holotype of *Anatoliadelphys maasae* (AÜJM 2002–25). **A**, lateral view; **B**, medial view; **C**, distal view. Abbreviations: cp, coracoid process; gf, glenoid fossa; if, infraspinous fossa; sf, supraspinous fossa; sgt, supraglenoid tubercle; ssp, scapular spine. Scale bar = 1 cm.

Partial left and right scapulae are present, but the glenoid is the only region intact on both sides (Fig 1). The right scapular fragment also preserves parts of the cranial and caudal borders (Fig 27).

The glenoid is a cranially-elongated oval shape. Unlike the condition in *Didelphis*, the coracoid process is distinctly separated from the supraglenoid tubercle by a deep, narrow sulcus, which is visible on both medial and lateral surfaces. The tip of the coracoid process is broken in almost identical locations on both the left and right scapular fragments, and so it is difficult to determine exactly how far it projected medially. The origin of the coracoid process is positioned relatively laterally (towards the midline of the glenoid cavity), and overall the base is broader than in *Didelphis*. Most of the scapular spine is missing, as is the entire acromion, and so it is not possible to infer the morphology of the hamatus process. Similarly, very little of the caudal border and none of vertebral border is preserved, and so the overall shape of the scapula (more rectangular versus more triangular) is uncertain. The posterior region of the neck lacks well-developed fossae for the M. teres minor and the caput longum of the M. triceps brachii [43, 57, 58]; Muizon (39) reported a similar morphology in *Mayulestes* and sparassodonts.

#### Humerus

**Fig 28:**
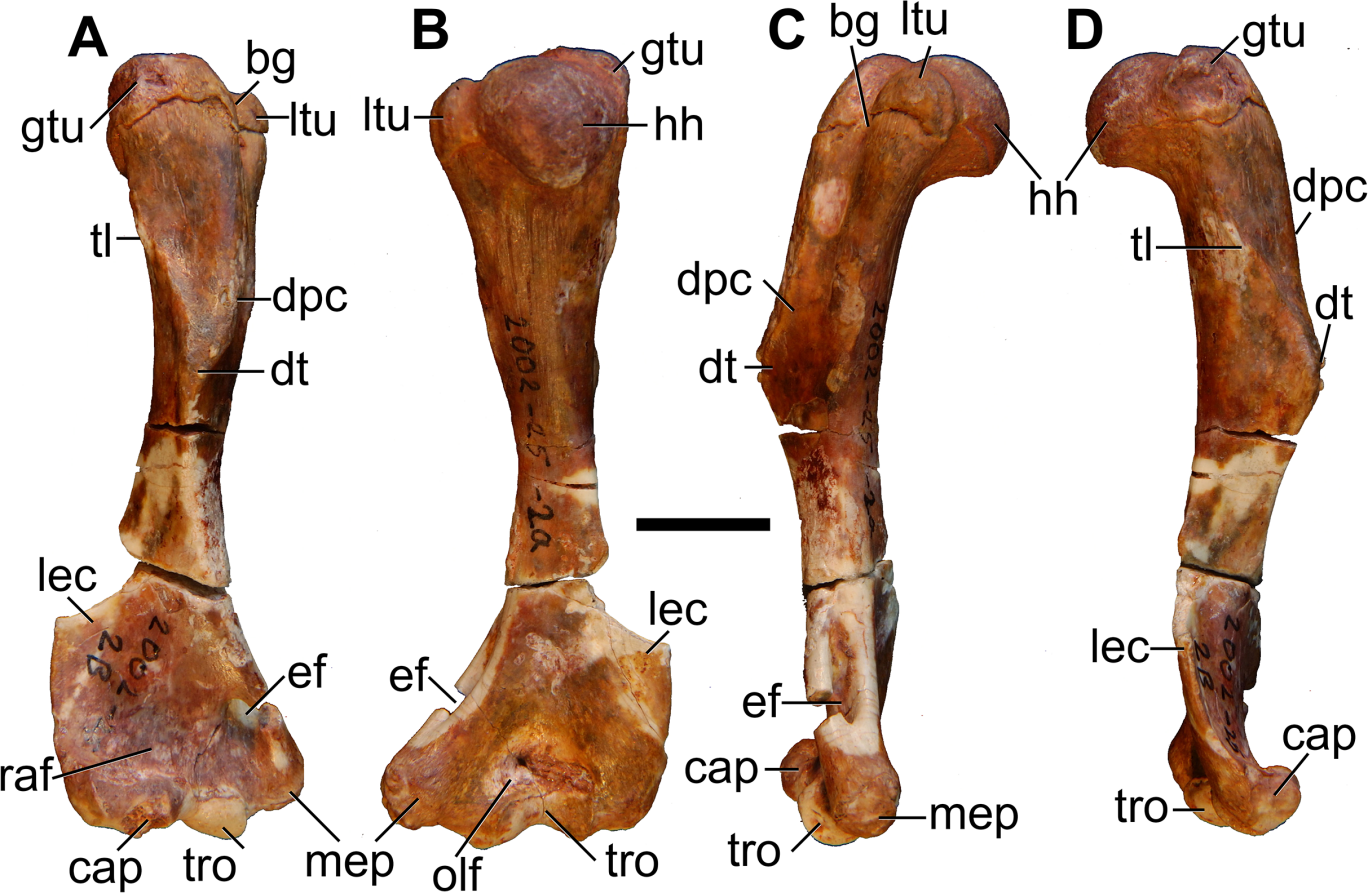
Photographs of right humerus of holotype of *Anatoliadelphys maasae* (AÜJM 2002–25). **A**, cranial (anterior) view; **B**, caudal (posterior) view; **C**, medial view; **D**, lateral view. Abbreviations: bg, bicipital groove; cap, capitulum; dpc, deltopectoral crest; dt, deltoid tuberosity; gtu, greater tuberosity; hh, humeral head; lec, lateral epicondylar crest; ltu, lesser tuberosity; mep, medial epicondyle; olf, olecranon fossa; raf, radial fossa; tl, tricipital line; tro, trochlea. Scale bar = 1 cm.

Both humeri are preserved in AÜJM 2002–25, but they differ in preservation. The right humerus is largely intact (Fig 28), but the left appears to have undergone plastic deformation of the distal part during fossilisation.

The intertubercular sulcus between the greater and lesser tubercles is deep. The head (= condyle) is separated from both tubercles by a shallow sulcus extending around its anterior perimeter. The separation between the head and the lesser tubercle appears to be more pronounced than that of *Didelphis*. In addition, the proximal portion of the head is more convex (almost spherical) when compared to that of *Didelphis*. The humeral head and greater tubercle are similar in height, as is usual for metatherians [119]. The head is positioned further posteriorly relative to the humeral shaft (i.e. it is distinctly “beaked” [47]) than that of *Caluromys*, but is similar in this regard to *Didelphis*.

In proximal view, the greater tubercle appears elongated and has an oblique anteromedial-posterolateral orientation, similar to that seen in *Didelphis*. The lesser tubercle is separated from the humeral head by a shallow groove, which opens anterolaterally into the intertubercular sulcus. The lesser tubercle is almost half the size of the greater tubercle; the lesser tubercle is also more rounded, and it is oriented nearly perpendicularly relative to the major axis of the greater tubercle. The lesser tubercle is lower than greater tubercle.

In mediolateral view, the shaft of the humerus appears weakly sigmoidal in shape, as in *Didelphis*, whereas that of *Caluromys* appears almost straight [43]. In medial view, the intertubercular sulcus (= bicipital groove) is pronounced. The sulcus is located on the anteromedial margin of the humerus. It is deepest proximally, and gradually becomes shallower distally. It extends as a distinct groove from the tubercles to almost the midpoint of the humerus. The medial wall of the groove is formed by the lesser tubercle crest, referred to as the crista tuberculi minoris in the dog [57]. At the distal end of the groove, the lesser tubercle crest first makes a very sharp medial projection as the teres tuberosity and then gradually tapers into the shaft. Interestingly, there appears to be no distinct lesser tubercle crest in *Didelphis*, and yet the teres tuberosity is prominent. The well-developed lesser tubercle crest in AÜJM 2002–25 indicates that the medial head of the M. triceps brachii was very well-developed, allowing powerful forearm extensions.

Laterally, the other wall of the intertubercular groove is formed by the deltopectoral crest, which extends slightly more distally than does the lesser tubercle crest. The deltopectoral surface is the area bound by the deltopectoral crest (medially) and the tricipital line (posterolaterally). In AÜJM 2002–25, the deltopectoral surface is triangular in shape, slightly concave, and faces anterolaterally. Compared to *Didelphis*, this surface is shorter, and relatively wider, and the tricipital line is more obvious. In *Didelphis*, the course of the tricipital line from its origin at the posterolateral edge of the greater tubercle to its fusion with deltopectoral crest is hard to identify in parts, and at times is only visible as weak muscle scars. In AÜJM 2002–25, the proximal half of the tricipital line (to which the M. triceps brachii caput lateralis attaches) runs straight distally, whereas the distal half bends medially and runs towards the deltopectoral crest, where together they form a ‘V’ shape. Throughout its course, there is no interruption in the definition of the tricipital line. Posteriorly, the fossa on the humeral shaft posterodistal to the lesser tubercle is shallow and runs two-thirds of the length of the lesser tubercle crest.

Distally, the most notable feature on the humerus is the mediolateral expansion of the distal end. This can be quantified as the Humeral Epicondylar Index (HEI = maximum distal width of the humerus/maximum length of the humerus [76]), which is 0.36 for AÜJM 2002–25. This HEI value is greater than in dasyurids and most didelphids (including *Didelphis* and *Caluromys*), but is similar to those of the didelphids *Marmosa* and *Micoureus* [76]. Parts of the lateral epicondylar crest (or supinator ridge) are broken in both the left and right humeri, but this crest was clearly well-developed. On the right humerus, only a small portion of the proximal aspect of the lateral epicondylar crest is broken, and so it can be seen that the crest spans approximately one-third of the total humeral length. However, the breakage on both left and right sides render the exact shape of the crest unclear.

In cranial view, the lateral epicondylar surface is concave. Distally, the capitulum is damaged on the right humerus, but intact on the left, which indicates a relatively spherical articular surface. There is a slight asymmetry between left and right humeri in terms of the depth of the radial fossa, with this fossa shallower on the right than on the left. The left radial fossa seems deeper than that of *Didelphis*, but this may be the result of post-mortem deformation. There is no continuous lateral extension (or “tail”) of the capitulum in AÜJM 2002–25; instead there is a distinct groove between the capitulum and the keel that forms the distolateral corner of the humerus. A capitular tail is found in *Didelphis* and other large didelphids (see character 56 of Horovitz and Sánchez-Villagra [119] and character 64 of Flores [40]), and also many fossil metatherians [28, 59], and it was found to be a synapomorphy of Metatheria by O’Leary et al. [98]; its absence in AÜJM 2002–25 is presumably a secondary loss. An oval-shaped entepicondylar foramen is present, but it is smaller than that seen in *Didelphis*. The trochlea has moderately well-defined crests, but is not as medially deflected as it is in *Didelphis*. In caudal view, the olecranon fossa is deeper than in *Didelphis*, but shallower than in *Dasyurus*.

#### Ulna

**Fig 29:**
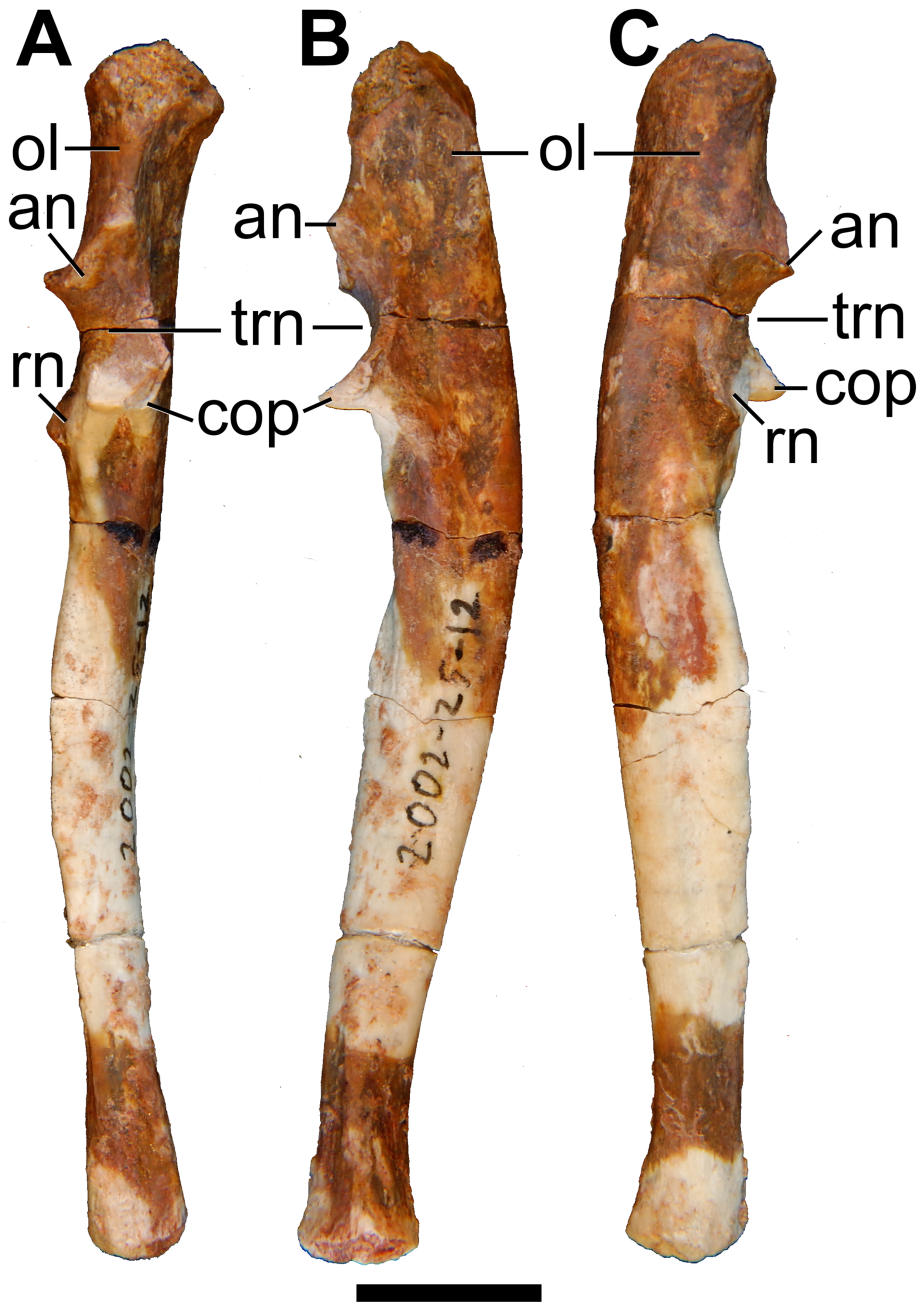
Photographs of right ulna of holotype of *Anatoliadelphys maasae* (AÜJM 2002–25). **A**, cranial (anterior) view; **B**, caudal (posterior) view; **C**, medial view; **D**, lateral view. Abbreviations: an, anconeal process; cop, coronoid process; ol, olecranon process; rn, radial notch; trn, trochlear notch. Scale bar = 1 cm.

Both left and right ulnae are preserved. The right ulna is practically complete, except that the proximal end is slightly damaged, and the styloid process is missing from the distal end (Fig 29). The bone is broken into several pieces, but the pieces fit together almost perfectly. The left ulna, however, is represented by the proximal half only.

The ulna is the longest bone of the forelimb in AÜJM 2002–25. In cranial view, the shaft is distinctly bowed, such that the medial border is concave and the lateral border is convex. The ulna of *Didelphis* also appears bowed, but the overall degree of curvature is much less than that in AÜJM 2002–25. The curvature observed in AÜJM 2002–25 does not appear to be caused by skeletal pathology or the fossilisation process. In mediolateral view, the ulna as a whole shows a sigmoidal curvature, similar to that seen in *Didelphis*.

The Olecranon Process Length Index (OPLI = olecranon process length/total ulnar length [76]) in AÜJM 2002–25 is 0.18, but this is only approximate as the proximal end of the olecranon process is damaged and the styloid process is missing from the distal end. Nevertheless, this OPLI value is similar to that of *Didelphis* and much greater than that of *Caluromys* [76].

In cranial view, the olecranon process does not appear strongly medially inflected, nor does it appear medially twisted as it is in *Asiatherium* [120]. The curve of the posterior border of the olecranon process is less than in *Caluromys*, but similar to that seen in *Didelphis* (see character 71 of Flores [40]). The depression on the medial side of the proximal ulna is the fossa for the M. flexor carpi ulnaris [39]; this fossa is quite deep in *Caluromys* and *Mayulestes*, but varies in depth in *Didelphis*, although it is never as deep as it is in *Caluromys*. In AÜJM 2002–25, the fossa for the M. flexor carpi ulnaris is neither long nor deep (as it is in *Caluromys*), but it falls within the range of morphologies seen in *Didelphis*.

In medial view, the distal half of the trochlear notch is deeper and more concave (similar to *Didelphis*) than the proximal half, which is shallower and low (as in *Caluromys*). The anconeal process (the beak of the olecranon) is moderately salient, but not as high as in *Didelphis*. The width of the trochlear notch is similar to that seen in *Didelphis*. The orientation and the prominence of the coronoid process are also very similar to the morphology observed in *Didelphis*.

The radial notch is distolateral to the trochlear notch. In *Didelphis*, the boundary between the radial notch and the lateral humeral articular surface within the trochlear notch are clearly demarcated by a salient ridge. In AÜJM 2002–25, however, these surfaces are almost continuous on the right ulna; the left shows a line of separation between the two surfaces, but it does not approach the morphology seen in *Didelphis*. The articular surface of the radial notch is more laterally facing than in *Didelphis*, in which it faces more anteriorly. The radial notch does not appear to extend onto the lateral border of the coronoid process, whereas it does so in *Didelphis* and *Caluromys*.

In cranial view, the fossa for the M. brachialis and M. biceps is quite deep and medially positioned, and it is just distal to the coronoid process. The fossa is proximodistally-elongated and surrounded by two ridges running from the medial and lateral borders of coronoid process. The lateral ridge separates the brachialis fossa from another slight depression, which Muizon [39] referred to as the ‘supinator fossa’ in *Mayulestes*. The lateral border of the supinator fossa is formed by a salient supinator crest in *Mayulestes* [39]. In AÜJM 2002–25, the lateral border of the supinator fossa is not raised into a crest or a ridge, but at the distolateral corner of the supinator fossa this lateral border rises to form the interosseous crest. The interosseous crest is salient and extends distally along the entire length of the ulnar shaft, forming the craniolateral border of the shaft. This crest is not as sharp in cross section as it is in *Caluromys* or *Didelphis*, but it is still sharper and more salient than the equivalent crest in *Sarcophilus*. Viewing the ulnar shaft laterally, there is a fossa for the M. abductor pollicis longus, caudal to the interosseous crest: this fossa is well-marked proximally, but distally it gradually becomes shallower and eventually disappears. The morphology of this fossa is more similar to the condition seen in *Didelphis* than in *Caluromys*, in which this fossa extends for practically the entire length of the ulnar shaft.

Medially, the pronator crest (the origin of the M. pronator quadratus) at the distal end of the diaphysis is shorter but more prominent than in *Didelphis*, but less salient than in *Caluromys*. As already discussed, the styloid process is not preserved.

#### Radius

**Fig 30:**
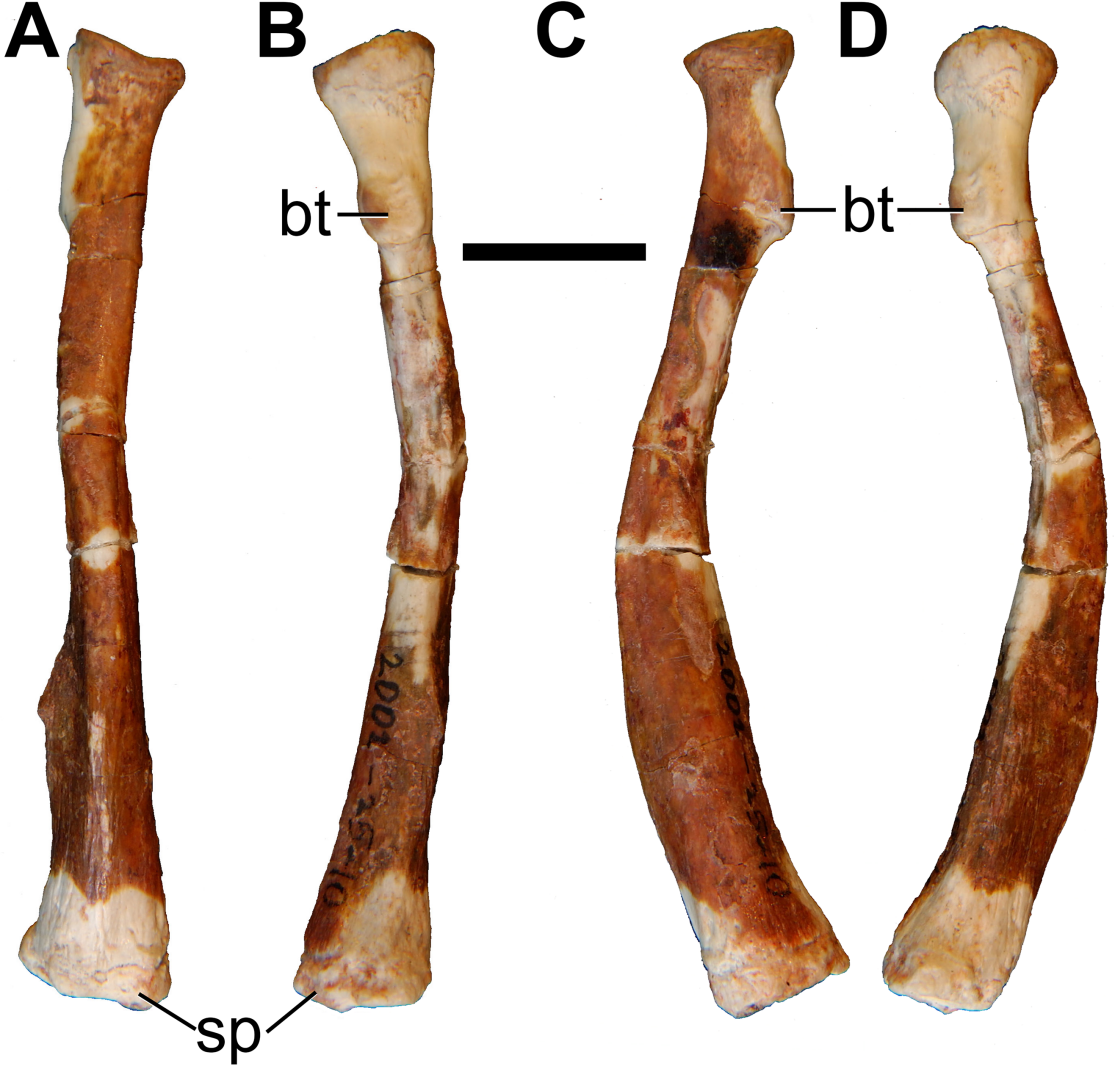
Photographs of right radius of holotype of *Anatoliadelphys maasae* (AÜJM 2002–25). **A**, cranial (anterior) view; **B**, caudal (posterior) view; **C**, medial view; **D**, lateral view. Abbreviations: bt, bicipital tuberosity; sp, styloid process. Scale bar = 1 cm.

Both radii are preserved. The right radius is almost complete; there is a major crack in the midshaft and the two sides do not match up either side of the crack, but evidently no more than a few millimetres of the shaft are missing (Fig 30). The left radius preserves the distal three-quarters of the bone; the portion from the distal portion of the bicipital tuber to the radial head is missing. Proximally, the radial head appears rounded in proximal view, similar in shape to those of *Didelphis* and *Caluromys*, whereas it is transversely wider and more oval-shaped in some other metatherians (e.g. *Asiatherium*, *Mayulestes*, *Pucadelphys*, some sparassodonts, *Thylacinus* [28, 39, 120]). However, the facet for the humeral capitulum is deeper than that of *Didelphis*, possibly reflecting the fact that the capitulum is more spherical in AÜJM 2002–25 than in *Didelphis*. The size and the shape of the ulnar articular facet on the caudal side of the radial head are similar to the morphology seen in *Didelphis*.

Along the shaft, the bicipital tuberosity is caudally very prominent, and is proportionately more salient than even the largest *Didelphis* specimens examined; this tuberosity is very weak in the fossil metatherian *Mayulestes* [39]. The bicipital tuberosity is proximodistally more distant from the radial head than in *Dasyurus*, but is similarly positioned to that of *Didelphis*. In medial view, there is a small but noticeable depression just anterior to the bicipital tuberosity. *Didelphis* lacks a similar depression, but *Caluromys* resembles AÜJM 2002–25 in this regard.

In caudal view, a ridge on the lateral side can be identified extending from approximately midpoint of the shaft to the proximal third. This ridge is the radial counterpart of the interosseous crest of ulna. A similar ridge can be identified in *Didelphis*, but the degree of cresting is much less than in AÜJM 2002–25. *Caluromys*, meanwhile, differs from AÜJM 2002–25 in that this interosseous crest extends along the entire radial shaft. Instead, the condition in AÜJM 2002–25 appears more similar to *Dasyurus* in this regard. Further distally, the ridge becomes identifiable again, forming the pronator crest. None of the *Didelphis* specimens examined for this study have a pronator crest as prominent as that in AÜJM 2002–25.

The cranial surface of the radial shaft is relatively flat, and is rectangular proximally. Both the medial and lateral borders of the anterior side merge around the midshaft, and then continue distally as a single well-defined lateral border. The lateral border terminates distally as the dorsal tubercle, which separates the sulcus for the tendons of the M. extensor digitorum communis (laterally) from the sulcus for the M. extensor carpi radialis (medially). Lateral to the M. extensor digitorum communis sulcus, there is no ridge marking the probable insertion of the M. abductor pollicus longus, and hence this muscle/tendon was probably not particularly strongly developed. *Didelphis* also lacks a prominent ridge for the M. abductor pollicus longus, but this ridge is very well-developed in *Caluromys* [39, 43]. Curvature of the radial shaft means that it is strongly convex cranially and concave caudally, to a greater extent than seen in *Didelphis*, but similar to that seen in the didelphid *Monodelphis* and the stem-marsupial *Pucadelphys* [43]. The cross section of the shaft is triangular throughout the length of the radius; however the apex of the triangle projects caudally in the proximal half, whereas it projects cranially in the distal half.

In distal view, the scapholunar articulation is oval and more concave than in *Didelphis*, and is also larger. The styloid process projects more vertically from the articular surface compared to the more gradual inclination seen in *Didelphis*. Viewed medially, the styloid process is almost hook-shaped, with a notch on its posterior margin; by contrast, in *Didelphis* it is straight, whereas in *Sarcophilus* it has a trochlear shape. The distal ulnar facet is restricted to the lateral margin of the distal radius, and is smaller than in *Didelphis*.

#### Os coxae

**Fig 31:**
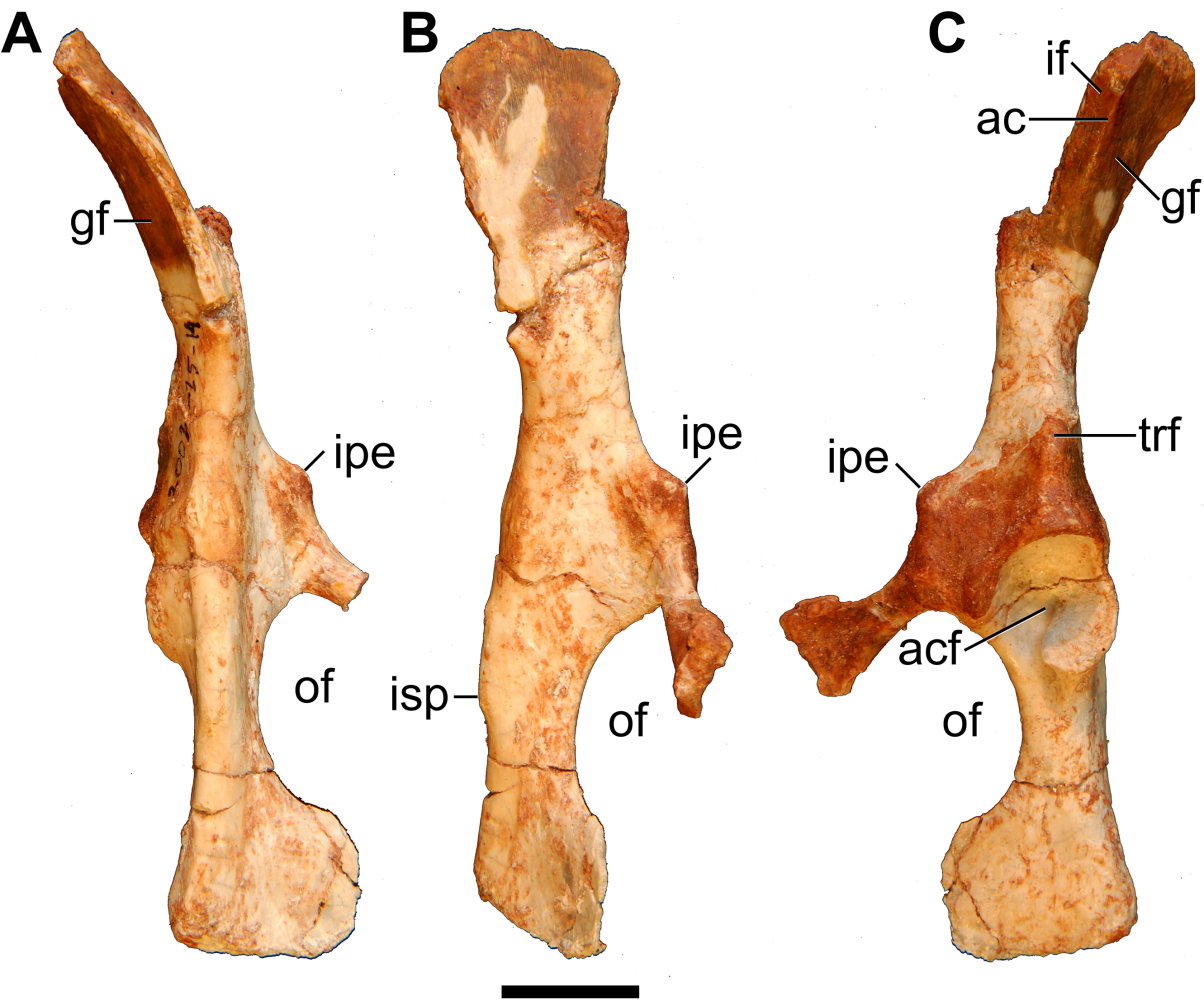
Photographs of left os coxae (innominate) of holotype of *Anatoliadelphys maasae* (AÜJM 2002–25). **A**, dorsal view. **B**, medial view. **C**, lateral view. Abbreviations: ac, acetabular crest; acf, acetabular foramen; gf, gluteal fossa; if, iliacus fossa; ipe, iliopubic eminence; isp, ischatic spine; of, obturator foramen; trf, tuberosity for M. rectus femoris. Scale bar = 1 cm.

Both ossa coxae (innominate bones) are preserved; they are both mostly complete, missing only the ischiopubic ramus and parts of the iliac crest (Fig 31). Damage to these elements and to the sacrum (Fig 24) means that it is unclear how many sacral vertebrae made contact with the ilium. The ilium is long, forming 61% of the total length of the os coxae; it is relatively flat and bladelike and flares laterally in dorsal view, as in dasyurids but unlike the condition in didelphids except *Metachirus* [39, 44, 48]. However, the iliac fossa does not appear as small in AÜJM 2002–25 as it is in dasyurids and *Metachirus* [44]. The iliopubic process (or eminence) is well-developed. The acetabulum is relatively spherical but somewhat elongated dorsoventrally. In dorsal view, there is a slight emargination of the dorsal acetabular rim, but the acetabulum is nevertheless fully enclosed dorsally. The acetabular notch is deep, and located on the caudoventral margin of the acetabulum. There is a distinct tuberosity for the M. rectus femoris anterior to the acetabulum, as in the fossil metatherians *Herpetotherium*, *Mayulestes* and *Pucadelphys*, whereas this is uncommon in extant marsupials, although it is seen in dasyurids [49, 119]. Damage means that the likely area of contact between the pubis and the epipubic bones cannot be identified.

#### Epipubic bones

Both epipubic (marsupial) bones are preserved intact (Fig 1); they appear relatively robust but are otherwise unremarkable and lack distinctive morphological features.

#### Femur

**Fig 32:**
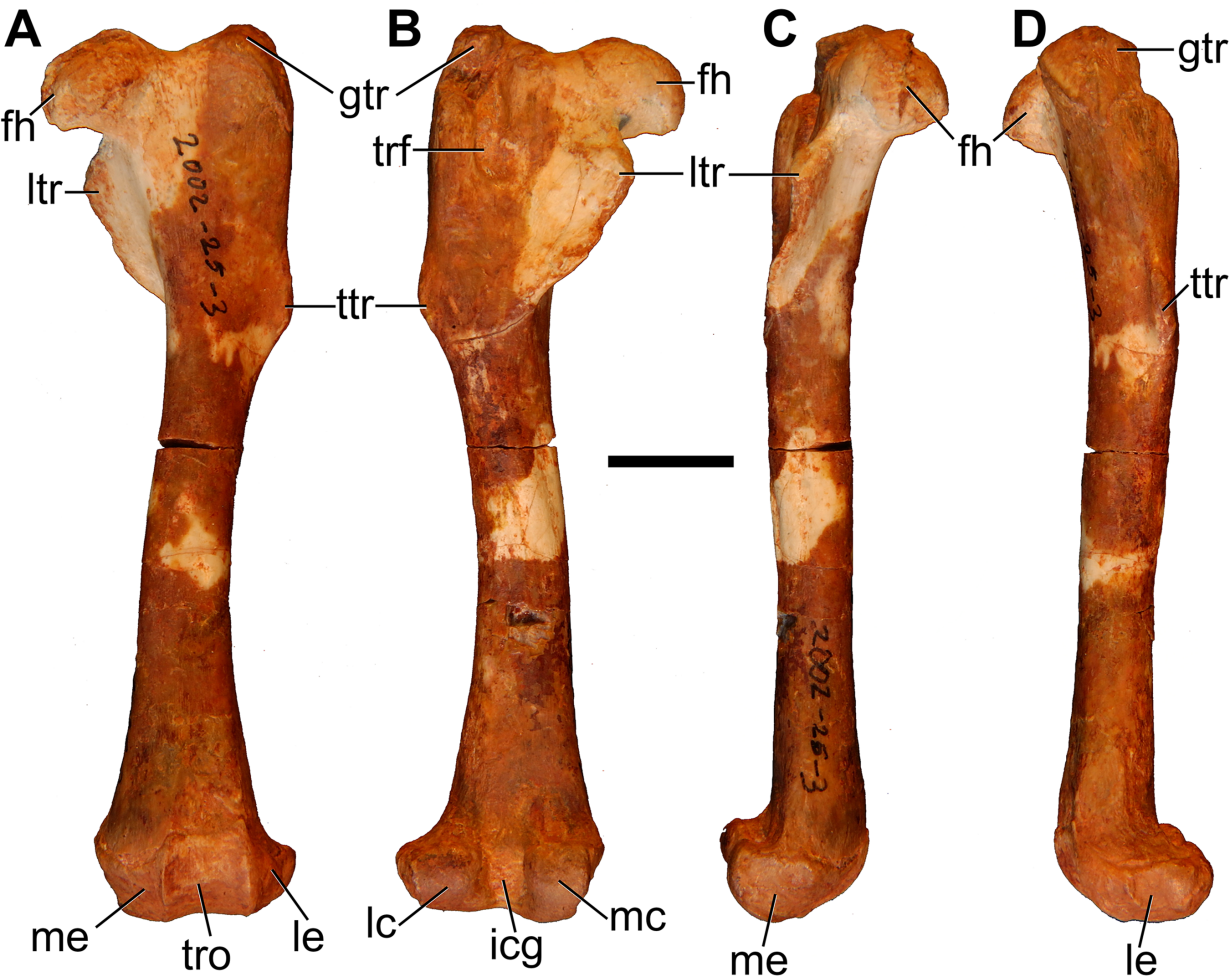
Photographs of left femur of holotype of *Anatoliadelphys maasae* (AÜJM 2002–25). **A**, cranial (anterior) view; **B**, caudal (posterior) view; **C**, medial view; **D**, lateral view. Abbreviations: fh, femoral head; icg, intercondylar groove; lc, lateral condyle; le, lateral epicondyle; ltr, lesser trochanter; mc, medial condyle; me, medial epicondyle; tro, trochlea; trf, trochanteric fossa; ttr, third trochanter. Scale bar = 1 cm.

Both femora are preserved. The left femur was in contact with the pelvis when AÜJM 2002–25 was excavated. However, in the process of preparing the specimen, it became clear that the femur had been displaced superiorly with respect to the os coxae during fossilisation, and the head was no longer in articulation with the acetabulum; thus, the left femur was also removed from the matrix. The only major structure missing from the right femur is the intertrochanteric crest. However, the left femur is better preserved overall (Fig 32).

The overall shape of the femur is broadly similar to that of *Didelphis*, although proportionately it is slightly more robust. Unlike the straight femur of *Didelphis*, the bone appears curved in cranial view, with the medial margin convex and the lateral margin concave, especially in the distal two thirds. In lateral view, the proximal epiphysis seems to be positioned slightly more cranially than that of *Didelphis*, and is more similar in this regard to *Caluromys*. When viewed medially, laterally, or proximally, the femoral head is also positioned more cranially relative to the greater trochanter in AÜJM 2002–25 than in *Didelphis*, but is similarly positioned to that of *Caluromys*. The femoral head is more spherical and rounded than that of *Didelphis*, but the anteroposterior dimension of the femoral head is slightly larger than the mediolateral dimension.

On the right specimen, the epiphysial surface surrounding the cranial half of the femoral head is damaged, and this damage extends laterally as far as the contact between the head and the neck. The preservation of the head on the right femur is better, but there is a crack running around the entire perimeter of the contact between the head and the neck, which makes it difficult to interpret. As a result, it is difficult to determine whether or not the proximolateral part of the articular surface extends onto the femoral neck. However, as far as can be judged, the condition in AÜJM 2002–25 seems to be more similar to that of *Dasyurus* and *Sarcophilus*, both of which show little expansion of the articular surface onto the neck compared to *Didelphis* and *Caluromys*. A shallow depression can be seen on the caudomedial quadrant of the head of the right femur, positioned towards the centre. If this is the fovea capitis (for attachment of the ligament of the femoral head), than it is in the practically same spot as it is in *Didelphis*. The head of the left femur is too weathered to provide any unequivocal information regarding the position of the fovea capitis.

In craniocaudal view, the greater trochanter appears roughly the same height as the femoral head, as in most metatherians [49, 119]. On the lateral edge of the greater trochanter, the site of attachment for the M. gluteus medius and M. gluteus profundus is salient and crestlike. In proximal view, the highest point on the greater trochanter is a medially-positioned, salient tubercle, which is probably the origin for the M. piriformis [44]. There is no prominent fossa on the anterior face of the greater trochanter, which houses the M. vastus lateralis [39, 44]. However, there is a wide and shallow depression distomedial to the greater trochanter, which continues a short distance distally.

The lesser trochanter is better developed in AÜJM 2002–25 than in *Didelphis*, and more like the condition in the dasyurids *Dasyurus* and *Sarcophilus*, in which the lesser trochanter extends further proximally towards the femoral head; however, the lesser trochanter extends further medially in *Dasyurus*, to a point close to or level with the medial edge of the femoral head [44]. The lesser trochanter of AÜJM 2002–25 also resembles that of *Sarcophilus* in its degree of distal extension (to approximately 25% of the total length of the bone). AÜJM 2002–25 differs from *Caluromys* in that the lesser trochanter is twice as long proximodistally as it is wide mediolaterally; in *Caluromys* these two dimensions are almost equal [44]. In didelphids, the third trochanter is either missing (e.g. *Didelphis*), or only visible as a faint scar line (e.g. *Caluromys*, *Metachirus*), and it is also uncommon in other marsupials, whereas it is found in several stem-marsupials, including *Herpetotherium* and *Mayulestes* [39, 47, 49, 51, 80]. The third trochanter is a prominent, flaring tubercle in AÜJM 2002–25; it differs from that of *Mayulestes*, which is more obviously continuous with the crestlike lateral border of the greater trochanter [39].

On the caudal side of the proximal femur, the intertrochanteric fossa is shallower than in *Didelphis* and *Caluromys*. However, the proximodistal extension of this fossa is longer than in *Didelphis*, and it extends mediodistally into the concave surface of the flaring lesser trochanter. Just proximal to this opening, there is a salient tubercle, which lies on the elevated surface that forms the medial border of the intertrochanteric fossa.

In medial and lateral views, the femoral shaft appears smooth and straight. However in cranial view, a concavity is noticeable on the lateral side, starting from the level of third trochanter and extending to the lateral condyle. Similarly, the medial margin has a sigmoid shape. The degree of distal curvature somewhat resembles the condition in the dasyurids *Sarcophilus* and *Dasyurus*; however, because dasyurids lack a third trochanter, the degree of curvature does not appear as exaggerated as in AÜJM 2002–25.

Distally, the anterior surface of the bone bears a well-developed trochlea, as in *Herpetotherium* [49], although it is unclear whether a patella was present or not; the patella is absent in most marsupials, but it is present in the stem-marsupial sparassodonts [121]. The medial and lateral crests of the trochlea are similar in height, and the trochlear surface is relatively symmetrical. Neither the medial nor the lateral sides of the trochlea seem to extend proximally onto the diaphysis. The distal portion of the femoral shaft, just proximal to the trochlea, bears a sulcus for the M. vasti and M. rectus femoris tendons [39]. The medial epicondyle is slightly wider than the lateral one, but the lateral epicondyle projects further beyond the edge of the femoral shaft, possibly reflecting contact with the fibula. The medial epicondyle and condyle are deeper proximodistally because they extend slightly more distally than the lateral epicondyle and condyle, as can be seen visible in both cranial and caudal views. In caudal view, the medial and lateral condyles appear similar in width, but the medial condyle is deeper proximodistally, extending further both proximally and distally.

#### Tibia

**Fig 33:**
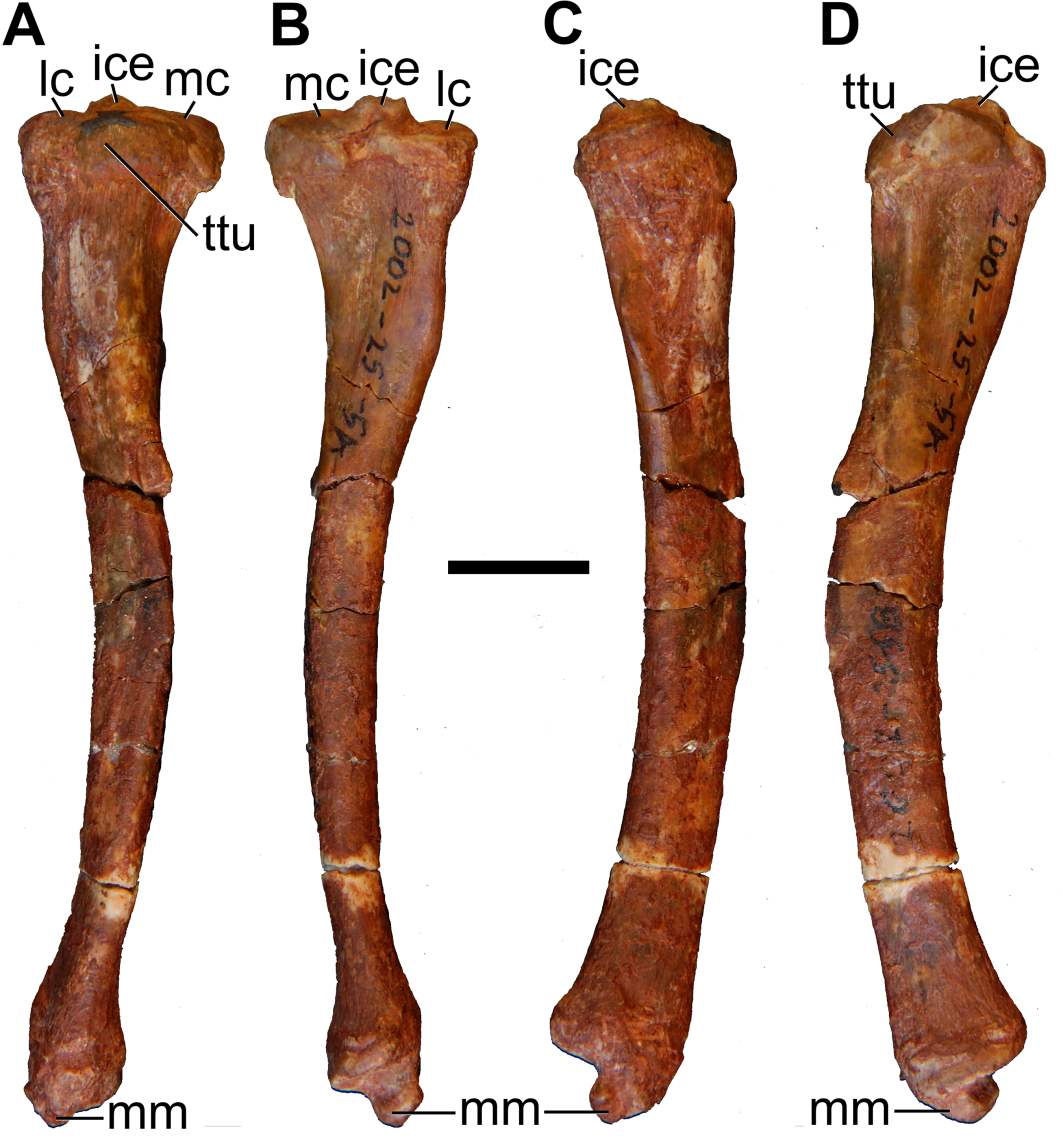
Photographs of left tibia of holotype of *Anatoliadelphys maasae* (AÜJM 2002–25). **A**, cranial (anterior) view; **B**, caudal (posterior) view; **C**, medial view; **D**, lateral view. Abbreviations: ice, intercondylar eminence; lc, lateral condyle; mc, medial condyle; mm, medial malleolus; ttu, tibial tuberosity. Scale bar = 1 cm.

Both tibiae are preserved. Both are complete, but there are some preservational differences between the two. The distal epiphysis of the right specimen was disarticulated when excavated; it was found later in the matrix, but the proximal surface on the epiphysis and the distal surface on the diaphysis were damaged to such an extent that the fit between two fragments is not a good one. The left tibia is broken, but the fits between all the breaks seem to be close, suggesting that relatively little bone has been lost (Fig 33).

Similar to the femur, the tibia of AÜJM 2002–25 is proportionately more robust than that of *Didelphis*. In cranial and caudal views, the tibial shaft is sigmoidal in shape: the medial border of the shaft is initially concave, but this changes further distally, towards the midpoint of the shaft. In medial and lateral views, the tibia is strongly curved craniocaudally, with a concave caudal border and convex cranial border. The right tibia is more curved than the left, especially distally, but no significant deformation or pathology is identifiable on either bone. The left tibia is more broken, and the degree of craniocaudal curvature may have been artefactually reduced when the pieces were reassembled. However, the fit between the preserved pieces appears very close, and so we assume that the left specimen preserves the “true” degree of curvature. However, even when compared to the straighter left tibia of AÜJM 2002–25, most didelphids (with some exceptions, e.g. *Chironectes–*see character 99 of Flores [40]) show a lesser degree of craniocaudal curvature of the tibial shaft; in this regard, AÜJM 2002–25 more closely resembles the stem-marsupials *Mayulestes* and *Pucadelphys* [39, 44].

In proximal view, the craniocaudal length of the proximal epiphysis is relatively short. However, the overall morphology of the proximal epiphysis is broadly similar to that of *Didelphis*. The tibial tuberosity is prominent, and is almost as large as that of *Didelphis*. The lateral condyle has a slightly convex articular surface, which is roughly triangular in shape. The proximal fibular facet occupies the entire caudal border of the lateral condyle. The medial condyle is approximately “reniform” or bean-shaped, and is slightly lower than the lateral condyle. The lateral half of the medial condyle is excavated and deeper than the medial half. The intercondylar eminence is wider than in *Didelphis*.

In cranial view, there is no obvious tibial crest proximally (unlike the stem-metatherian *Herpetotherium* [49]), but ~30–50% of the way from the proximal end, the tibial shaft becomes mediolaterally narrower, and a smooth, rounded ridge is formed that continues distally. The lateral tibial fossa, positioned on the proximal shaft just distal to the epiphysis, is shorter but deeper than that of *Didelphis*, and in this regard is more similar to that of *Dasyurus*. The medial tibial fossa is only identifiable on the right specimen; it differs from the condition in *Didelphis*, in which it is flat. However, this fossa is shallower in AÜJM 2002–25 than in *Caluromys*.

On the posterior side of the proximal tibia, the fossa just distal to the popliteal notch is deeper than in *Didelphis*, resembling *Mayulestes* in this regard. The distal two-thirds of the shaft is mediolaterally compressed. The degree of compression of the distal shaft is broadly comparable to that of *Didelphis* and *Dasyurus*, but in those taxa the cresting forms at the distal third of the bone and becomes increasing well-developed distally.

The facet for the fibular articulation is on the lateral side of the distal epiphysis, and occupies the whole of the lateral margin. With the exception of the distal epiphysis on the right tibia, all of the epiphyses appear fused with the diaphyses. However, as is common in living marsupials [122], the suture lines are usually visible. The distal epiphysis bears the medial malleolus, which is higher and more prominent than in *Didelphis*. The lateral facet for the astragalus is reniform in shape, smooth and has a caudoproximal-to-craniodistal slope. The slope seems to be shallower than that seen in *Didelphis*; however the degree of slope is difficult to judge because the bone itself also bends caudally. In lateral view, the medial astragalotibial facet on the lateral side of the malleolus makes a sharp, almost vertical contact with the lateral astragalar astragalotibial facet. This differs from the condition in *Didelphis*, in which the contact is more gradual, and the angle between the two surfaces is wider. In therians, the distal articular surface of the tibia for the astragalus bears two distinct facets for contact with the astragalus: the medial and the lateral astragalotibial facets. In some metatherians, there is a caudal extension of the lateral astragalotibial facet, referred to as the “posterior astragalotibial facet” [56], which is typically separated from the medial astragalotibial facet by a distinct notch. The lateral astragalotibial facet appears to be slightly extended caudally in AÜJM 2002–25, but much less so than in the posterior astragalotibial facet of some marsupials, and there is no distinct notch between this extension and the medial astragalotibial facet.

#### Fibula

**Fig 34:**
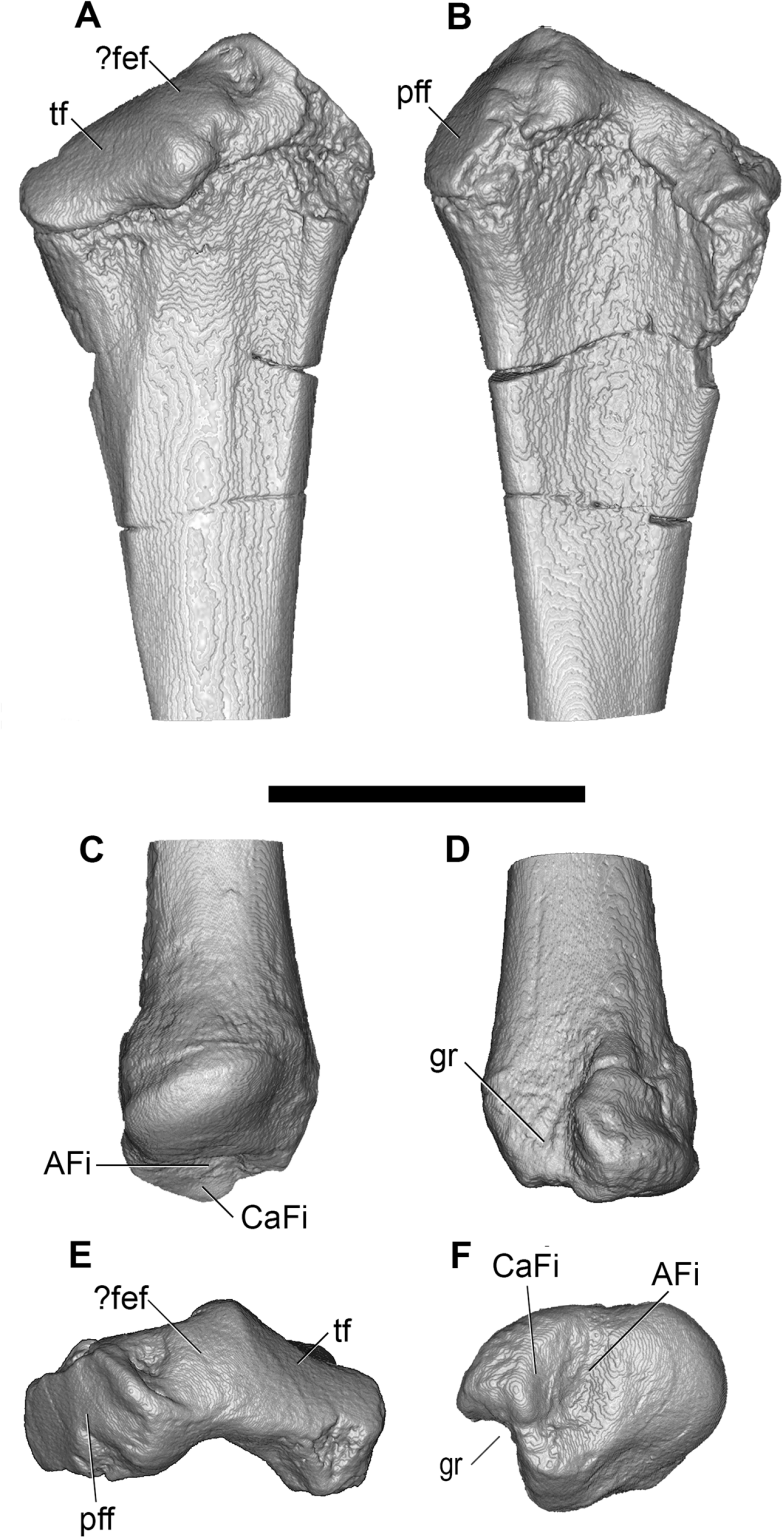
Volume-rendered CT images of right fibula of holotype of *Anatoliadelphys maasae* (AÜJM 2002–25). **A**, medial view of proximal end; **B**, lateral view of proximal end; **C**, medial view of distal end; **D**, lateral view of distal end; **E**, proximal (dorsal) view of proximal end; **F**, distal (ventral) view of distal end. Abbreviations: AFi, astragalofibular facet; CaFi, calcaneofibular facet;? fef,? femoral facet; gr, groove for M. peroneus brevis, M. peroneus longus and M. extensor digitorum lateralis; pff, parafibular (fabellar) facet; tf, tibial facet. Scale bar = 1 cm.

Both left and right fibulae are preserved, but neither is complete (Fig 1). The proximal and distal ends are intact in both fibulae. In the right specimen, the entire shaft is also preserved, but a major break is present approximately one-third the distance from the end of the shaft, and the matching contacts between the proximal and distal parts are minimal (Fig 34). As a result, the full intact morphology of the fibula cannot be ascertained, although the shaft appears relatively straight, as in other metatherians [44].

Overall the fibula is more robust than that of *Didelphis*. In proximal view, the proximal epiphysis is large and laterally flaring as in *Didelphis*, but less than in *Caluromys* [44]. The craniomedial part of this epiphysis bears the proximal tibial facet. In *Didelphis*, this facet is deep, concave, and almost saddle-shaped, and it is even deeper in *Caluromys*. In AÜJM 2002–25, the proximal tibial facet is still concave, but it is shallower than in *Didelphis*. There is a tubercle on the anteromedial side of the flare, just distal to the proximal tibial facet, but this tubercle is smaller than in *Didelphis*. In *Caluromys*, this tubercle is the site of origin of the M. extensor digitorum longus and M. peroneus brevis [44].

When the proximal epiphysis is viewed mediolaterally, the proximalmost point of the parafibular (fabellar) facet is identifiable as a distinct apex. The facet is flat and steep, with its long axis oriented from medioproximal to laterodistal. There is a small groove anteromedial to the apex that separates it from what is probably a facet for contact with the femur (unless it is for the tendon of the M. gastrocnemius externus).

Proximally, the cross-section of the shaft appears mediolaterally compressed, but distally it gradually becomes wider: at a distance of approximately one third of the total shaft length from the proximal end, it is nearly as wide mediolaterally as deep craniocaudally. However, the fibula is deeper craniocaudally than it is wide mediolaterally throughout its entire length. A craniomedial crest originates from the tubercle for the M. peroneus brevis and extends distally as far as the distal 25% of the length of the bone, at which point it becomes smoother and bifurcates. The lateral bifurcation extends as far as the malleolus, whilst the medial bifurcation ends at the distal tibial facet. In life, this crest probably served as an attachment for the interosseus membrane, which was also attached to the crest on the anterolateral side of the tibia.

The shape of the shaft appears to differ somewhat between the left and right fibulae. Distally, the shaft seems to curve slightly caudally on the left side, whereas the shaft of the right fibula appears straight in all views. However, it is not clear which is the real condition, because both bones are fragmented. Some *Didelphis* specimens exhibit a caudal curvature of the distal shaft, but to a lesser extent than in AÜJM 2002–25.

The distal epiphysis bears a groove visible in lateral view, which is probably for the tendons of the M. peroneus brevis, M. peroneus longus and M. extensor digitorum lateralis. This groove is weaker than that seen in *Didelphis*, which is in turn weaker than that in *Caluromys*. Compared to *Didelphis*, the malleolus is smaller in lateral view and it does not seem to extend as far laterally when viewed cranially. The medial side of the distal epiphysis is rounded and articulates with the tibia. In distal view, the astragalofibular facet is roughly triangular in shape. In the same view, there is a deep depression separating the malleolus from the astragalofibular facet. It seems likely that malleolus contacted the calcaneus, and there is also evidence for a calcaneofibular facet on the calcaneus, lateral to (and continuous with) the ectal facet (see below).

#### Calcaneus

**Fig 35:**
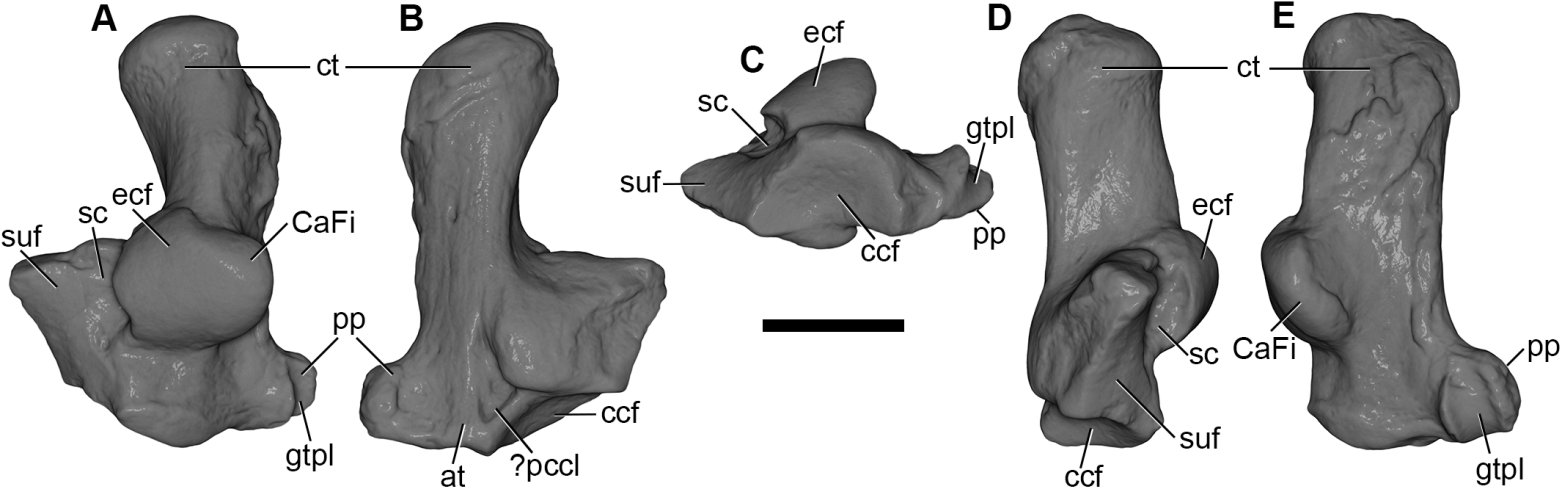
Volume-rendered CT images of left calcaneus of holotype of *Anatoliadelphys maasae* (AÜJM 2002–25). **A**, dorsal view. **B**, ventral (plantar) view. **C**, distal view. **D**, medial view. **E**, lateral view. Abbreviations: at, anterior plantar tubercle; ccf, calcaneocuboid facet; CaFi, calcaneofibular facet; ecf, ectal facet; gtpl, groove for the tendon of M. peroneus longus;? pccl,? pit for plantar calcaneocuboid ligament; pp, peroneal process; sc, sulcus calcanei; suf, sustentacular facet. Scale bar = 1 cm.

Both calcanei are complete and well-preserved (Fig 35). Overall, the calcaneus is relatively robust, especially the calcaneal tuber, which is dorsoventrally very deep. In dorsal view, the tuber is distinctly inflected medially. The ectal (posterior calcaneoastragalar) facet is almost round: its longest axis is anteroposterior, but it is nearly as large mediolaterally and anterolaterally-to-posteromedially. A faint ridge extending anterolaterally from the midpoint of the posterior border of the ectal facet, coinciding with a slight change in facet curvature, probably represents the border between the “true” ectal facet (medially) and a facet for articulation with the fibula (laterally); the morphology of the distal fibula also suggests fibula-calcaneus contact (see above).

The sustentaculum is roughly rectangular in outline. The sustentacular facet extends the entire length of the dorsal surface of the sustentaculum, terminating anteriorly at the medial margin of the calcaneocuboid facet. A wide calcaneal sulcus separates the sustentacular facet from the ectal facet. The sustentacular facet faces somewhat medially at its anterior end, but more dorsally further posteriorly; nevertheless, it seems likely that the degree of superposition of the astragalus on the calcaneus was greater than in the stem-marsupials *Herpetotherium* [49], *Mayulestes* [44] and *Pucadelphys* [47].

There is a distinct raised area at the anterodorsal margin of calcaneus, anterior to the ectal facet and anteromedial to the sustentacular facet. This is unlikely to be a facet for contact with the astragalus, as is (for example) the distal calcaneoastragalar facet seen in dasyuromorphians [56]; this is because the sustentacular facet of AÜJM 2002–25 is large and extends the entire length of the sustentaculum, and the raised area is distinctly medial to the sustentacular facet, whereas the distal calcaneoastragalar facet of dasyuromorphians is immediately anterior to the sustentacular facet. Instead, this raised area is probably for attachment of the anterior (or dorsal) astragalocalcaneal ligament and/or the M. extensor digitorum brevis.

The peroneal process is prominent, but it does not extend anteriorly beyond the level of the calcaneocuboid facet. There is a well-developed groove for the tendon of the M. peroneus longus on the dorsolateral surface of the peroneal process; the medial wall of this groove is particularly prominent and dorsally extensive.

In ventral view, the sustentaculum is triangular in outline. A well-marked groove on the ventral surface of the sustentaculum, towards its medial edge, probably housed the tendons of the M. flexor digitorum fibularis (= M. flexor hallucis longus) and the M. flexor digitorum brevis. Lateral to the anterior part of the sustentaculum, in line with the medial edge of the calcaneal tuber, a raised area represents the anterior plantar tubercle, to which would have attached the plantar calcaneocuboid ligament. Immediately lateral to this, and extending further anteriorly, is a very well-marked pit; this pit clearly did not house a proximal extension of the cuboid like that seen in didelphids [56] because it is not continuous with the calcaneocuboid facet, but rather is enclosed anteromedially by a sharply-defined wall. Instead, it probably represents an additional area of attachment of the calcaneocuboid ligament; a similar fossa or groove has been reported in the sparassodont *Lycopsis* [54] and a few other mammals [123, 124].

In distal view the calcaneocuboid facet is roughly trapezoidal in shape, and is concave. It is not subdivided into distinct facets with different orientations (as seen in some metatherians [49, 51, 56, 125]), but rather forms a single, relatively continuous surface. A distinct groove or fossa immediately lateral to the lateral margin of the calcaneocuboid facet is presumably for the lateral calcaneocuboid ligament.

Compared to other fossil metatherian calcanei known from the early Palaeogene, that of AÜJM 2002–25 shows the greatest overall (phenetic) resemblance to the “Itaborai Metatherian Group” (IMG) III morphotype from the? early Eocene Itaborai fauna of Brazil, and the “Bridger Metatherian Group” (BMG) I morphotype from the middle Eocene Bridger formation of North America, both of which were described by Szalay (56).

#### Metapodials

AÜJM 2002–25 preserves two metapodials (Fig 1). One of these is complete and bears a distinct lateral hook-like projection, allowing it to be confidently identified as the fifth metatarsal. The proximal surface, which is the only feature that allows a metapodial to be identified as either metacarpal or metatarsal, is broken in the second specimen. This specimen is neither the first nor the fifth metatarsal, but no more precise identification can be made.

#### Phalanges

AÜJM 2002–25 preserves four proximal and two intermediate phalanges (Fig 1). Only two of the proximal phalanges are complete: of these, one has a longer and more slender shaft and a sharp distal articular surface, whereas the other shows a more robust and shorter shaft, with a wide distal articular head. The morphology of the former is more consistent with identification as a pedal proximal phalanx, whereas the latter agrees more with the typical morphology of manual phalanges. The remaining two proximal phalanges preserve no diagnostic feature that would allow them to be identified as either manual or pedal. The two intermediate phalanges are also broken, and hence they also cannot be identified as belonging to either the manus or the pes.

### Body mass estimate

Total length of the intact part of the left mandible of AÜJM 2002–25 is 88.2 mm. Using the total jaw length regression equation of Myers [65] for his “dasyuromorphian” dataset and taking into account the “smearing estimate” gives a body mass estimate of 3.37 kg for AÜJM 2002–25, with a range of 2.76–3.97 kg when the percentage error is taken into account. Given that the left mandible is slightly damaged anteriorly, this is undoubtedly a slight underestimate, and so a body mass of 3–4 kg (similar to a typical domestic cat, or a male spotted quoll [*Dasyurus maculatus*]) seems probable.

### Morphofunctional analysis

#### Dentition and cranium

The preserved dentition of AÜJM 2002–25 comprises robust canines, three premolars that increase markedly in size posteriorly (with P3 and p3 large and very broad) and four molars that also increase markedly in size posteriorly. The large, labiolingually broad P3/p3 and the extreme wear of the postcanine dentition are suggestive of durophagy. The exact functional significance of the peculiar, more-or-less continuous wear surface formed by m1, m2 and the trigonid of m3 (more clearly seen on the left mandible) is unclear, but presumably reflects the consumption of particularly abrasive food items.

In general, hypercarnivorous mammals are characterised by a suite of characteristic molar features [66]. On the upper molars, the postmetacrista is elongate and often has a carnassial notch, and the paracone is reduced, while on the lower molars the paraconid is tall, the paracristid is tall and blade-like and often has a carnassial notch, and the metaconid is reduced; collectively, the morphology of the upper and lower molars reflects an increased emphasis on postvallum-prevallid shear. In addition, the protocone of the upper molars and talonid of the lower molars are also usually reduced, reflecting reduced emphasis on crushing. *Anatoliadelphys* exhibits only some of these features (Figs 2–5 and 12–19): the postmetacrista and paracristid are relatively elongate (particularly on M3 and m4 respectively), but neither crest has a distinct carnassial notch, and the paraconid is very low (rather than tall) on the lower molars. The protocone is also prominent and anteroposteriorly broad, and the talonid with which it occludes is similarly well-developed, reflecting a well-developed capacity for crushing. The latter may reflect durophagy or, alternatively, a meso- or hypo-carnivorous (rather than hypercarnivorous) diet.

The cingula that extend along the anterior and posterior margins of the upper molars are distinctive features of *Anatoliadelphys*. It has often been suggested that the function of dental cingula is to prevent damage to the gingiva from impacted food items and/or the cusps of occluding teeth [126–129]. However, a recent study using finite element analysis of simplified “virtual” tooth models concluded that dental cingula may function to reduce tensile strains in the enamel caused by forces generated when consuming soft foods, with partial cingula particularly effective at reducing strains generated by asymmetrical loads [130]. Regardless, the functional significance of the dental cingula of *Anatoliadelphys* is not obvious, for several reasons. Firstly, whilst the upper molar cingula are relatively well-developed in *Anatoliadelphys*, the anterior cingulid (precingulid) and posterior cingulid (postcingulid) on the lower molars are extremely weakly developed or absent. Secondly, the presence of such upper molar cingula is rare within metatherians (see character 50 of Williamson et al. [28, 131]). Thirdly, although the study of Anderson et al. [130] found that a dental cingulum is of greater functional importance under “soft-food” forces than under “hard-food” forces, the craniodental morphology of *Anatoliadelphys* strongly suggests some degree of durophagy.

The five dental indices of Zimicz [67] for *Anatoliadelphys* and a range of other faunivorous metatherians are shown in Table 3. When considered individually, these indices support different interpretations for the diet of *Anatoliadelphys*. Following the critical values proposed by Zimicz [67], a Relative Grinding Area index (RGA) of 0.65 suggests mesocarnivory, whilst Premolar Shape (PS) and Relative Premolar Size (RPS) indices of 0.70 and 2.89 respectively are congruent with its being a bonecracker or other durophage. A Relative Premolar Length index of 0.75 suggests hypercarnivory, whilst a Relative Blade Length (RBL) index of 0.61 indicates hypocarnivory.

**Table 3:** Body masses and morphofunctional dental indices (from Zimicz [67]) for *Anatoliadelphys* and other carnivorous metatherians weighing <10 kg. RGA (Relative Grinding Area) = square root of the area of the talonid of m4/length of m4; RPS (Relative Premolar Size) = width of the largest lower premolar/cube root of body mass in kg; PS (Premolar Shape) = width of the largest lower premolar/length of largest lower premolar; RPL (Relative Premolar Length) = length of largest lower premolar/length of m4; RBL (Relative Blade Length) = length of the trigonid of m4/total length of m4. The dagger symbol (†) indicates fossil taxa.

Species	Clade	body mass (kg)	RGA	RPS	PS	RPL	RBL	Source of measurements (specimen numbers)
**†*Anatoliadelphys maasae***	**†Anatoliadelphidae**	**3.5**	**0.65**	**2.89**	**0.70**	**0.75**	**0.61**	**this study (AÜJM 2002–25)**
*Dasyurus geoffroii*	Dasyuridae	1.107	0.34	1.67	0.49	0.64	0.74	this study (AM M1427, M1541, M10370, P756 and P757)
*Dasyurus hallucatus*	Dasyuridae	0.471	0.47	1.79	0.46	0.74	0.65	this study (AM M5044, M8673, M9081, M21230, M22902 and M26350)
*Dasyurus maculatus*	Dasyuridae	3.284	0.41	1.56	0.51	0.64	0.71	this study (AM M1666, M4330, M4720, M6748, M7388, M7399 and S2124)
*Dasyurus spartacus*	Dasyuridae	0.886	0.38	2.08	0.53	0.70	0.71	this study (AM M37432)
*Dasyurus viverrinus*	Dasyuridae	1.101	0.44	1.65	0.45	0.76	0.69	this study (AM M3776, M5269, M6525, M6604 and M7389)
*Phascogale tapoatafa*	Dasyuridae	0.193	0.54	2.57	0.65	0.80	0.64	this study (AM M35626, M35919, M37467, M37468 and M37469)
*Sarcophilus harrisii*	Dasyuridae	8.202	0.00	2.78	0.80	0.62	1.00	this study (AM M23599 and M44955)
†*Lotheridium mengi*	†Deltatheroida	0.479	0.76	2.21	0.52	0.94	0.57	[100]
†*Sulestes karakshi*	†Deltatheroida	0.108	0.42	2.52	0.50	1.06	0.71	[132]
*Chironectes minimus*	Didelphidae	0.974	0.72	2.00	0.49	0.81	0.65	[67, 133]
*Didelphis albiventris*	Didelphidae	0.9	0.61	1.30	0.21	1.10	0.61	[67]
*Didelphis aurita*	Didelphidae	1.16	0.63	1.42	0.22	1.10	0.60	[67]
*Didelphis marsupialis*	Didelphidae	1.1	0.65	1.45	0.26	0.93	0.53	[67]
*Lutreolina crassicaudata*	Didelphidae	0.555	0.57	2.07	0.46	0.86	0.75	[67, 134]
*Philander opossum*	Didelphidae	0.75	0.55	1.25	0.31	0.88	0.62	[67]
†*Thylatheridium cristatum*	Didelphidae	0.2	0.55	2.28	0.62	0.60	0.68	[67, 135]
†*Thylophorops chapadmalensis*	Didelphidae	3.7	0.89	1.55	0.38	0.75	0.57	[67, 135]
†*Galadi speciosus*	Peramelemorphia	0.93	0.41	1.65	0.57	0.78	0.46	[136]
†*Hesperocynus dolgopolae*	†Sparassocynidae	0.3	0.28	2.24	0.58	0.68	0.74	[137]
†*Sparassocynus bahiai*	†Sparassocynidae	0.37	0.50	2.37	0.57	0.69	0.69	[137]
†*Sparassocynus derivatus*	†Sparassocynidae	0.36	0.49	2.21	0.58	0.65	0.70	[137]
†*Acyon herrerae*	†Sparassodonta	7.84	0.30	1.69	0.34	0.93	0.78	[67, 138]
†*Borhyaenidium musteloides*	†Sparassodonta	1.56	0.30	1.98	0.41	0.92	0.83	[67, 138]
†*Cladosictis centralis*	†Sparassodonta	3.4	0.17	1.90	0.44	0.83	0.75	[67, 138]
†*Cladosictis patagonica*	†Sparassodonta	4.68	0.17	1.93	0.44	0.83	0.78	[67, 138]
†*Pseudonotictis pusillus*	†Sparassodonta	1.2	0.30	1.54	0.38	0.86	0.90	[67, 138]
†*Sipalocyon gracilis*	†Sparassodonta	3.15	0.33	1.60	0.40	0.88	0.85	[67, 138]
†*Didelphodon vorax*	†Stagodontidae	1.728	0.53	4.35	0.76	0.96	0.54	[29, 139, 140]
†*Eodelphis browni*	†Stagodontidae	0.653	0.78	3.04	0.59	0.83	0.57	[141, 142]
†*Badjcinus turnbulli*	Thylacinidae	3.059	0.39	1.10	0.38	0.68	0.65	[143]
†*Mutpuracinus archibaldi*	?Thylacinidae	3.197	0.35	1.56	0.49	0.81	0.64	[144]

**Fig 36:**
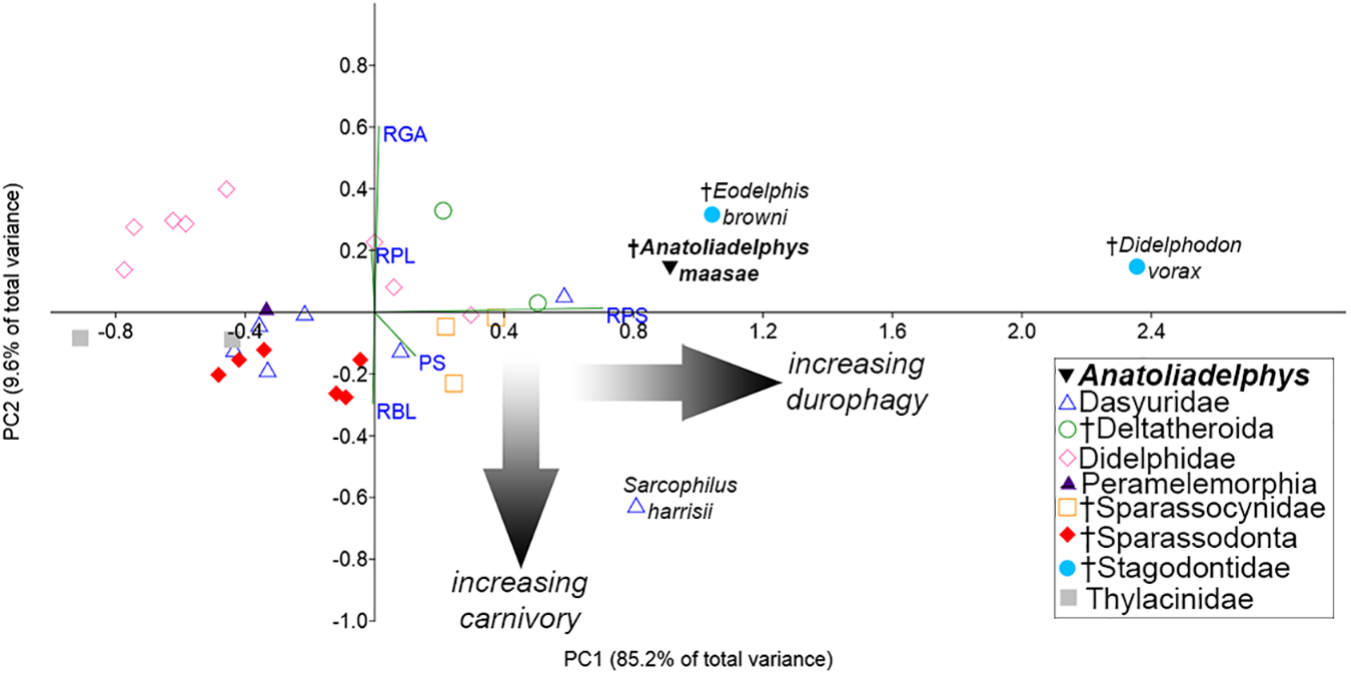
Principal component analysis of five morphofunctional dental indices for *Anatoliadelphys maasae* and other carnivorously-adapted metatherians. The morphofunctional dental indices used were Relative Grinding Area (RGA), Relative Premolar Size (RPS), Premolar Size (PS), Relative Premolar Length (RPL) and Relative Blade Length (RBL). The plot shows the first two principal components (PCs) only.

A principal component analysis of all five dental indices for *Anatoliadelphys* and a wide range of fossil and modern metatherians (Fig 36) places *Anatoliadelphys* closest to the fossil stagodontids *Eodelphis* and *Didelphodon*, with which it shares high values for Principal Component (PC) 1. Higher values of PC1 indicate a larger Relative Premolar Size (RPS) index, and so plausibly represent increasing durophagy. Numerous papers have proposed that stagodontids were specialised durophages [29, 75, 101, 145–147], and it is striking that *Anatoliadelphys*, *Eodelphis* and *Didelphodon* all show greater PC1 and RPS values than the living bone-cracker *Sarcophilus*; however, it should be noted that they considerably smaller in terms of body mass than *Sarcophilus*, and it seems plausible that RPS does not need to be as large for effective processing of hard food items in larger-bodied taxa. A possible example of this is the late Oligocene sparassodont *Australohyaena antiqua*, which has an estimated body mass of approximately 70 kg (i.e. nearly ten times larger than that of *Sarcophilus*), and has an RPS of 2.32, which is less than the critical value proposed by Zimicz [67, 68] as indicating durophagy (RPS ≥ 2.6), even though its skull shows numerous probable bone-cracking specialisations [148].

Using the method of Therrien [76], as modified for metatherians by Wilson et al. [77], estimated dorsoventral bending force (Zx/L) at c1 for the ~3-4kg *Anatoliadelphys maasae* (Zx/L = 0.013) is similar to that for the extant didelphid *Didelphis virginiana* (Zx/L = 0.015), which has an average mass of ~2.5 kg [70]. However, this is considerably less than in the Late Cretaceous stagodontid *Didelphodon vorax* (Zx/L = 0.023), which weighed up to 5.2 kg [75], or the much larger (~8.2 kg [70]) extant dasyurid *Sarcophilus harrisii* (Zx/L = 0.074; Table 4). The mandible of *A*. *maasae* is predicted as being capable of being able to resist high forces more posteriorly, largely due to the dorsoventrally deep mandibular body, particularly in the region of m3 and m4; estimated dorsoventral bending force immediately posterior to m4 is ~40% greater in *A*. *maasae* (Zx/L = 0.062) than in *D*. *vorax* (Zx/L = 0.044), and nearly twice that of *D*. *virginiana* (Zx/L = 0.035). Estimates of bite force in *A*. *maasae* will require further testing, for example via finite element analysis of the mandible [149], or by discovery of more complete cranial remains that might allow calculation of bite force using the “dry skull method” [72, 73]; nevertheless, its mandibular morphology clearly indicates that it was capable of generating high bite forces relative to body size, particularly towards the back of the jaw.

**Table 4:** Measurements and calculations of mandibular bending strength for *Anatoliadelphys maasae*, *Didelphodon vorax*, *Sarcophilus harrisii* and *Didelphis virginiana*, based on the methods of Therrien [74], as modified for metatherians by Wilson et al. [75]. Values for *Anatoliadelphys maasae* are from this study; values for the other taxa are taken from Wilson et al. [75]. Ix = second moment of area about the labiolingual axis; Zx = bending strength in the dorsoventral plane; Iy = second moment of area about the dorsoventral axis; Zy = bending strength in the labiolingual plane.

Taxon	Tooth position	Distance to condyle (L)	Dorsoventral radius (a)	Labiolingual radius (b)	Ix	Zx	Dorsoventral bending force (Zx/L)	log_10_ (Zx/L)	Iy	Zy	Labiolingual bending force (Zy/L)	log_10_ (Zy/L)	Relative force (Zx/Zy)
*Anatoliadelphys maasae* (AÜJM 2002–25)	c1	7.97	0.49	0.57	0.05	0.11	0.013	-1.87	0.07	0.13	0.016	-1.80	0.86
	p2-p3	7.28	0.61	0.37	0.06	0.10	0.014	-1.84	0.02	0.06	0.0087	-2.06	1.66
	p3-m1	6.62	0.66	0.33	0.07	0.11	0.017	-1.77	0.02	0.06	0.0085	-2.07	1.98
	m1-m2	6.13	0.75	0.33	0.11	0.14	0.023	-1.63	0.02	0.06	0.010	-1.98	2.26
	m2-m3	5.56	0.82	0.34	0.14	0.17	0.031	-1.50	0.02	0.07	0.013	-1.89	2.43
	post-m4	4.1	0.91	0.40	0.23	0.25	0.062	-1.21	0.04	0.11	0.027	-1.57	2.29
*Didelphodon vorax* (UWBM 102139)	c1	8.08	0.6	0.66	0.11	0.18	0.023	-1.64	0.13	0.2	0.025	-1.6	0.91
	p2-p3	7.58	0.66	0.5	0.12	0.17	0.023	-1.64	0.07	0.13	0.017	-1.76	1.32
	p3-m1	7	0.73	0.48	0.15	0.2	0.029	-1.54	0.06	0.13	0.019	-1.72	1.52
	m1-m2	6.51	0.78	0.44	0.16	0.21	0.032	-1.5	0.05	0.12	0.018	-1.75	1.78
	m2-m3	6	0.84	0.44	0.21	0.25	0.041	-1.39	0.06	0.13	0.021	-1.67	1.93
	post-m4	4.68	0.76	0.45	0.15	0.2	0.044	-1.36	0.06	0.12	0.026	-1.58	1.67
*Didelphodon vorax* (UCMP 159909)	c1	–	0.82	0.59	0.26	0.31		–	0.13	0.22		–	1.39
	p2-p3	–	0.87	0.56	0.29	0.33		–	0.12	0.21		–	1.56
	p3-m1	–	0.89	0.55	0.3	0.34		–	0.11	0.21		–	1.63
	m1-m2	–	0.88	0.47	0.25	0.28		–	0.07	0.15		–	1.87
	m2-m3	–	0.91	0.45	0.27	0.29		–	0.07	0.15		–	2
*Sarcophilus harrisii* (UWBM 20671)	c1	9.51	0.96	0.97	0.67	0.7	0.074	-1.13	0.68	0.7	0.074	-1.13	0.99
	p2-p3	8.42	1.02	0.47	0.4	0.39	0.047	-1.33	0.09	0.18	0.021	-1.67	2.16
	p3-m1	7.57	1.06	0.44	0.41	0.39	0.051	-1.29	0.07	0.16	0.021	-1.67	2.42
	m1-m2	6.57	1.03	0.48	0.41	0.4	0.060	-1.22	0.09	0.18	0.028	-1.56	2.17
	m2-m3	5.48	1.03	0.57	0.5	0.48	0.087	-1.06	0.15	0.27	0.049	-1.31	1.8
	post-m4	4.13	1.2	0.53	0.72	0.6	0.14	-0.84	0.14	0.26	0.065	-1.19	2.28
*Didelphis virginiana* (UWBM 12555)	c1	9.27	0.57	0.54	0.08	0.14	0.015	-1.82	0.07	0.13	0.014	-1.85	1.06
	p2-p3	7.83	0.72	0.34	0.1	0.14	0.018	-1.75	0.02	0.07	0.0083	-2.08	2.13
	p3-m1	7.04	0.73	0.33	0.1	0.14	0.019	-1.71	0.02	0.06	0.0089	-2.05	2.19
	m1-m2	6.55	0.75	0.3	0.1	0.13	0.019	-1.71	0.02	0.05	0.0078	-2.11	2.52
	m2-m3	5.95	0.74	0.3	0.09	0.13	0.021	-1.67	0.01	0.05	0.0085	-2.07	2.5
	post-m4	4.6	0.82	0.31	0.13	0.16	0.035	-1.45	0.02	0.06	0.013	-1.88	2.68

*Sarcophilus harrisii* is the smallest bone-cracking mammal alive today, weighing ~8kg, ~2-3x larger than *Anatoliadelphys*. It is unclear whether there is a lower limit on body size for bone-cracking to be a feasible dietary strategy. Nevertheless, the comparatively small size of *Anatoliadelphys* relative to known bone-cracking mammals may be an indication that it was not a bone-cracker, but instead consumed other hard food items such as hard-shelled invertebrates (as has been proposed for the enigmatic fossil marsupial *Malleodectes*) [150, 151].

The prominent sagittal crest of *Anatoliadelphys*, probably approximately similar in size to that of *Didelphis*, indicates that the temporal musculature was well-developed. The area of origin of the deep masseter on the ventrolateral surface of the zygomatic process and the posterior shelf of the masseteric fossa are also prominent, suggesting that the masseter muscles were also well-developed. Collectively, the powerfully-developed jaw muscles suggest a high bite force, congruent with the large, very broad P3 and p3, which presumably had a crushing function.

A prominent nuchal crest, as seen in *Anatoliadelphys*, is usually interpreted as indicating strongly-developed neck musculature, indicative of powerful dorsoventral and lateral movements of the head and neck [45]. In turn, this has been argued to be suggestive of predatory behaviour; *Didelphis*, which shows a similar degree of development of the nuchal crest to that seen in *Anatoliadelphys*, subdues prey items by violently shaking them from side to side [152, 153], as do dasyurids [154, 155]. However, it may instead simply reflect the presence of a large head relative to body size, which requires well-developed neck muscles to support it.

In summary, the known craniodental morphology of *Anatoliadelphys* suggests that this animal was specialised for durophagy (based on its large, very broad P3 and p3), and was capable of generating high bite forces (based on its robust mandible and prominent jaw muscle attachments). However, it was probably not a hypercarnivore, but instead had a more generalised, meso- or hypo-carnivorous diet, based on the absence of a very elongate, notched postmetacrista and tall, notched paracristid, and the presence of a robust protocone and well-developed talonid.

#### Postcranium

The glenoid of the scapula of *Anatoliadelphys* is a cranially-elongated oval (“pear”) shape, as is typical of most mammals, with the exception of cursorially-specialised forms [43]. The rounded (rather than transversely compressed) humeral head that is slightly higher than the greater tubercle suggests considerable mobility of the shoulder, congruent with some degree of climbing ability [39, 43, 48]. The humeral head protrudes posteriorly further than in the aboreal didelphid *Caluromys*, which might indicate a lesser range of movement at the scapulo-humeral joint [43], but the articular surface faces more posteriorly than proximally, and the overall morphology is broadly similar to the condition in other didelphids and the stem-marsupials *Pucadelphys* and *Mayulestes* [43]. The well-developed teres tuberosity indicates a powerful M. teres major, which is a flexor of the shoulder; the teres tuberosity is large in both arboreal and semi-fossorial therian mammals [58], but is also associated with a relatively abducted forelimb position, which is likely plesiomorphic for therians. The deltopectoral crest is prominent, particularly at its distal end, but extends distally only to about the mid-point of the humerus (deltopectoral crest length/total humeral length = 0.47). A short but strongly-developed deltopectoral crest is characteristic of some arboreal didelphids (e.g. *Caluromys*, *Glironia*; [41, 43]), where it may reflect powerful grasping, but it is also seen in some semi-fossorial taxa, e.g. the sciurids *Cynomys* and *Marmota* [58].

The radial and olecranon fossae are deeper than in the arboreal didelphid *Caluromys*, and the olecranon fossa is slightly deeper than in the more scansorial *Didelphis*, but much shallower than in *Dasyurus* and *Sarcophilus* [43, 52]. The crests of the humeral trochlea are not particularly well-developed, although somewhat more prominent than in arboreal didelphids *Caluromys* and *Glironia* [41, 43], and the articular surface of the trochlea is not extensive proximally either cranially or caudally. Together with the relatively shallow trochlear notch and only moderately prominent anconeal process of the ulna, this indicates that the elbow joint of *Anatoliadelphys* was more stabilised than in arboreally-specialised marsupials, but less so than in the more terrestrially-adapted dasyurids and didelphid *Metachirus*, or the stem-marsupial *Mayulestes*. This suggests that *Anatoliadelphys* was capable of climbing, but that it did not have particularly agile, rapid movements.

The relatively spherical capitulum would allow considerable pronation and supination of the forearm. The entepicondyle is well-developed and protrudes medially, as in all didelphids except *Metachirus* [43, 48], indicating the presence of powerful flexors of the manus and digits; unlike the condition in the stem-marsupial *Mayulestes*, it does not protrude distomedially [39]. Although broken, the supinator crest is also prominent, suggesting that the extensors of the manus and digits, and the supinator of the forearm, were also well-developed.

The olecranon process of the ulna appears longer than in dasyurids and arboreal didelphids [43, 52]; proportionately, the relative size of the olecranon process appears similar to that of the terrestrial didelphid *Monodelphis* and the stem-marsupial *Mayulestes* [39, 43]. The olecranon process is not as long proportionately as in the semi-fossorial bandicoots and bilby [156] or in other semi-fossorial and fossorial mammals [76, 157], and nor is it obviously medially inflected, suggesting that *Anatoliadelphys* was not specialised for digging. The caudal margin of the olecranon is not inclined cranially towards its proximal end, whereas a strong incline is seen in arboreal didelphids and several other arboreal mammals [43, 48, 58]. The anconeal process is more prominent and the trochlear notch is deeper than in arboreal didelphids, but less than in the terrestrially-specialised *Metachirus* [43].

The radial head is rounded, similar in shape to those of *Didelphis* and *Caluromys*, whilst the facet for the humeral capitulum is deeper than that of *Didelphis*. This, together with the spherical capitulum, indicates considerable capacity for pronation and supination, which is suggestive of locomotion over uneven substrate, and probably also the capacity to climb [40, 47, 48]. This interpretation is supported by the very prominent bicipital tuberosity, and the strongly curved radial shaft (similar in morphology to that seen in the didelphid *Monodelphis* and the stem-marsupial *Mayulestes* [43]); the latter feature would have provided space for a very large M. flexor digitorum profundus [43].

The most striking feature of the os coxae (innominate bone) of *Anatoliadelphys* is the laterally everted ilium, which would have provided a larger insertion area for the M. longissimus dorsi (the major extensor of the back) and more space for the gluteal muscles [39, 44, 48, 58]. The ilia of most didelphids are relatively straight, but lateral eversion of the ilium is seen in the agile *Metachirus*, dasyurids and *Mayulestes* [39, 44, 48]. The presence of a laterally everted ilium, indicating more powerful epaxial musculature, has often been interpreted as evidence of greater agility, possibly including leaping [39, 44, 48, 58]. However, several non-agile mammals, such as the koala, wombat, bears and anteater, also exhibit a laterally everted ilium [39, 58], reflecting changes in the orientation of the lever-arm of the gluteal muscles [158]. *Anatoliadelphys* differs from agile mammals such as *Metachirus* in that the gluteal fossa does not appear to be much larger than the iliacus fossa, and the greater trochanter of the femur does not extend further proximally than the femoral head (see below). This, together with the general robustness of its postcranial skeleton, suggests that *Anatoliadelphys* was not a highly agile, leaping animal. The ischiatic spine is the origin of the M. biceps femoris, the M. semimembranosus, and the M. semitendinosus caput ventrale, all of which are powerful extensors of the hip [44, 48]; in agile and rapidly-moving (e.g. leaping, bounding or running) mammals, the ischiatic spine is usually prominent and everted [48], but it appears only very weakly developed and not obviously everted in *Anatoliadelphys*. A laterally everted ilium has also been associated with bipedalism [58], and it is possible that *Anatoliadelphys* was capable of periods of bipedal locomotion, perhaps in the context of climbing or scanning the environment.

A distinct tuberosity for the M. rectus femoris, anterior to the acetabulum, is present in *Anatoliadelphys*, as it is in dasyurids and in the stem-marsupials *Herpetotherium*, *Pucadelphys* and *Mayulestes* [39, 48–50]. In didelphids, this tuberosity is absent or weakly developed in slow-moving arboreal forms such as *Caluromys* and *Micoureus*, but better developed in the agile *Metachirus*, which resembles *Dasyurus* in this regard [48]. The M. rectus femoris is a powerful extensor of the knee and flexor of the hip, and presence of a well-developed tuberosity has been interpreted as evidence of agility [39, 48, 49]. It might be an indication that *Anatoliadelphys* was capable of periods of relatively rapid locomotion, perhaps in pursuit of prey.

In the femur, the greater trochanter (the area of insertion of the gluteal muscles) extending proximally above the height of the femoral head is characteristic of agile taxa such as *Metachirus* and the bandicoot *Perameles* [48], and this feature is often associated with a much larger gluteal fossa than iliacus fossa [44, 48]. However, the greater trochanter is similar in height to the femoral head in *Anatoliadelphys* and the gluteal fossa does not appear greatly enlarged, and so it was probably not as agile as *Metachirus* or *Perameles*. The lesser trochanter is prominent and bladelike, and distally more extensive than in didelphids or *Mayulestes*, resembling dasyurids in this respect [39, 44, 52], which suggests that the M. iliacus and M. psoas major (which are flexors and external rotators and adductors of the leg [48]) were well-developed; a large lesser trochanter may be an indication of climbing ability [44, 48]. The apex of the lesser trochanter is positioned close to the femoral head, as in *Dasyurus* and *Metachirus*, suggesting rapid flexion [44]. In medial view, the lesser trochanter extends largely medially and only slightly caudally, as is typical for more scansorial/arboreal didelphids [41], although the exact functional significance of the orientation of the lesser trochanter is somewhat unclear [58].

Presence of a distinct third trochanter (which receives the insertion of the M. gluteus superficialis, an extensor and abductor of the femur) is probably plesiomorphic for Metatheria [47] but is absent in most crown-clade marsupials, with the exception of paucituberculatans, vombatids and notoryctids [47, 51, 80]. The third trochanter of *Anatoliadelphys* is laterally more extensive in *Anatoliadelphys* than in *Mayulestes*, *Pucadelphys* and paucituberculatans [39, 51, 159], extending beyond the lateral margin of the greater trochanter, but is similar in this regard to that of *Herpetotherium* [49, 50]. It is also relatively distally positioned, with its apex ~30% of the total length of the femur from the proximal end, broadly similar to its position in *Pucadelphys*, but further distal than in *Mayulestes* [39], *Hereptotherium* [49, 50] and paucituberculatans [51, 159]. A distal position of the third trochanter may indicate that the M. gluteus superficialis was acting more as an abductor than as an extensor [62]. A more distal location and greater degree of lateral extension of the third trochanter has been interpreted as evidence of greater terrestrial locomotion in tupaiid treeshrews [160], but evidence from other mammalian clades is more equivocal [62, 161]. Nevertheless, the third trochanter of *Anatoliadelphys* appears less prominent than in fossorially-specialised mammals [80, 161].

The crests of the femoral trochlea are more salient in *Anatoliadelphys* than in didelphids, including the relatively terrestrial *Metachirus*, but similar in this regard to *Dasyurus*, suggesting relatively rapid and powerful movements of the knee joint (the trochlear crests prevent dislocation of the tendon of the M. vasti and M. rectus femoris); however, the trochlea is not as deeply defined as the agile, terrestrial *Perameles* [39, 44].

In caudal view, although the medial condyle extends further proximally and distally than does the lateral condyle, the two condyles are of similar widths, with a ratio of medial condyle width to lateral condyle width of 0.90. This is greater than that of any didelphid measured by Muizon [39] or Argot [44], including the more terrestrial *Monodelphis* (0.83) and *Metachirus* (0.75), and also greater than that of *Dasyurus* (0.78), *Mayulestes* (0.73) and *Pucadelphys* (0.75–0.82). The terrestrial caenolestids and *Perameles* have condyles of roughly equal width [44, 47, 159], as in *Anatoliadelphys*, whilst the medial condyle is wider than the lateral condyle in the borhyaenoid *Borhyaena*, which has been interpreted to be terrestrial and digitigrade [53]. A lateral condyle that is wider than the medial condyle width is suggestive of a very abducted position for the femur, which may be plesiomorphic for mammals [39]; in therians, it may be an indication of arboreality [39, 44, 47, 48]. The relatively equal widths of the condyles in *Anatoliadelphys* suggests a less abducted femur, possibly indicative of more rapid, terrestrial locomotion.

The tendon of the M. quadriceps (a powerful extensor of the knee) inserts on the tibial tuberosity. In didelphids, the development of the tibial tuberosity is correlated with the degree of terrestriality [44, 48]. The tibial tuberosity of *Anatoliadelphys* is more prominent than that of the arboreal *Caluromys*, similar in size to that of the scansorial *Didelphis*, and less developed than that of the agile, terrestrial *Metachirus*. The tibial crest of *Anatoliadelphys* is also not as sharp as in *Metachirus*, again suggesting a lesser degree of agility [44, 48]. The sigmoidal shape of the tibia in craniocaudal view is likely plesiomorphic for therians and may be further indication of climbing ability [47, 48]; however, the tibia is sigmoidal in the relatively terrestrial *Caenolestes* and *Sarcophilus* and straight in the arboreal diprotodontians *Phalanger* and *Phascolarctos* [39], and so by itself this feature is of limited functional significance. The tibia of *Anatoliadelphys* is more strongly curved in mediolateral view (convex anteriorly, concave posteriorly) than in didelphids or dasyurids, but resembles *Pucadelphys* and *Mayulestes* in this regard; this would probably have resulted in a large space between the tibia and fibula, which may have been filled by a particularly well-developed M. flexor digitorum fibularis [44]. The medial tibial condyle is slightly lower than the lateral condyle, which helps to stabilise the knee joint; in didelphids, the difference in height is greater in terrestrial forms (e.g. *Metachirus*) than in arboreal forms (e.g. *Caluromys*) [44].

The large, flared proximal end of the fibula of *Anatoliadelphys* may be plesiomorphic for therians [47], and it suggests that the M. flexor fibularis and (particularly) M. peroneus longus were well-developed [44]. The proximal end of the fibula of *Anatoliaelphys* is smaller than that of *Caluromys*, but similar in size to that of *Didelphis* and larger than that of *Metachirus* (in which the hallux is still opposable) and dasyurids (in which the hallux is reduced and non-opposable) [44, 48]. However, the groove at the distal end of the fibula for the M. peroneus brevis, M. peroneus longus and M. extensor digitorum lateralis is less prominent than in *Didelphis* and much less so in *Caluromys* [44], suggesting a less powerful grasping ability. The pes is insufficiently preserved in AÜJM 2002–25 to reveal whether or not the hallux was opposable.

On the distal epiphysis of the tibia of *Anatoliadelphys*, the medial and lateral astragalotibial facets meet at a sharper angle than in didelphids, including the terrestrially-adapted *Metachirus* [56]. This suggests that the upper ankle joint of *Anatoliadelphys* was less mobile than in didelphids, congruent with more terrestrial locomotion. However, neither the left nor the right astragalus is preserved in AÜJM 2002–25, and so the degree of angulation between the astraglotibial and astragalofibular facets of the astragalus is unknown. Conversely, the short, deep, medially-inflected calcaneal tuber, and the prominent peroneal process with a very well-defined groove for the tendon of the M. peroneus longus, indicate that the hindfoot of *Anatoliadelphys* was capable of powerful grasping and inversion-eversion [39, 44, 56, 162, 163]. The ectal facet of *Anatoliadelphys* appears larger than the sustentacular facet, it has a prominent lateral border proximally, and it faces more medially than in the more terrestrial *Metachirus*, dasyurids and peramelids, but more dorsally than in the arboreal *Caluromys* [44, 48, 56].

A small calcaneofibular facet appears to be present in *Anatoliadelphys*; among marsupials, contact between the fibula and calcaneus probably helps stabilise the upper ankle joint, and it is generally found among more terrestrially-adapted taxa [56]. However, calcaneofibular contact is plesiomorphic for therian mammals [56], and so its apparent presence in *Anatoliadelphys* may be of limited functional significance. The broad, concave calcaneocuboid facet of *Anatoliadelphys* would have allowed considerable rotation of the pes at the transverse tarsal joint [44, 56], which may have facilitated climbing. However, it lacks the morphological specialisations of the calcaneocuboid facet to facilitate rotation at the transverse tarsal joint seen in didelphids (in which the calcanceocuboid facet is bipartite, to accommodate a distinct proximal extension of the cuboid) and plesiomorphic australidelphians (in which the facet is tripartite) [56, 125, 164].

In summary, there are features of the forelimb of *Anatoliadelphys* that would have facilitated pronation-supination movements (e.g. the spherical capitulum) and powerful grasping (e.g. the prominent medial epicondyle and supinator crest), suggesting an ability to climb (although it should be noted that these features are probably plesiomorphic for therians); however, they are less developed in *Anatoliadelphys* than in arboreal specialists such as the didelphid *Caluromys*. Some features of the hindlimb are also indicative of powerful grasping and inversion-eversion (e.g. the large peroneal process of the calcaneus with its very prominent groove for the tendon of the M. peroneus longus), but others (such as the laterally flaring ilium, well-developed tubercle for the M. rectus femoris, well-defined femoral trochlea, and femoral condyles that are of similar width) are typical of more agile, more terrestrial marsupials. Thus, we suggest that *Anatoliadelphys* was not an arboreal specialist, but was nevertheless capable of climbing. It may have had similar scansorial abilities to *Didelphis*, but was probably capable of more rapid, agile movements. In this regard, it may have resembled the living spotted quoll *Dasyurus maculatus*, which has a similar body mass to that estimated for *Anatoliadelphys*, and which is an agile predator that is predominantly terrestrial, but which is nevertheless capable of climbing well. However, the larger medial epicondyle and supinator crest of the humerus and the much larger peroneal process of the calcaneus of *Anatoliadelphys* suggest that it was capable of more powerful grasping than in *D*. *maculatus* in which (as in all dasyuromorphians) the hallux is reduced; nevertheless, *D*. *maculatus* and other dasyurids (including *Sarcophilus*) are capable of grasping [52, 154, 155, 165].

**Fig 37:**
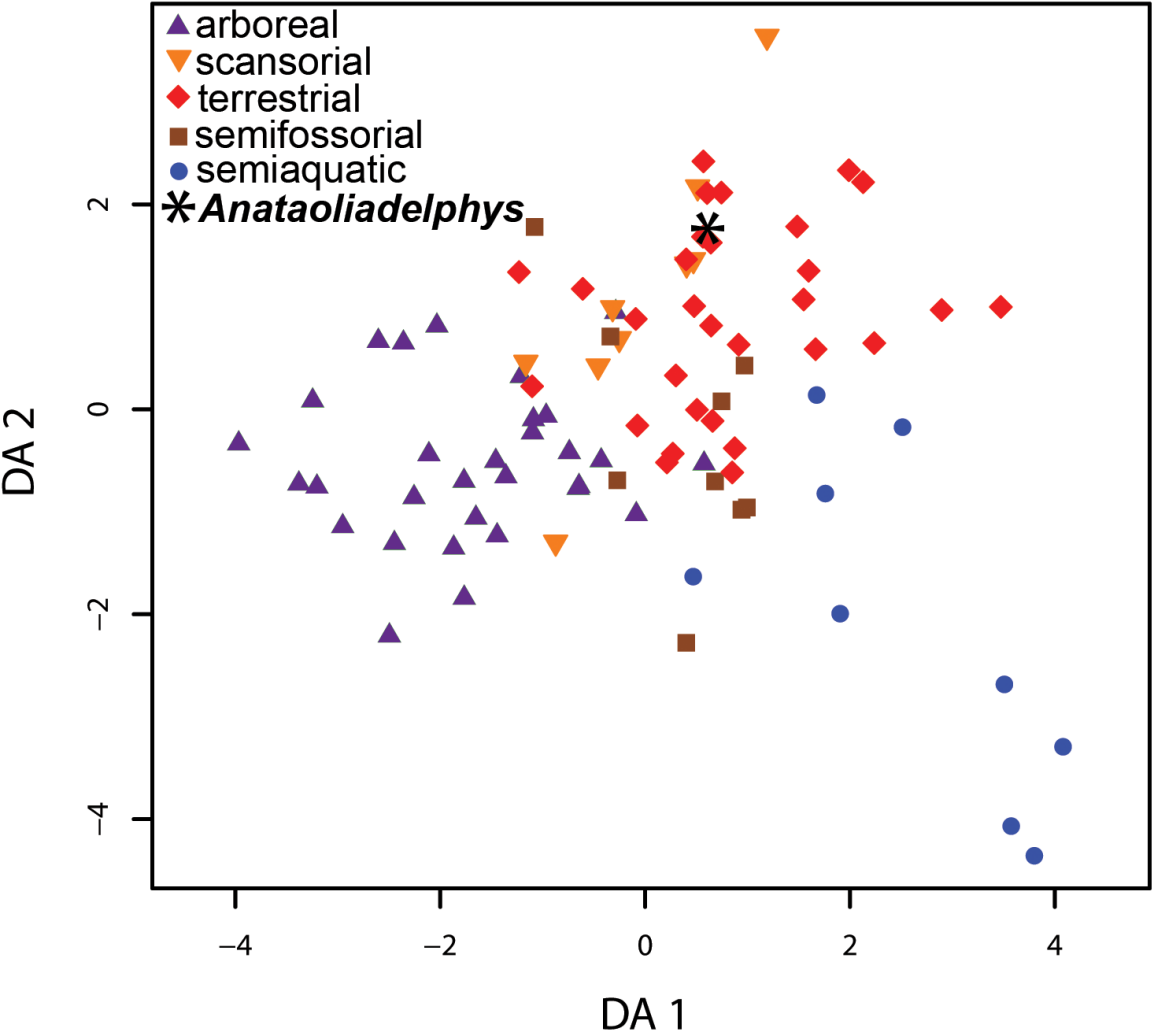
Phylogenetically flexible discriminant analysis of postcranial indices for *Anatoliadelphys maasae* and modern mammals. The modern mammals were classified as representing either arboreal, scansorial, semiaquatic, semifossorial or terrestrial locomotor modes. This analysis classified *Anatoliadelphys* as having a probability of 77% of being scansorial, 17% of being semifossorial and 6% of being terrestrial. The plot shows the first two discriminant axes (DAs) only.

Our phylogenetically-flexible discriminant function analysis of locomotor mode based on a dataset of 24 postcranial indices (Table 5 and Fig 37) performed well at correctly classifying extant taxa, with only 16% misclassified. Based on the same dataset, this analysis classified *Anatoliadelphys* as having a probability of 77% of being scansorial, 17% of being semifossorial and 6% of being terrestrial. These results are therefore highly congruent with our qualitative analysis of its postcranial skeleton, which also supports scansorial locomotion.

**Table 5:** 24 postcranial indices from Chen and Wilson [76] calculated for *Anatoliaelphys maasae*, based on measurements from AÜJM 2002–25.

Index	Value
Humeral mid-shaft transverse diameter divided by humeral length (= “Humeral Robustness Index”)	0.10
Humeral proximal end width divided by humeral length (= “Humeral Proximal End Index”)	0.25
Humeral epicondylar width divided by humeral length (= “Humeral Epicondylar Index”)	0.36
Transverse diameter of humerus divided by humeral proximal end width	0.38
Humeral head length divided by humeral length (= “Humeral Head Robustness Index”)	0.16
Humeral head width divided by humeral proximal end width	0.69
Deltopectoral crest width divided by humeral proximal end	0.22
Deltopectoral crest width divided by the mid-shaft width of humerus (= “Deltopectoral Crest Index”)	0.59
Deltopectoral crest width divided by humeral distal end	0.15
Ulnar length divided by humeral length	1.18
Olecranon process length divided by ulnar length (= “Olecranon Process Length Index”)	0.18
Olecranon process length divided by humeral length	0.22
Radial length divided by humeral length (= “Brachial Index”)	0.89
Radial length divided by ulnar length	0.75
Olecranon process length divided by radial length	0.24
Ilium length divided by entire pelvic length (= “Ilium Robustness Index”)	0.60
Proximal extension of greater trochanter divided by femoral length (= “Gluteal Index”)	0.03
Transverse diameter divided by femur length (= “Femoral Robustness Index”)	0.09
Tibial length divided by fibular length (= “Crural Index”)	1.03
Lengths of the humerus and radius divided by lengths of the femur and tibia (= “Intermembral Index”)	0.79
Transverse diameter of tibia divided by tibial length (= “Tibial Robustness Index”)	0.07
Calcaneal body length divided by calcaneal length (= “Calcaneal Body Robustness Index”)	0.25
Calcaneal tuber length divided by calcaneal length (= “Calcaneal Tuber Robustness Index”)	0.43
Calcaneal body length divided by calcaneal tuber length	0.58

### Phylogenetic relationships of *Anatoliadelphys*

Several metatherians show broadly similar craniodental adaptations to *Anatoliadelphys*, namely enlarged upper and lower third premolars and features associated with the generation of high bite forces; these include the Late Cretaceous North American stagodontids [29, 75, 101, 142, 145–147], *Eobrasilia coutoi*, *Didelphopsis cabrerai* and *Gaylordia macrocynodonta* from the early Eocene Itaboraí fauna of Brazil [166–172], various South American Cenozoic sparassodonts [148], the probable dasyuromorphian *Malleodectes* from the Miocene of Australia [150, 151], and the extant dasyurid *Sarcophilus harrisii*. Qualitative analysis of available data suggests that these similarities are most likely homoplastic, as discussed here.

*Anatoliadelphys* lacks the tall paraconid and high, bladelike paracristid with a “keyhole”-like carnassial notch characteristic of stagodontid lower molars, and there are major differences in the structure of the upper molars, with stagodontids having a straight centrocrista on M3 (strongly v-shaped in *Anatoliadelphys*, with the premetacrista contacting stylar cusp D) and well-developed conules (absent or indistinct in *Anatoliadelphys*) [66, 101, 147]. Isolated tarsals tentatively referred to the stagodontids *Eodelphis* and *Didelphodon* by Szalay [56] are highly distinctive, and exhibit a number of probable apomorphies absent in *Anatoliadelphys*, including a circular calcaneocuboid facet, and a distally-placed and obliquely-oriented sustentacular facet [56].

*Eobrasilia* is too poorly known to be meaningfully compared with *Anatoliadelphys*. An isolated right upper molar (DGM 896-M(a), an M2 or M3) was tentatively referred to *E*. *coutoi* by Marshall [167], but was subsequently referred by him to a separate taxon, *Zeusdelphys complicatus* [168, 169]. In any case, DGM 896-M(a) differs markedly from the upper molars of *Anatoliadelphys*: in DGM 896-M(a), StB and StD are both very large and subequal in height (StD is much larger than StB in *Anatoliadelphys*), and the enamel is heavily wrinkled, unlike in *Anatoliadelphys*, and it further differs from *Anatoliadelphys* in lacking distinct cingula anterior and posterior to the protocone. *Didelphopsis cabrerai* and *Gaylordia macrocynodonta*, meanwhile, lacks the strong posterior increase in molar size, enormous protoconid and very reduced paraconid and metaconid of *Anatoliadelphys*. *Didelphopsis cabrerai* and a second, less specialised *Gaylordia* species from Itaboraí, *G*. *mater* are characterised by a weakly v-shaped centrocrista on M3 (strongly v-shaped in *Anatoliadelphys*, with the premetacrista contacting stylar cusp D), and a prominent stylar cusp C (absent in *Anatoliadelphys*), and they lack prominent pre- and post-cingula [168–172].

In the upper molars of sparassodonts, the centrocrista (where not lost through fusion of the paracone and metacone) is straight on all upper molars, unlike the strongly v-shaped centrocrista of the M3 of *Anatoliadelphys*, pre- and post-cingula are absent, and the protocone is often reduced, whilst in the lower molars, the paraconid is consistently well-developed in sparassodonts [32, 66]; there are also major differences in tarsal structure between sparassodonts and *Anatoliadelphys* [56].

Finally, *Anatoliadelphys* shares with most dasyuromorphians (including the durophagously-adapted *Malleodectes* [151]) the presence of a StD that is distinctly larger than StB on M3, but it lacks the characteristic australidelphian apomorphies seen in the tarsus of dasyuromorphians, most obviously the fusion of the ectal and sustentacular facets (the “continuous lower ankle joint pattern”) [56, 125, 164]. In summary, although *Anatoliadelphys* shares some derived craniodental features with a wide range of metatherians, it is likely that most (if not all) are convergent, reflecting the functional demands of a durophagous diet. Broadly similar adaptations have also evolved multiple times in eutherians, for example in the “condylarth” *Periptychus* [10] and the apternodontid “insectivore” *Apternodus* [173].

This qualitative interpretation is supported by the results of our Bayesian undated and “tip-and-node dating” analyses. In the both analyses, *Anatoliadelphys* falls within Marsupialiformes but outside Marsupialia, and it does not form a clade with *Didelphodon* or with the possible sparassodont *Mayulestes*. In the undated analysis, *Anatoliadelphys* is part of a trichotomy that also includes Peradectidae and a clade that includes Marsupialia, *Didelphodon*, *Herpetotherium* and *Asiatherium*. In the dated analysis, *Anatoliadelphys* forms a clade with Peradectidae, which receives moderate support (Bayesian posterior probability = 0.77), whilst *Didelphodon* is again closer to Marsupialia.

Overall, our phylogenetic results are broadly similar to other recent analyses [37, 75, 80, 103, 148, 174, 175], although some relationships within Marsupialiformes remain to be confidently resolved. Notably, our analyses recovered relationships within Marsupialia that are congruent with recent molecular studies [5, 176–178], in contrast to the phylogenetic analysis of Wilson et al. [75], which failed to recover monophyly of Australidelphia.

Unambigous synapomorphies for Metatheria, Marsupialiformes, Marsupialia and (for the dated analysis) *Anatoliadelphys*+Peradectidae are given in Table 6. In the undated analysis, loss of the posterior cingulid optimises as an unambiguous synapomorphy of Marsupialia (secondarily reversed in dasyuromorphians), as previously proposed by Voss and Jansa [27]; *Anatoliadelphys* retains a very faint posterior cingulid on m3 and m4, supporting a position outside Marsupialia. In the dated analysis, meanwhile, the sole unambiguous synapomorphy of Marsupialia is subdivision of the calcaneocuboid facet of the calcaneus into three distinct facets; this morphology is present in australidelphians and paucituberculatans, whilst didelphids have distinct distal and proximal calcaneocuboid facets, and that of *Herpetotherium* also appears to be subdivided into two distinct facets (one laterodorsal and one medioventral [49, 56, 119, 125, 164, 179]). The simple, concave calcaneocuboid facet of *Anatoliadelphys* is congruent with a position outside Marsupialia. A full list of morphological synapomorphies for all clades present in our undated and dated analyses, under both Accelerated Transformation (ACCTRAN) and Delayed Transformation (DELTRAN), is given in S4 Text.

**Table 6:** Unambiguous morphological synapomorphies (identified using the maximum parsimony criterion in PAUP* 4.0a152) for selected clades recovered in our dated and undated phylogenetic analyses (see Figs 38 and 39). A full list of morphological synapomorphies for all clades present in our undated and dated analyses, under both Accelerated Transformation (ACCTRAN) and Delayed Transformation (DELTRAN), is given in S4 Text.

Analysis	Clade	Unambiguous synapmorphies	Consistency Index
Undated	Metatheria	four upper molars (148: 0)	1
		C1 single-rooted (167: 1)	1
		staggering of i2 (168: 1)	1
		marsupial pattern of dental replacement (171: 1)	1
		angular process of mandible medially inflected (174: 1)	0.333
		palatal process of premaxilla reaches C1 alveolus (199: 1)	0.167
		sulcus for stapedial artery on petrosal promontorium absent (252: 1)	0.5
	Marsupialiformes	paracone smaller than metacone (151: 2)	0.3
		Intersection of cristid obliqua with m2 trigonid labial to protocristid/metacristid notch (156: 1)	0.333
		petrosal contributes to lateral wall of epitympanic recess (244: 1)	1
		posterior cingulid present on lower molars (257: 1)	0.333
	Marsupialia	no raised tuberosity for rectus femoris on ilium (70: 0)	0.222
		longest dimension of ectal facet is anteromedial to posterolateral (109: 1)	0.2
		posterior cingulid absent on lower molars (257: 0)	0.333
Dated	Metatheria	none	N/A
	Marsupialiformes	paracone smaller than metacone (151: 2)	0.375
		petrosal contributes to lateral wall of epitympanic recess (244: 1)	1
		posterior cingulid present on lower molars (257: 1)	0.2
	Marsupialia	calcaneocuboid facet of the calcaneus subdivided into three facets (123: 2)	1
	*Anatoliadelphys*+ Peradectidae	none	N/A

**Fig 38:**
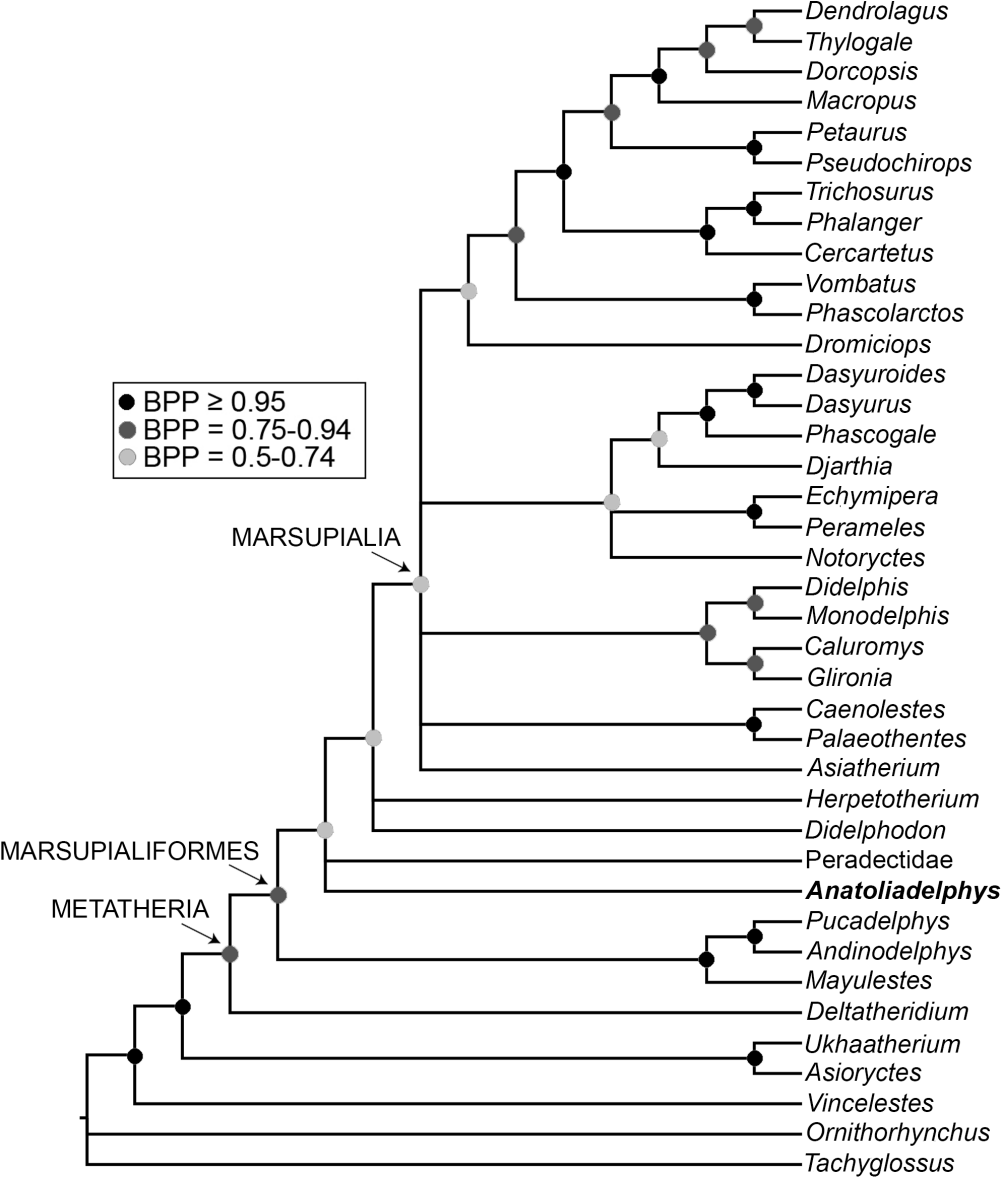
Phylogeny of Metatheria based on Bayesian undated analysis of total evidence dataset. The dataset comprises 259 morphological characters and 9012 bp of molecular sequence data from 5 nuclear genes (*APOB*, *BRCA1*, *IRBP*, *RAG1* and *VWF*). The topology represents a 50% majority rule consensus tree of post-burn-in trees. Abbreviations: BPP, Bayesian posterior probability.

**Fig 39:**
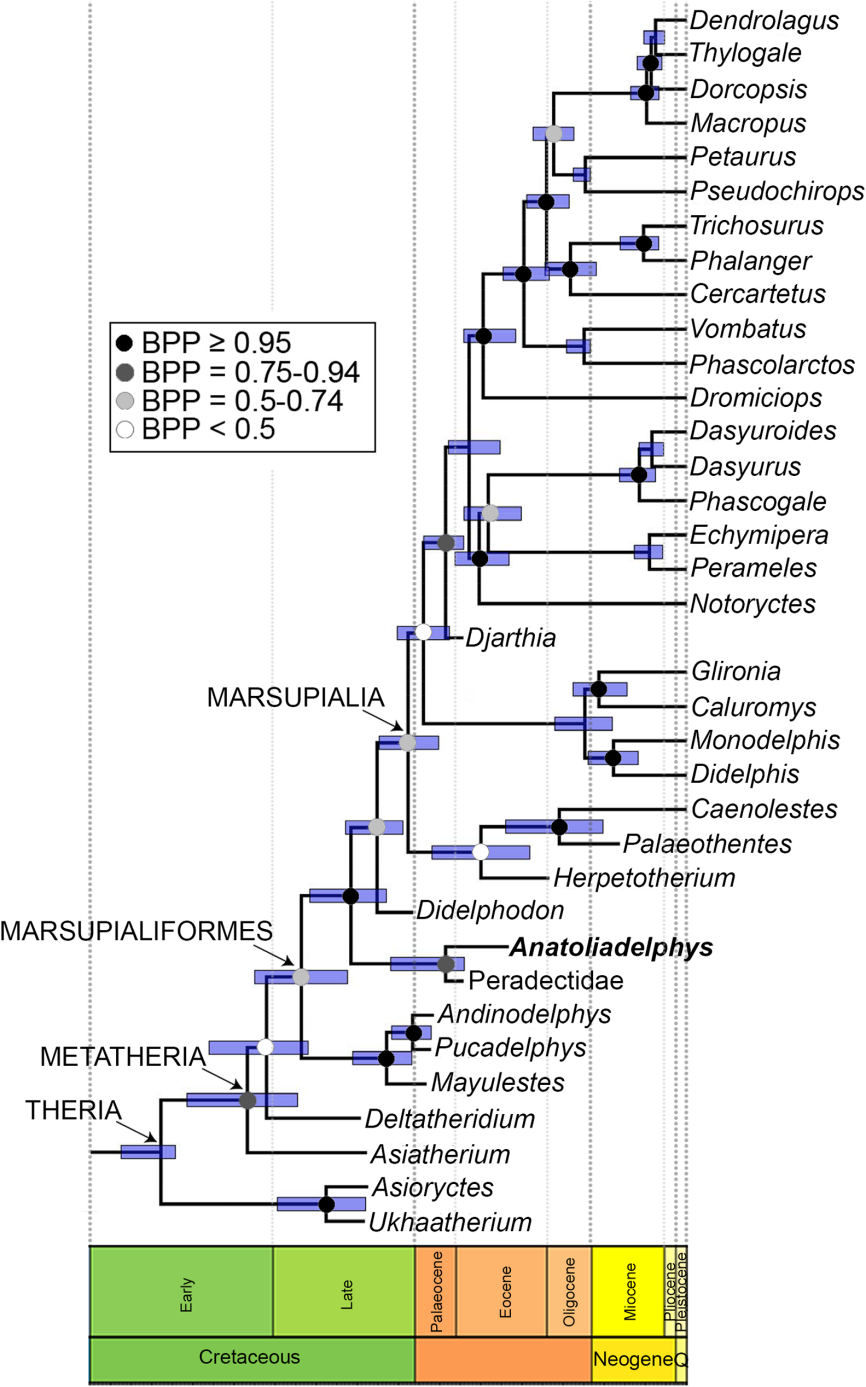
Phylogeny of Metatheria based on Bayesian “tip-and-node dating” analysis of total evidence dataset. The dataset comprises 259 morphological characters and 9012 bp of molecular sequence data from 5 nuclear genes (*APOB*, *BRCA1*, *IRBP*, *RAG1* and *VWF*), assuming a single Independent Gamma Rates (IGR) clock model, “diversity” sampling, and with temporal information provided in the form of tip ages and also constraints (specified as offset exponential distributions) on the ages of selected nodes. The topology represents a maximum clade credibility (MCC) tree. Nodes without Bayesian posterior probabilities were used as age constraints, which required them to be constrained as monophyletic a priori. Blue bars at nodes represent 95% Highest Posterior Densities (HPDs) on node ages. Abbreviations: BPP, Bayesian posterior probability.

The dated analysis resulted in divergence dates that are broadly congruent with recent molecular studies [5], with the first divergence within Marsupialia estimated at 68.2 MYA (95% HPD: 60.7–75.2 MYA). *Anatoliadelphys* was estimated as having diverged from Peradectidae 59.0 MYA (95% HPD 54.4–72.4 MYA).

## Discussion

Perhaps the most remarkable aspect of *Anatoliadelphys maasae* is its large size. With an estimated body mass of 3–4 kg, it is one of the largest metatherians known from the northern hemisphere, together with two North American species, namely the extant Virginia opossum (*Didelphis virginiana*; mean body mass 2.4 kg [70]), and the Late Cretaceous stagodontid *Didelphodon vorax* (estimated body mass 2.1–6.2 kg [75]). With the exception of *D*. *virginiana* (which dispersed to North America from South America <1 million years ago [22]), the largest Cenozoic metatherians previously known from the northern hemisphere were the early Eocene North American peradectids *Mimoperadectes labrus* and *M*. *houdei* [20, 180]), which probably weighed ~250g, i.e. an order of magnitude smaller than *Anatoliadelphys*.

*Anatoliadelphys* is also unusual in exhibiting obvious craniodental adaptations for carnivory (probably meso- or hypocarnivory, rather than hypercarnivory) and durophagy (perhaps for crushing bone, hard shelled invertebrates, or both). The Cretaceous northern hemisphere deltatheroidans and stagodontids were carnivorously adapted [6, 7, 9, 66], and the large, broad premolars of stagodontids also suggest durophagy [6, 29, 101, 145, 181]. Numerous carnivorous and durophagous metatherians are also known from the Cenozoic of South America and Australia [12, 15, 17, 32, 104, 148, 151, 182, 183]. However, prior to the discovery of *Anatoliadelphys*, all known Cenozoic fossil metatherians from the northern hemisphere were characterised by relatively generalised tribosphenic molars and unspecialised premolars [20, 21, 28, 184], suggesting that they were primarily insectivorous (although some peradectids may have been at least partially frugivorous [95]). *Anatoliadelphys* therefore reveals previously unsuspected ecomorphological diversity among northern hemisphere metatherians during the Cenozoic.

*Anatoliadelphys* demonstrates that at least one metatherian lineage evolved to successfully occupy a small-medium meso- or hypocarnivore niche in the northern hemisphere during the early Palaeogene. Numerous carnivorous fossil eutherians are known from the northern hemisphere during this time period, including carnivoromorphians, mesonychians, pantolestids, didymoconids, and oxyaenid and hyaenodontid creodonts [10, 185]. Hyaenodontids are also known from the early Palaeogene of Africa, where they likely originated [186]. Many of these carnivorous eutherians were similar in size (1–10 kg) to *Anatoliadelphys*, and so represent potential ecological competitors [185].

However, although still poorly known, the UCF mammal fauna appears to be highly endemic (Table 7), and carnivorous eutherians have yet to be found there. As noted by Metais et al. [94], the presence of multiple species of the pleuraspidotheriid *Hilalia* in the Lutetian (44–43 MYA) UCF mammal fauna is particularly striking: the youngest record of Pleuraspidotheriidae from Europe is latest Palaeocene, i.e. 12–14 million years older [189]. This “anachronistic” presence of pleuraspidotheriids suggests that the UCF may preserve a mammal fauna that evolved largely in isolation during the early Cenozoic, with only limited biogeographical connectivity to Laurasia and Africa [89, 94]. This is congruent with geological evidence that this region of Turkey was an island for at least part of the early Cenozoic [188, 190]. If *Anatoliadelphys* evolved as part of an isolated mammalian fauna in which carnivorous eutherians were absent, then it may in fact provide further support for the hypothesis that metatherians are competitively inferior to eutherians, due to differences in traits relating to reproductive mode and associated developmental constraints, and/or differences in metabolic rate [23, 191–195]. This interpretation is reinforced by the apparent absence of mainland Laurasian metatherians that show similar specialisations to *Anatoliadelphys*.

**Table 7:** The mammal fauna described to date from the Uzunçarşidere Formation.

Taxon	Specimen(s)	Higher level relationships	Reference
*Anatoliadelphys maasae*	AÜJM 2002–25	Marsupialiformes	this study
Unnamed bunodont mammal	AK95-19	?Marsupialiformes	[92]
Unnamed dilambdodont mammal	AK94-8, AK95-34, AK95-35, AK95-37	?Marsupialiformes or? Afrosoricida	[91]
Unnamed embrithopod	AK94-2, AK94-5	Embrithopoda; Paenungulata; Afrotheria	[91, 92]
Unnamed tribosphenic mammal	AK95-36	Theria	[91]
*Hilalia saribeya*	AK95-50R, AK95-50L, AK94-1, AK95-4, AK95-5, AK95-45, AK95-89, AÜJM99-3, AÜJM99-5, EOU-UCF-1	Pleuraspidotheriidae; “Condylarthra”	[93]
*Hilalia selanneae*	AK95-28R, AK95-28L, AK95-29, AÜJM99-20	Pleuraspidotheriidae; “Condylarthra”	[93]
*Hilalia sezerorum*	AK95-26, AÜJM2000-6	Pleuraspidotheriidae; “Condylarthra”	[93]
*Hilalia robusta*	AÜJM99-17R, AÜJM99-17L, AÜJM99-30	Pleuraspidotheriidae; “Condylarthra”	[93]
Unnamed mammal (?*Hilalia* sp.)	AK95-20	?Pleuraspidotheriidae	[92, 93]
*Hypsamasia seni*	AK95-52	Embrithopoda; Paenungulata; Afrotheria	[92]
*Palaeoamasia* sp.		Embrithopoda; Paenungulata; Afrotheria	[187, 188]
?Proboscidean	AK95-1, AUGD 2000–062, AUGD 2000–045, AUGD 99-12a, AUGD 99-12b, AUGD 99–14, AUGD 2000–048	?Proboscidea; Paenungulata; Afrotheria	[92, 188]

## Supporting information

S1 File Total evidence (morphological and molecular) character matrix for analysing phylogenetic relationships of metatherians using Bayesian undated and “tip-and-node dating” approaches. (NEX)

S1 Text Comparative material of modern and fossil metatherians examined in this study. (DOCX)

S2 Text Morphological character scores of *Anatoliadelphys* and *Didelphodon* for phylogenetic analysis. (DOCX)

S3 Text Assumed age ranges (in MYA) for fossil taxa included in phylogenetic analysis. (DOCX)

S4 Text Morphological synapomorphies for all clades present in our undated and dated analyses, under both Accelerated Transformation (ACCTRAN) and Delayed Transformation (DELTRAN). (TXT)
