# Correction: Skeleton of an unusual, cat-sized marsupial relative (Metatheria: Marsupialiformes) from the middle Eocene (Lutetian: 44-43 million years ago) of Turkey

**DOI:** 10.1371/journal.pone.0188132

**Published:** 2017-11-09

**Authors:** A. Murat Maga, Robin M. D. Beck

The following information is missing from the Funding section: We acknowledge funding from National Geographic Society for the fieldwork in Uzuncarsidere that resulted in the discovery of the specimen (grant no 7086–01).
