# Supplementary material for: Skeleton of an unusual, cat-sized marsupial relative (Metatheria: Marsupialiformes) from the middle Eocene (Lutetian: 44-43 million years ago) of Turkey
Source: PLoS One. 2017 Aug 16;12(8):e0181712. doi: 10.1371/journal.pone.0181712 (PMC5559079; doi:10.1371/journal.pone.0181712)
Supplement: S1 Text — (DOCX) [file pone.0181712.s002.docx]

**S1 Text. Comparative material of modern and fossil metatherians examined in this study.**

As part of this study, comparative material of modern and fossil metatherians were examined in the following collections: Australian Museum, Sydney, Australia (prefix AM); American Museum of Natural History, New York, USA (prefix AMNH); Vertebrate Paleontology Laboratory, Jackson School of Geosciences, The University of Texas at Austin, Austin, Texas (prefix TMM)**,** Smithsonian National Museum of Natural History, Washington DC, USA (prefix USNM).

MODERN

**Dasyuridae**

*Dasyurus geoffroii*

AM: M1427, M1541, M10370, P756, P757

*Dasyurus hallucatus*

AM: M5044, M8673, M9081, M21230, M22902, M26350

TMM: M6921

*Dasyurus maculatus*

AM: M1666, M4330, M4720, M6748, M7388, M7399, S2124

USNM: 238352, 579573

*Dasyurus spartacus*

AM: M37432

*Dasyurus viverrinus*

AM: M3776, M5269, M6525, M6604, M7389

*Phascogale tapoatafa*

AM: M35626, M35919, M37467, M37468, M37469

*Sarcophilus harrisii*

AM: M23599, M44955

USNM: 238340, A22816

**Didelphidae**

*Didelphis marsupialis*

USNM: A14213

*Didelphis virginiana*

TMM: M1895, M2487, M2517

USNM: A23330

**Thylacinidae**

*Thylacinus cynocephalus*

AM: M822, M1821,

FOSSIL

**incertae sedis**

“Bridger Metatherian Group I” (BMG I)

AMNH: FM29110

**Herptotheriidae**

*Herptotherium* sp.

AMNH: FM22304
