# Supplementary material for: Skeleton of an unusual, cat-sized marsupial relative (Metatheria: Marsupialiformes) from the middle Eocene (Lutetian: 44-43 million years ago) of Turkey
Source: PLoS One. 2017 Aug 16;12(8):e0181712. doi: 10.1371/journal.pone.0181712 (PMC5559079; doi:10.1371/journal.pone.0181712)
Supplement: S2 Text — (DOCX) [file pone.0181712.s003.docx]

**S2 Text. Morphological character scores of *Anatoliadelphys* and *Didelphodon* for phylogenetic analysis.**

The morphological character matrix used comprises 259 characters are from Beck et al. ([1]; see also [2-8]), who included 33 fossil and modern metatherians, to which we have added *Anatoliadelphys* and *Didelphodon*. Characters marked by * (49 in total) were treated as ordered/additive in the phylogenetic analysis. The total evidence matrix that includes these morphological character scores, together with 9012 base pairs of sequence data from five nuclear protein-coding genes (*APOB*, *BRCA1*, *IRBP*, *RAG1* and *VWF*), is available as S2 File.

1. Atlantal foramen:

0 absent

1 present

***Anatoliadelphys*: 0**

***Didelphodon*: ?**

2. Atlas transverse foramen:

0 absent

1 present

***Anatoliadelphys*: 0**

***Didelphodon*: ?**

3. Atlas, posterior extent of transverse process:

0 absent

1 present

***Anatoliadelphys*: 0**

***Didelphodon*: ?**

*4. Atlas, cranial facets shape:

0 only concave

1 dorsal edge curved

2 dorsal edge envelops the occipital condyle medially

***Anatoliadelphys*: 0**

***Didelphodon*: ?**

5. Atlas, transverse process ventral to atlantal foramen groove:

0 absent

1 present

***Anatoliadelphys*: 0**

***Didelphodon*: ?**

6. Atlas ventral arches:

0 open

1 complete

***Anatoliadelphys*: ?**

***Didelphodon*: ?**

7. Atlas intercentrum:

0 absent

1 present with no fusion

***Anatoliadelphys*: ?**

***Didelphodon*: ?**

8. Axis transverse foramen:

0 open

1 enclosed

***Anatoliadelphys*: 0**

***Didelphodon*: ?**

9. Axis posterior spinous process extension:

0 absent

1 present

***Anatoliadelphys*: 1**

***Didelphodon*: ?**

10. Axis extra pair of transverse processes on the ventral surface of the body:

0 absent

1 present

***Anatoliadelphys*: 0**

***Didelphodon*: ?**

*11. Axis anterior facets (prezygapophyses) and dens connection:

0 not linked

1 linked

2 facets extend ventral to the dens

***Anatoliadelphys*: 0**

***Didelphodon*: ?**

12. Suture between rib and axis is visible:

0 no

1 suture visible

***Anatoliadelphys*: ?**

***Didelphodon*: ?**

*13. Axis-C3-C4 fusion:

0 no fusion

1 axis and C3 fused

2 axis-C3-C4 fused

***Anatoliadelphys*: 0**

***Didelphodon*: ?**

14. C3-C4 ventral sagittal process:

0 absent

1 present

***Anatoliadelphys*: ?**

***Didelphodon*: ?**

15. C5 transverse process displays two heads:

0 absent

1 present

***Anatoliadelphys*: ?**

***Didelphodon*: ?**

16. C5 transverse process heads overlap transversally:

0 absent

1 present

***Anatoliadelphys*: ?**

***Didelphodon*: ?**

17 C5 and T1 body length:

0 subequal or C5 longer than T1

1 C5 shorter than T1

***Anatoliadelphys*: ?**

***Didelphodon*: ?**

18. C6 transverse process posterior extension:

0 absent

1 present

***Anatoliadelphys*: ?**

***Didelphodon*: ?**

*19. C6 spinous process:

0 absent

1 protuberance

2 lamina

***Anatoliadelphys*: ?**

***Didelphodon*: ?**

*20. C7 transverse foramen:

0 absent

1 incipient

2 present

***Anatoliadelphys*: 2**

***Didelphodon*: ?**

21. Articulation among cervical vertebrae:

0 only bodies articulate

1 prezygapophyses and postzygapophyses in addition to bodies articulate

***Anatoliadelphys*: 1**

***Didelphodon*: ?**

22. Articulation between C4-C5 bodies type:

0 flat

1 saddle

***Anatoliadelphys*: 0**

***Didelphodon*: ?**

23. T1 transverse process:

0 absent

1 present

***Anatoliadelphys*: ?**

***Didelphodon*: ?**

24. T1 transverse process level:

0 level with prezygapophysis

1 lower than prezygapophysis

***Anatoliadelphys*: ?**

***Didelphodon*: ?**

25. First thoracic vertebra with a tall spinous process (relative to other vertebrae):

0 T1

1 T2

***Anatoliadelphys*: ?**

***Didelphodon*: ?**

*26. First thoracic vertebrae with prezygapophysis facing laterally:

0 T1

1 T2

2 T3

***Anatoliadelphys*: ?**

***Didelphodon*: ?**

27. Thoracic vertebrae, intervertebral foramen enclosed:

0 absent

1 present

***Anatoliadelphys*: ?**

***Didelphodon*: ?**

28. Last lumbar vertebra, foramen on dorsal arch:

0 absent

1 present

***Anatoliadelphys*: ?**

***Didelphodon*: ?**

29. Metapophyses in third lumbar vertebra anterior to last:

0 absent

1 anterior

2 dorsal

***Anatoliadelphys*: ?**

***Didelphodon*: ?**

30. Caudal vertebrae, count:

0 9 or fewer

1 11 or more

***Anatoliadelphys*: 1**

***Didelphodon*: ?**

31. Caudal vertebrae, hyperexpanded chevrons:

0 absent

1 present

***Anatoliadelphys*: 0**

***Didelphodon*: ?**

32. Prehensile tail:

0 absent

1 present

***Anatoliadelphys*: ?**

***Didelphodon*: ?**

33. Sternal cartilages, ossification (= sternal ribs):

0 absent

1 present

***Anatoliadelphys*: ?**

***Didelphodon*: ?**

34. Ribs, articulation with sternum:

0 with single sternebra

1 with two sternebrae

***Anatoliadelphys*: ?**

***Didelphodon*: ?**

*35. Coracoid process:

0 separate and large

1 hook-like process

2 small process

3 small protuberance or absent

***Anatoliadelphys*: 2**

***Didelphodon*: ?**

36. Ventral extension of acromion:

0 ventral (beyond) glenoid

1 does not extend beyond level of glenoid

***Anatoliadelphys*: ?**

***Didelphodon*: ?**

37. Crest on medial aspect of scapula, near the caudal border:

0 absent

1 present

***Anatoliadelphys*: ?**

***Didelphodon*: ?**

38. Separate large interclavicle:

0 absent

1 present

***Anatoliadelphys*: ?**

***Didelphodon*: ?**

*39. Infraspinous fossa width:

0 less than 1/4 its length

1 between 1/2 and 1/4 its length

2 more than 1/2 its length

***Anatoliadelphys*: ?**

***Didelphodon*: ?**

*40. Scapular spine width at neck level:

0 narrower than infraspinous fossa

1 subequal

2 wider than infraspinous fossa

***Anatoliadelphys*: ?**

***Didelphodon*: ?**

*41. Infraspinous/supraspinous fossa width at neck level:

0 infraspinous fossa narrower

1 subequal

2 supraspinous fossa narrower

***Anatoliadelphys*: 1**

***Didelphodon*: ?**

42. Humerus, medial relief for teres major m:

0 absent

1 present

***Anatoliadelphys*: 1**

***Didelphodon*: ?**

*43. Humerus, lateral ridge:

0 absent

1 ridge or crest

2 massive crest continuous with deltopectoral crest

***Anatoliadelphys*: 2**

***Didelphodon*: ?**

44. Humerus, capitulum for radius:

0 spherical

1 cylindrical

***Anatoliadelphys*: 0**

***Didelphodon*: ?**

45. Humerus, entepicondylar foramen:

0 absent

1 present

***Anatoliadelphys*: 1**

***Didelphodon*: ?**

*46. Humerus, olecranon fossa or foramen:

0 absent

1 small fossa

2 large fossa

3 foramen

***Anatoliadelphys*: 1**

***Didelphodon*: ?**

47. Humerus, laminar supinator crest:

0 absent

1 present

***Anatoliadelphys*: 1**

***Didelphodon*: ?**

48. Humerus, greater tuberosity height relative to humeral head height:

0 equal or greater tuberosity is lower

1 greater tuberosity is higher

***Anatoliadelphys*: 0**

***Didelphodon*: ?**

49. Humerus, extension of the deltoid crest:

0 restricted to proximal half of humerus

1 reaches distal half

***Anatoliadelphys*: 0**

***Didelphodon*: ?**

50. Humerus trochlea:

0 absent

1 present

***Anatoliadelphys*: 1**

***Didelphodon*: ?**

51. Humerus, proximal extension of capitulum and trochlea:

0 longer proximal extension of trochlea

1 equal

***Anatoliadelphys*: 1**

***Didelphodon*: ?**

52. Humerus, medial epicondyle size:

0 small

1 large

***Anatoliadelphys*: ?**

***Didelphodon*: ?**

53. Humerus distal end size:

0 small

1 large

***Anatoliadelphys*: ?**

***Didelphodon*: ?**

54. Humerus, head shape:

0 symmetrical/ovoid

1 mediolaterally compressed

***Anatoliadelphys*: 0**

***Didelphodon*: ?**

55. Humerus, capitulum lateral extension:

0 absent

1 present

***Anatoliadelphys*: 1**

***Didelphodon*: ?**

56. Humerus, shaft curvature:

0 straight

1 curved

***Anatoliadelphys*: 1**

***Didelphodon*: ?**

57. Ulna, proximal epiphysis, distal most portion of articular surface for humerus:

0 ulnar area is less than 1/2 of radial one

1 more

***Anatoliadelphys*: 1**

***Didelphodon*: ?**

58. Ulna, coronoid process:

0 absent

1 present

***Anatoliadelphys*: 1**

***Didelphodon*: ?**

59. Distal process of ulna:

0 spherical, contributing to a ball and socket articulation

1 nonspherical

***Anatoliadelphys*: ?**

***Didelphodon*: ?**

60. Radius, articular facet for humerus:

0 circular

1 antero-posteriorly compressed

***Anatoliadelphys*: 0**

***Didelphodon*: ?**

*61. Lunate:

0 absent or fused to other elements

1 very small

2 relatively large, with contacts with other carpals

***Anatoliadelphys*: ?**

***Didelphodon*: ?**

62. Prepollex:

0 absent

1 present

***Anatoliadelphys*: ?**

***Didelphodon*: ?**

*63. Distolateral process of scaphoid separates dorsally lunate from magnum:

0 absent

1 present

2 present, separates dorsally lunate from magnum

***Anatoliadelphys*: ?**

***Didelphodon*: ?**

64. Proximal aspect of magnum:

0 single head

1 saddle-shape articulation with lunate

***Anatoliadelphys*: ?**

***Didelphodon*: ?**

65. Magnum, facet with MC III:

0 flat, convex or keeled

1 fully concave

***Anatoliadelphys*: ?**

***Didelphodon*: ?**

66. Trapezium, number of distal heads:

0 one head

1 two heads

***Anatoliadelphys*: ?**

***Didelphodon*: ?**

67. Plane of the articular surface trapezium-Mc I in relation to other carpal-metacarpal articulations:

0 not parallel

1 somewhat parallel

***Anatoliadelphys*: ?**

***Didelphodon*: ?**

68. Both MC I and MC V are hyper-reduced in relation to other metacarpals:

0 hyperreduction

1 no hyperreduction

***Anatoliadelphys*: ?**

***Didelphodon*: ?**

69. Magnum and unciform proximal extension:

0 similarly proximal

1 unciform more proximal

***Anatoliadelphys*: ?**

***Didelphodon*: ?**

70. Tuberosity for rectus femoris m:

0 no relief

1 protuberance

2 depression

***Anatoliadelphys*: 1**

***Didelphodon*: ?**

71. Relative size of obturator foramen:

0 smaller or equal to acetabulum

1 larger than acetabulum

***Anatoliadelphys*: 1**

***Didelphodon*: ?**

72. Hyperdevelopment of iliopubic process:

0 absent

1 present

***Anatoliadelphys*: 0**

***Didelphodon*: ?**

73. Pelvis, acetabular notch:

0 absent

1 present

***Anatoliadelphys*: 1**

***Didelphodon*: ?**

74. Epipubic bones:

0 absent

1 present

***Anatoliadelphys*: 1**

***Didelphodon*: ?**

75. Epipubic bones proximal size:

0 short

1 long

***Anatoliadelphys*: ?**

***Didelphodon*: ?**

76. Mediolateral constriction in greater trochanter of femur:

0 absent

1 present

***Anatoliadelphys*: 0**

***Didelphodon*: ?**

77. Relative height of greater trochanter / femoral head:

0 greater trochanter is lower or equal

1 higher

***Anatoliadelphys*: 0**

***Didelphodon*: ?**

78. Femur, lesser trochanter:

0 absent

1 present

***Anatoliadelphys*: 1**

***Didelphodon*: ?**

79. Fibula proximal medial accessory shelf:

0 absent

1 present

***Anatoliadelphys*: 0**

***Didelphodon*: ?**

80. Fibula proximal posterior extension beyond the area of contact with the tibia:

0 does not extend posteriorly

1 extends

***Anatoliadelphys*: 1**

***Didelphodon*: ?**

81. Fibula lateral distal process:

0 absent

1 present

***Anatoliadelphys*: 1**

***Didelphodon*: ?**

82. Articulation between femur and fibula:

0 absent

1 present

***Anatoliadelphys*: ?**

***Didelphodon*: ?**

83. Process on fibula proximal end extends proximally beyond knee:

0 absent

1 present

***Anatoliadelphys*: 0**

***Didelphodon*: ?**

*84. Sesamoids in articular area between tibia, fibula, and astragalus (or UAJ, upper ankle joint):

0 absent

1 one sesamoid present

2 two sesamoids present

***Anatoliadelphys*: ?**

***Didelphodon*: ?**

85. Tibia medial malleolus:

0 absent

1 present

***Anatoliadelphys*: 1**

***Didelphodon*: ?**

86. Tibia length relative to femur length:

0 tibia shorter than femur

1 tibia longer or equal to femur

***Anatoliadelphys*: 1**

***Didelphodon*: ?**

*87. Tibia proximal dimensions:

0 larger mediolaterally than anteroposteriorly

1 equal

2 smaller mediolaterally than anteroposteriorly

***Anatoliadelphys*: 0**

***Didelphodon*: ?**

*88. Tibia posterior shelf:

0 absent

1 present but does not extend posteriorly beyond the medial astragalotibial facet

2 present and extends posteriorly beyond the medial astragalotibial facet

***Anatoliadelphys*: 1**

***Didelphodon*: ?**

89. Tibia, distal articulation type:

0 sagittal

1 spiral

***Anatoliadelphys*: 1**

***Didelphodon*: ?**

90. Tibia, posterior shelf articulation:

0 not articular

1 articular

***Anatoliadelphys*: 0**

***Didelphodon*: ?**

*91. Astragalus, angle between medial and lateral facets for tibia:

0 90 degrees

1 intermediate

2 180 degrees

***Anatoliadelphys*: ?**

***Didelphodon*: ?**

92. Astragalonavicular facet extends on medial side of head:

0 absent

1 medial

***Anatoliadelphys*: ?**

***Didelphodon*: ?**

93. Astragalonavicular facet extends on ventromedial area of head:

0 absent

1 present

***Anatoliadelphys*: ?**

***Didelphodon*: ?**

94. Astragalus, dimensions of facet for navicular in distal view:

0 transversely wider

1 dorsoventrally wider

***Anatoliadelphys*: ?**

***Didelphodon*: ?**

95. Astragalar medial plantar tuberosity (ampt) visibility in dorsal view:

0 not visible

1 visible

***Anatoliadelphys*: ?**

***Didelphodon*: ?**

*96. Astragalus, angle between facet for fibula and lateral facet for tibia:

0 180 or more degrees

1 intermediate (90-180 degrees)

2 90 degrees

***Anatoliadelphys*: ?**

***Didelphodon*: ?**

97. Astragalar neck:

0 absent

1 present

***Anatoliadelphys*: ?**

***Didelphodon*: ?**

98. Astragalus, relative width of head and neck:

0 neck narrower or as wide as head

1 neck wider than head

***Anatoliadelphys*: ?**

***Didelphodon*: ?**

99. Astragalar sustentacular facet medial extent:

0 does not reach medial edge of neck

1 reaches medial edge of neck

***Anatoliadelphys*: ?**

***Didelphodon*: ?**

100. Astragalonavicular facet position relative to facets for tibia:

0 anterior to facets for tibia

1 medial relative to facets for tibia

***Anatoliadelphys*: ?**

***Didelphodon*: ?**

101. Astragalar canal:

0 absent

1 present

***Anatoliadelphys*: ?**

***Didelphodon*: ?**

102. Ectal facet major dimension orientation:

0 straight

1 posteromedial to anterolateral

***Anatoliadelphys*: ?**

***Didelphodon*: ?**

103. Ectal facet position in ventral view:

0 extending up to posterior edge of astragalus

1 subterminal

***Anatoliadelphys*: ?**

***Didelphodon*: ?**

104. Astragalus, ridge between medial and lateral astragalotibial facets:

0 absent

1 present

***Anatoliadelphys*: ?**

***Didelphodon*: ?**

105. Astragalus, ridge between lateral astragalotibial and astragalofibular facets:

0 absent

1 present

***Anatoliadelphys*: ?**

***Didelphodon*: ?**

106. Medial astragalotibial facet, posterior extent:

0 does not reach posterior edge of lateral astragalotibial facet

1 equal in anteroposterior length as lateral astragalotibial facet

***Anatoliadelphys*: ?**

***Didelphodon*: ?**

107. Astragalonavicular facet connection with sustentacular facet:

0 absent

1 present

***Anatoliadelphys*: ?**

***Didelphodon*: ?**

*108. Calcaneal sustentacular facet on sustentaculum:

0 no sustentaculum (facet is located in calcaneal body)

1 on sustentaculum

2 facet is located above level of sustentaculum, which becomes the medial process

***Anatoliadelphys*: 1**

***Didelphodon*: 1**

*109. Ectal facet longest dimension:

0 anteromedial to posterolateral

1 straight

2 posteromedial to anterolateral

***Anatoliadelphys*: 1**

***Didelphodon*: 2**

110. Ectal facet longest axis when straight:

0 sagittally longer

1 transversely longer

***Anatoliadelphys*: 0**

***Didelphodon*: -**

111. Calcaneal anterior peroneal tubercle:

0 absent

1 present

***Anatoliadelphys*: 1**

***Didelphodon*: 1**

*112. Calcaneal anterior peroneal tubercle shape:

0 protuberance

1 laminar

2 process

***Anatoliadelphys*: 1**

***Didelphodon*: ?**

*113. Calcaneal anterior peroneal tubercle position:

0 protruding anteriorly beyond calcaneocuboid facet

1 anterior, non-protruding

2 at a distance from anterior end of calcaneum

***Anatoliadelphys*: 1**

***Didelphodon*: 2**

114. Calcaneal sustentacular facet (anterior part) mesiolateral orientation:

0 medial

1 mediodorsal or dorsal

***Anatoliadelphys*: 1**

***Didelphodon*: 0**

115. Calcaneal sustentacular facet anteroposterior orientation:

0 dorsal

1 45 degrees dorsoanteriorly

***Anatoliadelphys*: 1**

***Didelphodon*: 1**

116. Calcaneal sustentacular facet anteroposterior convexity:

0 concave or flat

1 convex

***Anatoliadelphys*: 0**

***Didelphodon*: ?**

117. Calcaneal sustentacular facet posteriorly convex:

0 absent

1 present

***Anatoliadelphys*: ?**

***Didelphodon*: ?**

118. Calcaneal plantar tubercle:

0 absent

1 present

***Anatoliadelphys*: 1**

***Didelphodon*: ?**

*119. Calcaneal sustentacular facet and ectal facet merging:

0 separate

1 with narrow connection

2 merged

***Anatoliadelphys*: 0**

***Didelphodon*: 0**

120. Calcaneal sustentacular facet anterior edge:

0 anterior to posterior facet

1 equal or posterior to posterior facet

***Anatoliadelphys*: 0**

***Didelphodon*: 0**

121. Calcaneal facet for fibula:

0 absent

1 present

***Anatoliadelphys*: ?**

***Didelphodon*: 1**

*122. Calcaneal facet for fibula orientation:

0 lateral

1 dorsal

2 dorsal only posteriorly

***Anatoliadelphys*: ?**

***Didelphodon*: 1**

123. Calcaneocuboid facet of the calcaneum:

0 not subdivided into separate facets

1 subdivided into distal (CaCud) and proximal (CaCup) facets

2 subdivided into auxiliary (CaCua), lateral (CaCul) and medial facets

***Anatoliadelphys*: 0**

***Didelphodon*: 0**

124. Calcaneum sustentacular facet reaches anterior end:

0 absent

1 present

***Anatoliadelphys*: 1**

***Didelphodon*: 0**

125. Calcaneum accessory facet anterior to sustentacular facet:

0 absent

1 present

***Anatoliadelphys*: 0**

***Didelphodon*: 0**

126. Cuboid medial plantar process forms groove:

0 absent

1 present

***Anatoliadelphys*: ?**

***Didelphodon*: ?**

127. Cuboidcalcaneal facet:

0 mostly convex or flat

1 concave anteroposteriorly

2 two shelves, medial more proximal

3 two concavities

***Anatoliadelphys*: ?**

***Didelphodon*: ?**

*128. Cuboidcalcaneal facet angle between proximal and distal facet areas:

0 no angle

1 angle present and small, dorsal area narrower than proximal area

2 angle present and almost straight, dorsal area wider than width of proximal area

***Anatoliadelphys*: ?**

***Didelphodon*: ?**

129. Cuboidcalcaneal facet outer shelf:

0 absent

1 present

***Anatoliadelphys*: ?**

***Didelphodon*: ?**

130. Cuboidcalcaneal ventral facet:

0 absent

1 present

***Anatoliadelphys*: ?**

***Didelphodon*: ?**

131. Spatial relationship between navicular and entocuneiform:

0 entocuneiform anterior to navicular

1 entocuneiform extends proximally medial to the distal area of the navicular

***Anatoliadelphys*: ?**

***Didelphodon*: ?**

132. Navicular shelf between cuboid and astragalus:

0 absent

1 present

***Anatoliadelphys*: ?**

***Didelphodon*: ?**

*133. Navicular size:

0 navicular half to one third the size of the cuboid

1 navicular more than half the size of the cuboid to equal to it

2 navicular larger than the cuboid

***Anatoliadelphys*: ?**

***Didelphodon*: ?**

134. Mesocuneiform contact with navicular:

0 absent

1 present

***Anatoliadelphys*: ?**

***Didelphodon*: ?**

135. Falcula on hallux:

0 absent

1 present

***Anatoliadelphys*: ?**

***Didelphodon*: ?**

136. Prehallux:

0 absent

1 present

***Anatoliadelphys*: ?**

***Didelphodon*: ?**

137. Mt IV proximal contact:

0 ectocuneiform and cuboid

1 cuboid

***Anatoliadelphys*: ?**

***Didelphodon*: ?**

138. Mt V proximal process extends ventral to cuboid:

0 absent

1 present

***Anatoliadelphys*: ?**

***Didelphodon*: ?**

*139. Mt II and Mt III proximal ends:

0 Mt II extends more proximally than Mt III

1 equal

2 Mt III more proximal

***Anatoliadelphys*: ?**

***Didelphodon*: ?**

140. Hallux opposability (articulation Mt I with entocuneiform):

0 not opposable

1 opposable

***Anatoliadelphys*: ?**

***Didelphodon*: ?**

141. Ridge on proximal articular facet of Mt I:

0 absent

1 present

***Anatoliadelphys*: ?**

***Didelphodon*: ?**

142. Syndactyly or external fusion of digits II and III in the foot:

0 absent

1 present

***Anatoliadelphys*: ?**

***Didelphodon*: ?**

143. Mt III thickness relative to that of Mt IV:

0 Mt III thinner

1 Mt III and IV equal thickness or Mt IV thicker

***Anatoliadelphys*: ?**

***Didelphodon*: ?**

*144. Mt III thickness relative to that of MtI:

0 Mt III thinner

1 Mt III and I equal

2 Mt III thicker than Mt I

3 Mt I absent

***Anatoliadelphys*: ?**

***Didelphodon*: ?**

*145. Foot ungual phalanx of digit IV, proximal view:

0 larger dorsoventrally than mediolaterally

1 equal dimensions

2 larger mediolaterally than dorsoventrally

***Anatoliadelphys*: ?**

***Didelphodon*: ?**

*146. Number of upper incisors:

0 five

1 four

2 three

3 two

4 one

5 none

***Anatoliadelphys*: ?**

***Didelphodon*: 1**

*147. Number of lower incisors:

0 four

1 three

2 two

3 one

4 none

***Anatoliadelphys*: ?**

***Didelphodon*: 1**

*148. Number of upper molars:

0 four

1 three

2 none

***Anatoliadelphys*: 0**

***Didelphodon*: 0**

149. Upper molar M2 shape:

0 triangular or semi-triangular

1 rectangular or semi-square

***Anatoliadelphys*: 0**

***Didelphodon*: 0**

150. Paracone and metacone placement in M2:

0 medial or buccal

1 buccal margin

***Anatoliadelphys*: 0**

***Didelphodon*: 0**

*151. Paracone versus metacone size in M2:

0 pa > me

1 pa = me

2 pa < me

3 pa entirely suppressed

***Anatoliadelphys*: 2**

***Didelphodon*: 2**

152. Centrocrista shape:

0 linear, oriented anteroposteriorly

1 V-shaped

2 linear, oriented obliquely

***Anatoliadelphys*: 1**

***Didelphodon*: 1**

153. Metaconule:

0 absent or not-well developed

1 well-developed or enlarged

***Anatoliadelphys*: 0**

***Didelphodon*: ?**

154. Trigonid versus talonid width:

0 trigonid wider than talonid

1 trigonid subequal to talonid or trigonid narrower than talonid

***Anatoliadelphys*: 0**

***Didelphodon*: 1**

155. Paraconid on lower molars:

0 absent or tiny

1 present

***Anatoliadelphys*: 0**

***Didelphodon*: 1**

NOTES: this character has been modified from previous studies. Although a paraconid can be identified in *Anatoliadelphys*, it is tiny (far smaller than is typical for tribosphenic mammals), and so this taxon has been scored as state 0 here.

156. Intersection of cristid obliqua with trigonid on m2:

0 lingual to protocristid notch

1 labial to protocristid notch

***Anatoliadelphys*: 1**

***Didelphodon*: 1**

*157. Upper incisor arcade shape:

0 U-shape

1 broad V-shape

2 long, narrow V-shape

***Anatoliadelphys*: ?**

***Didelphodon*: 0**

158. P1:

0 absent

1 present to greatly reduced

***Anatoliadelphys*: 1**

***Didelphodon*: 1**

*159. Lower p2:

0 absent

1 greatly reduced

2 present

***Anatoliadelphys*: 2**

***Didelphodon*: 2**

*160. Upper P2:

0 absent

1 greatly reduced

2 present

***Anatoliadelphys*: 2**

***Didelphodon*: 2**

161. Upper incisors spatulate:

0 no

1 yes

***Anatoliadelphys*: ?**

***Didelphodon*: ?**

*162. Size upper I3 vs I2:

0 I3 > I2

1 I3 = I2

2 I3 < I2

***Anatoliadelphys*: ?**

***Didelphodon*: 1**

163. Procumbent gliriform lower anteriormost incisor:

0 absent

1 present

***Anatoliadelphys*: ?**

***Didelphodon*: 0**

164. Hypoconulid absent or present:

0 absent

1 present

***Anatoliadelphys*: 1**

***Didelphodon*: 1**

*165. Lower canine:

0 absent

1 greatly reduced

2 retained

***Anatoliadelphys*: 2**

***Didelphodon*: 2**

166. Upper canine:

0 caniniform or premolariform

1 reduced or absent

***Anatoliadelphys*: 0**

***Didelphodon*: 0**

167. Number of roots on upper canine:

0 two

1 one

***Anatoliadelphys*: 1**

***Didelphodon*: 1**

*168. Lower i2 (i3 of Hershkovitz) staggered or not staggered:

0 not staggered

1 staggered

***Anatoliadelphys*: ?**

***Didelphodon*: ?**

169. Bunolophodonty or lophodonty developed:

0 no

1 yes

***Anatoliadelphys*: 0**

***Didelphodon*: 0**

170. Selenodonty developed:

0 no

1 yes

***Anatoliadelphys*: 0**

***Didelphodon*: 0**

171. Marsupial pattern of dental replacement:

0 absent

1 present

***Anatoliadelphys*: ?**

***Didelphodon*: 1**

172. Parietal-alisphenoid or squamosal-frontal contact on braincase:

0 parietal-alisphenoid

1 squamosal-frontal

***Anatoliadelphys*: ?**

***Didelphodon*: 0**

173. Width of frontals versus width of parietals:

0 parietal wider or equal to frontal

1 parietal narrower than frontal

***Anatoliadelphys*: ?**

***Didelphodon*: ?**

174. Angular process medially inflected:

0 no

1 yes

***Anatoliadelphys*: 1**

***Didelphodon*: 1**

175. Mandibular symphysis fused:

0 no

1 yes

***Anatoliadelphys*: 0**

***Didelphodon*: 0**

176. Bones surrounding infraorbital canal in the orbit:

0 maxilla + lacrimal

1 maxilla only

***Anatoliadelphys*: 0**

***Didelphodon*: ?**

177. Posterior-most point of premaxillo-nasal contact:

0 anterior or at the canine

1 posterior to the canine

***Anatoliadelphys*: ?**

***Didelphodon*: 1**

178. Maximum maxilla (palatal portion) length/width ratio:

0 ratio less or equal to 15

1 ratio larger than 15

***Anatoliadelphys*: ?**

***Didelphodon*: ?**

179. Maxillofrontal contact:

0 absent

1 present

***Anatoliadelphys*: ?**

***Didelphodon*: ?**

180. Lacrimal tubercle:

0 absent

1 present

***Anatoliadelphys*: ?**

***Didelphodon*: ?**

*181. Alisphenoid tympanic wing:

0 absent

1 poorly developed

2 moderately developed

3 well-developed, extending to or near posterior lacerate foramen and paroccipital process

***Anatoliadelphys*: ?**

***Didelphodon*: 2**

*182. Ectotympanic shape:

0 ring-shaped

1 moderately broadened

2 tubelike

***Anatoliadelphys*: ?**

***Didelphodon*: ?**

183. Postglenoid process:

0 absent

1 present

***Anatoliadelphys*: 1**

***Didelphodon*: 1**

184. Bony external auditory meatus separates ear canal from epitympanic recess:

0 no

1 yes

***Anatoliadelphys*: ?**

***Didelphodon*: ?**

185. Fusion of ectotympanic with other bones of the skull:

0 no

1 yes

***Anatoliadelphys*: ?**

***Didelphodon*: ?**

186. Postglenoid foramen:

0 absent

1 present

***Anatoliadelphys*: 1**

***Didelphodon*: 1**

*187. Postglenoid foramen position:

0 located posterior to postglenoid process

1 even with postglenoid process

2 anterior to postglenoid process and frequently encircled by squamosal

***Anatoliadelphys*: 0**

***Didelphodon*: 0**

188. Position of incisura tympanica:

0 caudal or caudodorsal

1 located dorsally or anterior crus and posterior crus unite dorsally

***Anatoliadelphys*: ?**

***Didelphodon*: ?**

189. Size of incisura tympanica:

0 narrow or absent

1 wide

***Anatoliadelphys*: ?**

***Didelphodon*: ?**

190. Foramen ovale position:

0 lamina obturans / other bones

1 alisphenoid / petrosal

2 just alisphenoid

3 alisphenoid / squamosal

***Anatoliadelphys*: ?**

***Didelphodon*: 2/3**

191. Carotid foramen position:

0 in basisphenoid

1 basisphenoid / basioccipital suture

2 basisphenoid / petrosal

***Anatoliadelphys*: ?**

***Didelphodon*: 2**

192. Transverse canal foramen:

0 absent

1 present

***Anatoliadelphys*: 1**

***Didelphodon*: ?**

193. Transverse canal position:

0 anterior to carotid foramen

1 perforating pterygoid fossa

2 confluent with carotid foramen

3 posterior to carotid foramen

***Anatoliadelphys*: 0**

***Didelphodon*: ?**

194. Intramural transverse canal:

0 absent

1 present

***Anatoliadelphys*: 1**

***Didelphodon*: ?**

195. Dorsal margin of foramen magnum:

0 formed by exoccipitals and supraoccipital

1 formed by exoccipitals

***Anatoliadelphys*: ?**

***Didelphodon*: ?**

196. Shape of nasals:

0 posteriorly expanded

1 not posteriorly expanded

***Anatoliadelphys*: ?**

***Didelphodon*: 0**

197. Septomaxilla:

0 absent

1 present

***Anatoliadelphys*: ?**

***Didelphodon*: 0**

*198. Palatal vacuities:

0 absent or just small foramina

1 present, restricted to palatine bones

2 present in both palatine and maxillary bones

***Anatoliadelphys*: ?**

***Didelphodon*: 2**

199. Premaxilla, palatal process:

0 does not

1 does reach canine alveolus or it is immediately posterior to it

***Anatoliadelphys*: ?**

***Didelphodon*: 1**

200. Minor palatine foramen:

0 absent

1 present

***Anatoliadelphys*: ?**

***Didelphodon*: 1**

*201. Hypoglossal foramina:

0 confluent with jugular foramen

1 one

2 two or more

***Anatoliadelphys*: ?**

***Didelphodon*: ?**

202. Optic foramen:

0 absent

1 present

***Anatoliadelphys*: ?**

***Didelphodon*: ?**

203. Masseteric canal and dental canal in dentary:

0 masseteric canal does not open into dental canal

1 masseteric canal opens into dental canal

***Anatoliadelphys*: 0**

***Didelphodon*: 0**

204. Malleolar neck:

0 long relative to head and manubrium

1 short

***Anatoliadelphys*: ?**

***Didelphodon*: ?**

*205. Ossicular axis:

0 > 20 degrees

1 10 to 20 degrees

2 < 10 degrees

***Anatoliadelphys*: ?**

***Didelphodon*: ?**

206. Manubrial-incudal lever-arm ratio:

0 equal or smaller than 16

1 larger than 16

***Anatoliadelphys*: ?**

***Didelphodon*: ?**

207. Stapedial ratio:

0 < 18

1 > 18

***Anatoliadelphys*: ?**

***Didelphodon*: ?**

208. Stapedial foramen visible:

0 absent

1 present

***Anatoliadelphys*: ?**

***Didelphodon*: ?**

209. Bullate stapes:

0 not bullate

1 bullate

***Anatoliadelphys*: ?**

***Didelphodon*: ?**

210. Paraseptal cartilage dips down vertically by the side of nasopalatine duct:

0 no

1 yes

***Anatoliadelphys*: ?**

***Didelphodon*: ?**

211. Paraseptal cartilage shape:

0 outer bar connects with uppermost portion of paraseptal cartilage

1 with middle portion, dorsal process short

2 with middle portion, dorsal process long

***Anatoliadelphys*: ?**

***Didelphodon*: ?**

212. Portion of paraseptal cartilage is ring-shaped in cross section:

0 no

1 yes

***Anatoliadelphys*: ?**

***Didelphodon*: ?**

213. Sperm pairing in epididymis:

0 no

1 yes

***Anatoliadelphys*: ?**

***Didelphodon*: ?**

214. Pouch type in mammary area:

0 type 1

1 type 5

2 type 6

3 no marsupium or skin folds develop during the reproductive period

4 thin marsupium-like structure develops during reproductive period

***Anatoliadelphys*: ?**

***Didelphodon*: ?**

*215. Mammary count:

0 0 teats

1 2 teats

2 4 teats

3 5-8 teats

4 9 teats or more

***Anatoliadelphys*: ?**

***Didelphodon*: ?**

216. Caecum:

0 absent

1 present

***Anatoliadelphys*: ?**

***Didelphodon*: ?**

217. Fasciculus aberrans in brain:

0 absent

1 present

***Anatoliadelphys*: ?**

***Didelphodon*: ?**

218. Cavum supracochleare:

0 is not roofed dorsally by the petrosal, and a depression in the anterior lamina for the geniculate ganglion is visible in dorsal view

1 is entirely enclosed within the petrosal

2 or is not floored ventrally, such that there is no secondary facial foramen

***Anatoliadelphys*: ?**

***Didelphodon*: 1**

219. Cavum epiptericum floored by:

0 petrosal

1 petrosal and alisphenoid

2 primarily or exclusively by alisphenoid

3 primarily open as piriform fenestra

***Anatoliadelphys*: ?**

***Didelphodon*: 2**

220. Fossa subarcuata:

0 deep

1 extremely shallow

***Anatoliadelphys*: 0**

***Didelphodon*: 0**

221. Pars mastoidea:

0 not extensively pneumatised

1 extensively pneumatised and composed of cancellous bone

***Anatoliadelphys*: 0**

***Didelphodon*: 0**

222. Expansion of the crista petrosa into a salient crest that may cover the anterolateral part of the fossa subarcuata:

0 absent

1 present

***Anatoliadelphys*: ?**

***Didelphodon*: ?**

223. Anterior lamina of petrosal exposure on the lateral wall of the braincase:

0 present and large

1 rudimentary

2 absent

***Anatoliadelphys*: ?**

***Didelphodon*: 2**

224. Anterior lamina of petrosal:

0 makes a major contribution to the medial wall of the middle cranial fossa

1 makes a minor contribution

***Anatoliadelphys*: ?**

***Didelphodon*: ?**

225. Internal acoustic meatus:

0 with prefacial commissure at least 50% the width of the internal acoustic meatus

1 with prefacial commissure less than 50% the width of the internal acoustic meatus

***Anatoliadelphys*: ?**

***Didelphodon*: ?**

226. Deep groove for internal carotid artery excavated on anterior pole of promontorium:

0 absent

1 present

***Anatoliadelphys*: ?**

***Didelphodon*: 0**

227. Deep and large fossa for the tensor tympani muscle excavated on the anterolateral aspect of promontorium, creating a battered ventral surface of the promontorium:

0 absent

1 present

***Anatoliadelphys*: ?**

***Didelphodon*: 0**

228. Epitympanic wing of petrosal (1):

0 absent

1 present

***Anatoliadelphys*: ?**

***Didelphodon*: 1**

229. Epitympanic wing of petrosal (2):

0 flat

1 confluent with bulla

***Anatoliadelphys*: ?**

***Didelphodon*: ?**

230. Lateral flange:

0 large and lateral to promontorium

1 greatly reduced or absent

***Anatoliadelphys*: ?**

***Didelphodon*: 1**

231. Broad shelf of bone surrounding fenestra cochleae and making a separation between it and aqueductus cochleae:

0 absent

1 present

***Anatoliadelphys*: ?**

***Didelphodon*: 0**

232. Rostral tympanic process of petrosal (1):

0 absent

1 present as a distinct crest or erected process

***Anatoliadelphys*: ?**

***Didelphodon*: ?**

233. Rostral tympanic process of petrosal (2):

0 forms an anterolaterally directed wing, sometimes contacting the ectotympanic, that does not extend on the whole length of the promontorium

1 that does extend on the whole length of the promontorium

***Anatoliadelphys*: ?**

***Didelphodon*: ?**

*234. Tympanic aperture of hiatus Fallopii:

0 dorsal

1 intermediate

2 ventral

***Anatoliadelphys*: ?**

***Didelphodon*: ?**

235. Stylomastoid foramen:

0 absent

1 present

***Anatoliadelphys*: ?**

***Didelphodon*: 1**

236. Inferior petrosal sinus:

0 intrapetrosal

1 between petrosal, basisphenoid and basioccipital

2 endocranial

***Anatoliadelphys*: ?**

***Didelphodon*: ?**

237. Mastoid exposure:

0 contacts the parietal

1 does not contact the parietal

2 greatly reduced, forming a dorsoventrally narrow band on the ventrolateral corner of the posterior face of the cranium

***Anatoliadelphys*: 0/1**

***Didelphodon*: 0/1**

238. Mastoid tympanic process:

0 large and vertical

1 small, slanted, and nodelike, on the posterolateral border of the stylomastoid notch and continuous with squamosal

2 indistinct to absent

***Anatoliadelphys*: ?**

***Didelphodon*: ?**

239. Caudal tympanic process of petrosal (1):

0 absent

1 present

***Anatoliadelphys*: ?**

***Didelphodon*: 1**

240. Caudal tympanic process of petrosal (2):

0 forms a small crest that does not wholly floor the postpromontorial sinus

1 forms an expanded lamina that floors the postpromontorial sinus

***Anatoliadelphys*: ?**

***Didelphodon*: ?**

241. Petrosal plate:

0 absent

1 present

***Anatoliadelphys*: ?**

***Didelphodon*: 0**

242. Fossa incudis and epitympanic recess:

0 continuous

1 separated by a distinct ridge

***Anatoliadelphys*: ?**

***Didelphodon*: 1**

243. Petrosal crest:

0 absent

1 present

***Anatoliadelphys*: ?**

***Didelphodon*: ?**

244. Petrosal contribution to the lateral wall of the epitympanic recess (1):

0 absent

1 present

***Anatoliadelphys*: ?**

***Didelphodon*: ?**

245. Petrosal contribution to the lateral wall of the epitympanic recess (2):

0 massive, large shelf of bone, sometimes rounded

1 slender and triangular

2 forming a thin lamina

***Anatoliadelphys*: ?**

***Didelphodon*: ?**

246. Prootic canal:

0 present

1 absent

***Anatoliadelphys*: ?**

***Didelphodon*: ?**

247. Imprint of the transverse sinus bifurcation on the petrosal:

0 absent

1 present

***Anatoliadelphys*: ?**

***Didelphodon*: ?**

248. Foramina on the sigmoid sinus and/or prootic sinus, apparently connecting both vessels (i.e. sigmoid sinus vein):

0 absent

1 present

***Anatoliadelphys*: ?**

***Didelphodon*: ?**

249. Posttemporal sulcus on the squamosal surface of the petrosal:

0 present

1 absent

***Anatoliadelphys*: ?**

***Didelphodon*: 0**

250. Posttemporal notch/foramen:

0 present

1 absent

***Anatoliadelphys*: ?**

***Didelphodon*: ?**

251. Transpromontorial sulcus:

0 present

1 absent

***Anatoliadelphys*: ?**

***Didelphodon*: 1**

252. Sulcus for stapedial artery:

0 present

1 absent

***Anatoliadelphys*: ?**

***Didelphodon*: 1**

253. Cochlear coiling:

0 absent or less than 300°

1 fully coiled (more than 360°)

***Anatoliadelphys*: ?**

***Didelphodon*: 1**

254. Tympanic sinus formed in the lateral trough (or anterolateral expansion of the pars canalicularis):

0 absent

1 present

***Anatoliadelphys*: ?**

***Didelphodon*: ?**

255. Squamosal contribution to hypotympanic sinus roof:

0 absent

1 present

***Anatoliadelphys*: ?**

***Didelphodon*: ?**

256. Alisphenoid contribution to hypotympanic sinus roof:

0 absent

1 present

***Anatoliadelphys*: ?**

***Didelphodon*: ?**

257. Posterior cingulid on lower molars:

0 absent

1 present

***Anatoliadelphys*: 0+1**

***Didelphodon*: 1**

258. Distinct third trochanter:

0 present

1 absent

***Anatoliadelphys*: 0**

***Didelphodon*: ?**

259. Squamosal epitympanic sinus:

0 absent

1 present

***Anatoliadelphys*: ?**

***Didelphodon*: ?**

References

1. Beck RMD, Travouillon KJ, Aplin KP, Godthelp H, Archer M. The osteology and systematics of the enigmatic Australian Oligo-Miocene metatherian *Yalkaparidon* (Yalkaparidontidae; Yalkaparidontia; ?Australidelphia; Marsupialia). J Mamm Evol. 2014;21(2):127-72.

2. Horovitz I, Sánchez-Villagra MR. A morphological analysis of marsupial mammal higher-level phylogenetic relationships. Cladistics. 2003;19:181-212.

3. Sánchez-Villagra MR, Ladevèze S, Horovitz I, Argot C, Hooker JJ, Macrini TE, et al. Exceptionally preserved North American Paleogene metatherians: adaptations and discovery of a major gap in the opossum fossil record. Biol Lett. 2007;3(3):318-22.

4. Beck RMD, Godthelp H, Weisbecker V, Archer M, Hand SJ. Australia’s oldest marsupial fossils and their biogeographical implications. PLoS ONE. 2008;3(3):e1858. doi: 10.1371/journal.pone.0001858.

5. Horovitz I, Ladevèze S, Argot C, Macrini TE, Martin T, Hooker JJ, et al. The anatomy of *Herpetotherium* cf. *fugax* Cope, 1873, a metatherian from the Oligocene of North America. Palaeontographica Abteilung A. 2008;284(4-6):109-41.

6. Horovitz I, Martin T, Bloch J, Ladevèze S, Kurz C, Sánchez-Villagra MR. Cranial anatomy of the earliest marsupials and the origin of opossums. PLoS ONE. 2009;4(12):e8278.

7. Abello MA, Candela AM. Postcranial skeleton of the Miocene marsupial *Palaeothentes* (Paucituberculata, Palaeothentidae): paleobiology and phylogeny. J Vertebr Paleontol. 2010;30(5):1515–27. doi: 10.1080/02724634.2010.501437. PubMed PMID: WOS:000281874900016.

8. Beck RMD. An 'ameridelphian' marsupial from the early Eocene of Australia supports a complex model of Southern Hemisphere marsupial biogeography. Naturwissenschaften. 2012;99(9):715-29. Epub 2012/08/07. doi: 10.1007/s00114-012-0953-x. PubMed PMID: 22864962.
