# Supplementary material for: Skeleton of an unusual, cat-sized marsupial relative (Metatheria: Marsupialiformes) from the middle Eocene (Lutetian: 44-43 million years ago) of Turkey
Source: PLoS One. 2017 Aug 16;12(8):e0181712. doi: 10.1371/journal.pone.0181712 (PMC5559079; doi:10.1371/journal.pone.0181712)
Supplement: S3 Text — (DOCX) [file pone.0181712.s004.docx]

**S3 Text. Assumed age ranges (MYA) for fossil taxa included in phylogenetic analysis**

These age ranges were assumed for the fossil taxa in the “tip-and-node dating” analysis, with the ages of modern taxa assumed to be 0 MYA. Beck and Lee [1] provided detailed justification for the ages of several taxa, based on published sources.

| Taxon | Age range (MYA) | Reference(s) |
| --- | --- | --- |
| *Vincelestes* | 132.9-125.0 | [1] |
| *Ukhaatherium* | 83.8-71.9 | [1] |
| *Asioryctes* | 83.8-71.9 | [1] |
| *Deltatheridium* | 83.8-71.9 | [1] |
| *Mayulestes* | 66.0-59.2 | [1] |
| *Pucadelphys* | 66.0-59.2 | [1] |
| *Andinodelphys* | 66.0-59.2 | [1] |
| *Asiatherium* | 83.8-71.9 | [1] |
| *Herpetotherium* | 33.9-33.3 | [1] |
| Peradectidae | 54.7-54.4 | [2] |
| *Djarthia* | 54.65-54.55 | [3, 4] |
| *Palaeothentes* | 19.0-14.0 | [5, 6] |
| *Anatoliadelphys* | 44.0-43.0 | [7] |
| *Didelphodon* | 69.0-66.0 | [8] |

References

1. Beck RMD, Lee MSY. Ancient dates or accelerated rates? Morphological clocks and the antiquity of placental mammals. Proceedings of the Royal Society B: Biological Sciences. 2014;281:20141278. doi: 10.1098/rspb.2014.1278. PubMed PMID: 25165770.

2. Horovitz I, Martin T, Bloch J, Ladevèze S, Kurz C, Sánchez-Villagra MR. Cranial anatomy of the earliest marsupials and the origin of opossums. PLoS ONE. 2009;4(12):e8278.

3. Godthelp H, Archer M, Cifelli RL, Hand SJ, Gilkeson CF. Earliest known Australian Tertiary mammal fauna. Nature. 1992;356:514-6.

4. Beck RMD, Godthelp H, Weisbecker V, Archer M, Hand SJ. Australia’s oldest marsupial fossils and their biogeographical implications. PLoS ONE. 2008;3(3):e1858. doi: 10.1371/journal.pone.0001858.

5. Abello MA, Candela AM. Postcranial skeleton of the Miocene marsupial *Palaeothentes* (Paucituberculata, Palaeothentidae): paleobiology and phylogeny. J Vertebr Paleontol. 2010;30(5):1515–27. doi: 10.1080/02724634.2010.501437. PubMed PMID: WOS:000281874900016.

6. Perkins ME, Fleagle JG, Heizler MT, Nash B, Bown TM, Tauber AA, et al. Tephrochronology of the Miocene Santa Cruz and Pinturas Formations, Argentina. In: Vizcaíno SF, Kay RF, Bargo MS, editors. Early Miocene paleobiology in Patagonia: high-latitude paleocommunities of the Santa Cruz Formation. Cambridge: Cambridge University Press; 2012. p. 23-40.

7. Licht A, Coster P, Ocakoğlu F, Campbell C, Métais G, Mulch A, et al. Tectono-stratigraphy of the Orhaniye Basin, Turkey: Implications for collision chronology and Paleogene biogeography of central Anatolia. J Asian Earth Sci. in press.

8. Wilson GP, Ekdale EG, Hoganson JW, Calede JJ, Vander Linden A. A large carnivorous mammal from the Late Cretaceous and the North American origin of marsupials. Nature communications. 2016;7:13734. doi: 10.1038/ncomms13734. PubMed PMID: 27929063; PubMed Central PMCID: PMCPMC5155139.
